# Thiofunctionalization of Silyl Enol Ether: An Efficient Approach for the Synthesis of *β*-Keto Sulfides

**DOI:** 10.3390/molecules30194032

**Published:** 2025-10-09

**Authors:** Xinyao Zhao, Hexia Ye, Yajie Fu, Haibo Liu, Xiaojing Bi

**Affiliations:** State Key Laboratory of Chemistry for NBC Hazards Protection, Beijing 102205, China; xy01253@yeah.net (X.Z.); yehexia6688@yeah.net (H.Y.); yajief2022@163.com (Y.F.)

**Keywords:** *β*-keto sulfide, ketone, silyl enol ether, catalyst-free

## Abstract

*β*-Keto sulfides are a class of compounds containing both carbonyl (C=O) and thioether (C–S–C) functionalities, exhibiting significant potential in the field of medicinal chemistry. This study employs the silyl enol ether as the substrate, enabling the formation of C–S bonds under catalyst- and additive-free conditions, thereby facilitating the efficient synthesis of *β*-keto sulfides. The reaction proceeds rapidly and efficiently, exhibiting a broad substrate scope, and a total of 31 target compounds were synthesized with up to 95% yields.

## 1. Introduction

**Figure 1:**
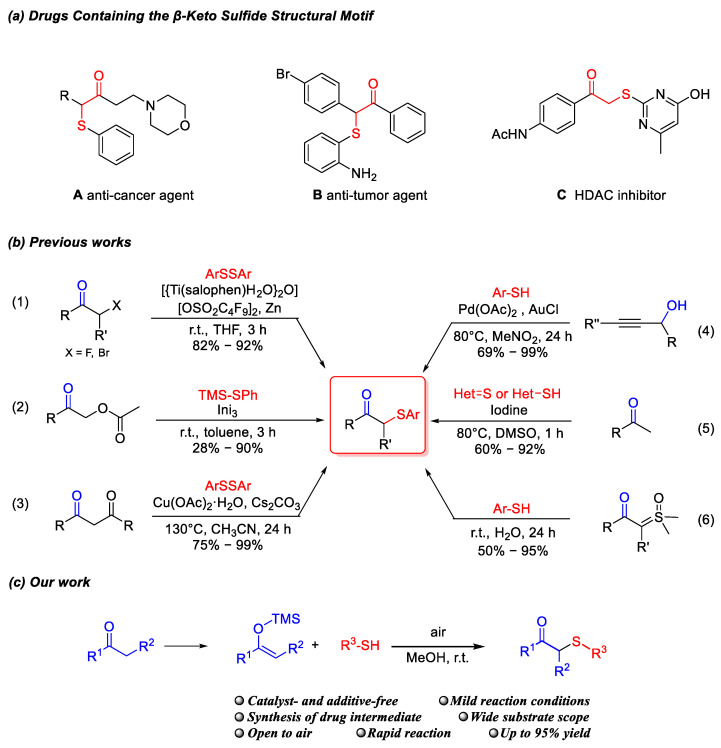
Drugs and Synthetic Strategies for *β*-Keto Sulfide Compounds.

*β*-Keto sulfides are widely found in nature and demonstrated significant potential in the field of medicinal chemistry. These compounds have long served as essential precursors in the synthesis of biologically active molecules [1,2]. Among various sulfur-containing compounds, *β*-keto sulfides represent a crucial class of bioactive molecules that exhibit diverse pharmacological properties. As potent anti-cancer and anti-tumor agents, *β*-keto sulfides can suppress tumor cell proliferation through specific interactions with biological macromolecules such as proteins and enzymes (Figure 1a(A)), or by interfering with the cell cycle and promoting apoptosis (Figure 1a(B)) [3]. Furthermore, *β*-keto sulfides are capable of inhibiting viral replication by modulating key enzymatic activities involved in the process, such as serine proteases, thereby preventing viral spread (Figure 1a(C)) [4]. Moreover, *β*-keto sulfides can function as acyl transfer reagents participating in the acetylation of proteins, a process that thereby closely modulates various intracellular pathways associated with the pharmacological efficacy of drugs within human body [5]. Therefore, the development of simple methods for the synthesis of *β*-keto sulfides with diverse functional groups is of great significance.

The most classic method for synthesizing *β*-keto sulfides involves substitution reactions utilizing α-halogenated carbonyl compounds as substrates (Figure 1b(1)) [6]. In 2012, Nishimoto Y et al. employed low-toxicity and biodegradable alkyl acetates as substrates and utilized the metal catalyst InI_3_ to promote the cyclization reaction for the synthesis of *β*-keto sulfides (Figure 1b(2)) [7]. Subsequently, in 2013, Zou L H et al. employed 1,3-dicarbonyl compounds (Figure 1b(3)) [8], and in 2014, Biswas et al. employed propargyl alcohol (Figure 1b(4)) [9] as a substrate to replace toxic halogenated carbonyl compounds such as phenacyl chloride, providing an environmentally benign alternative. In 2016, Siddaraju et al. utilized phenylacetone as substrate and applied an iodine-promoted, DMSO-oxidized cross-dehydrogenative coupling reaction to generate the target product (Figure 1b(5)) [10]. Subsequently, in 2020, Xu’s group reported that sulfonium ylides could efficiently react with aryl thiols under aqueous conditions at room temperature (Figure 1b(6)) [11].

Herein, we report a highly efficient synthesis of *β*-keto sulfides via a catalyst- and additive-free reaction between silyl enol ethers and sulfur-containing substrates in methanol under ambient air at room temperature. Furthermore, silyl enol ether intermediates can be conveniently prepared from readily available ketones with high yields (≥98%) [12]. The resulting silyl enol ether solution can be directly employed in the subsequent construction of target molecules after simple filtration and solvent removal, obviating any further isolation or purification. Trace quantities of residual ketone that may persist in the filtrate exert no measurable influence on the downstream reaction.

## 2. Results and Discussion

We propose that the sulfur source may undergo oxidation to generate a thiol radical under aerobic conditions, which could subsequently participate in an addition reaction with silyl enol ethers to form a C–S bond. To assess the feasibility of this hypothesis, we carried out a preliminary investigation employing 1-phenyl-1-trimethylsiloxyethylene (**1a**) and *p*-methylphenylthiol (**2a**) as model substrates, the results of which are summarized in Table 1.

Screening of solvents demonstrated that non-polar toluene delivered the target product in only 32% yield (Table 1, entry 1). Similarly, aprotic solvents such as MeCN, DMSO, DMF, THF and DCM (Table 1, entries 2–6), as well as polar protic solvent H_2_O (Table 1, entries 8) afforded lower yields. To our delight, using EtOH as the solvent resulted in 78% yield of the target product **3a** (entry 9), whereas MeOH afforded a significantly higher yield 95% (entry 10). In this reaction system, methanol serves not merely as a conventional solvent but also plays an active role in promoting the reaction. The small molecular size, moderate polarity, and capability to act as both a hydrogen bond donor and acceptor collectively facilitate the elimination of the trimethylsilyl (TMS) group from the enol silyl ether. By contrast, other commonly employed solvents fail to simultaneously satisfy these requirements, ultimately leads to a significant decrease in reaction efficiency. Under a nitrogen atmosphere the reaction yield decreases significantly (entry 14). The majority of *p*-methylphenylthiol (**2a**) remained unreacted, whereas 1-phenyl-1-trimethylsiloxyethylene (**1a**) was quantitatively recovered as acetophenone. This outcome can be attributed to the inability of the sulfur-containing substrate to generate a phenylthiyl radical in the absence of O_2_, thus preventing downstream C–S coupling. This hypothesis will be further investigated through radical trapping experiments.

With the optimal solvent established, the stoichiometry of the thiol was systematically examined. 1.0, 1.5 and 2.0 equiv. **2a** were performed. The reaction inevitably produces the byproduct disulfide, which cannot be entirely eliminated, and the optimal performance was achieved when 2.0 equiv. **2a** was employed (Table 1, entry 10). Time-course monitoring indicated that substrate **1a** was completely consumed within 1 h (Table 1, entry 13). Moreover, investigation of the reaction atmosphere indicated that the transformation was severely inhibited under nitrogen, thereby demonstrating that the presence of air is indispensable for the reaction to proceed efficiently.

Thus, the optimal conditions for the reaction of 1-phenyl-1-trimethylsiloxyethylene (**1a**) with *p*-methylphenylthiol (**2a**) are as follows: MeOH as solvent, 2.0 equiv. of **2a**, open to air, 1 h at ambient temperature.

**Figure 2:**
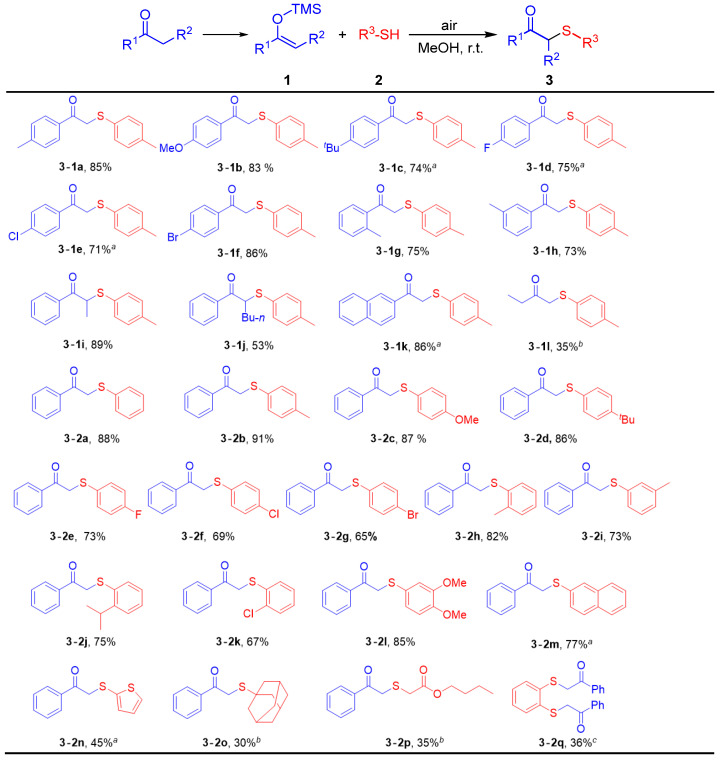
Substrate Scope of *β*-Keto Sulfides. Reaction conditions: **1** (0.6 mmol), **2** (1.2 mmol), MeOH (6 mL), r.t., air, 1 h, isolated yields.; *^a^* 4 h. *^b^* 12 h. *^c^* **1** (1.5 mmol), **2** (0.6 mmol), 6 h.

With the optimized conditions established, we proceeded to evaluated the scope of this transformation using silyl enol ethers **1** derived from ketones and thiophenols **2** as substrates (Figure 2).

To our delight, various silyl enol ethers and thiophenols bearing electron-withdrawing and electron-donating substituents on the aromatic ring proved to be competent substrates in the synthesis of the desired products (Figure 2, **3-1a**–**3-1h**, **3-2a**–**3-2k**). The silyl enol ether bearing different substituents at the terminal position were also found to be compatible, thereby enabling the formation of **3-1i** in 89% yield and **3-1j** in 53% yield. Using trimethyl((1-(naphthalen-2-yl)vinyl)oxy)silane as the starting material, a satisfactory yield was achieved (Figure 2, **3-1k**). Additionally, 1-ethyl-1-trimethylsilyloxyethenewas shown to be a slightly less efficient, providing compound **3-1l** in 35% yield. Polycyclic thiophenol provided the desired product in a good yield. (Figure 2, **3-2m**, 77%). The diminished reactivity and lower yield observed with thiophenethiol (Figure 2, **3-2n**, 45%). The main reason for this is that the electronegativity of sulfur in the thiophene ring is stronger than that of carbon in *p*-methylbenzene, resulting in a weaker electron-donating ability compared to p-methylbenzene. To our delight, both aliphatic and cyclic thiols bearing a mercapto group are capable to yield the target product (Figure 2, **3-2o**, **3-2p**). When benzene-1,2-dithiol was employed as the reactant, the addition of 2.5 eq. of **1a** results in the combination of two molecules of **1a** with the thiol group (Figure 2, **3-2q**). Overall, for different substituents on the benzene ring, electron-donating groups increase the electron cloud density, which facilitates the reaction, whereas electron-withdrawing groups decrease the electron cloud density, leading to a comparatively lower yield than that observed with electron-donating groups.

**Figure 3:**
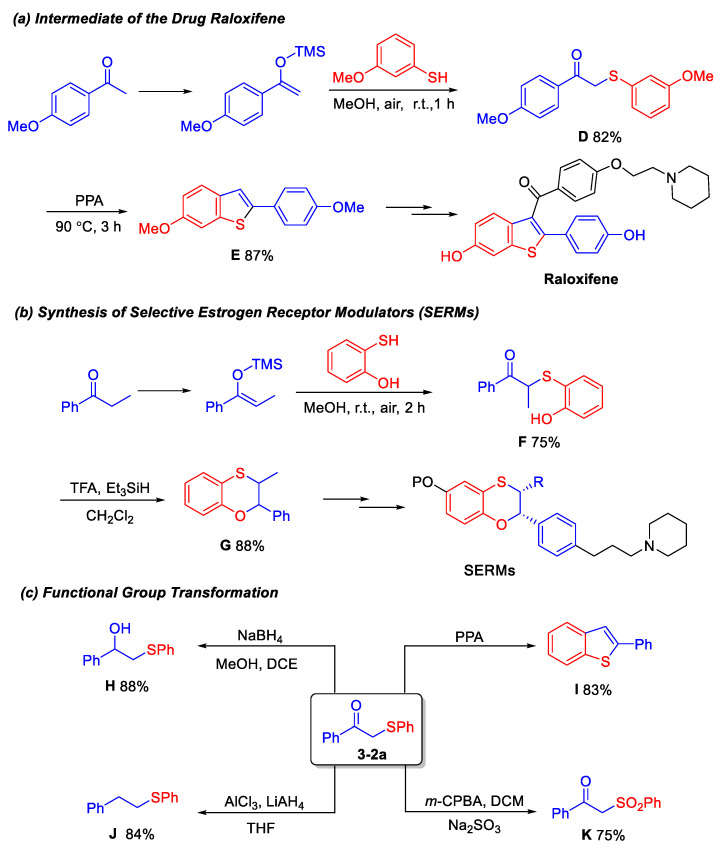
Synthetic applications and functional group transformation.

Raloxifene is a prescription medication indicated for the prevention and treatment of osteoporosis. It reduces the risk of osteoporotic fractures by inhibiting bone resorption and enhancing bone mineral density (BMD). Selective estrogen receptor modulators (SERMs) are capable of selectively binding to estrogen receptor α (ERα), thereby modulating estrogenic activity and ameliorating conditions associated with estrogen deficiency. This experimental methodology enables the facile synthesis of intermediates for the pharmaceutical agent Raloxifene (Figure 3a) [12,13,14], and selective estrogen receptor modulators SERMs (Figure 3b) [15,16]. Moreover, through a series of straightforward functional group transformations, as illustrated in Figure 3, compound **3-2a** can be readily converted into various valuable derivatives, such as the *β*-hydroxy sulfide **H**, thiophene ring **I**, thioether **J**, and sulfone **K** (Figure 3c) [17,18,19].

**Figure 4:**
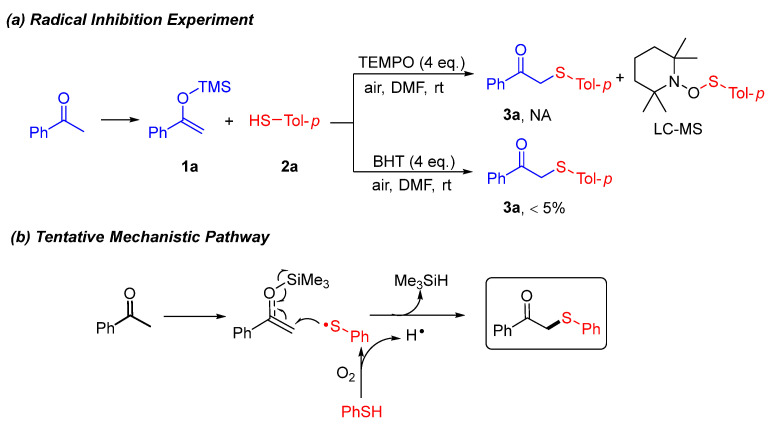
Plausible mechanism.

Subsequently, a series of mechanistic experiments were conducted to elucidate the formation pathway of *β*-keto sulfides (Figure 4a). First, upon introduction of TEMPO (4 equiv.) under standard reaction conditions, no desired product was observed, and LC–MS analysis revealed the formation of a phenylthiyl radical adduct (see figure titled “Radical inhibition experiment” in the Supplementary Materials). Similarly, the reaction was markedly inhibited upon the addition of BHT under standard reaction conditions, suggesting the possible involvement of radical intermediates in the reaction process. Based on the aforementioned experimental results and prior research, we propose a plausible reaction mechanism for this transformation (Figure 4b). Radical trapping experiments offer compelling evidence that the reaction proceeds via a radical-based mechanism. The proposed reaction mechanism is as follows. Thiyl radicals were generated under an air atmosphere from thiols [20],which couped with 1-phenyl-1-trimethylsiloxyethylene (**1a**) leading to the cleavage of the double bond and the formation of the target product with the elimination of the trimethylsilane.

## 3. Materials and Methods

Unless otherwise stated, all commercially available reagents were obtained from InnoChem (Beijing, China) and used without further purification. All reactions were carried out in a 10 mL tube under magnetic stirring and monitored by TLC, GC-MS and LC-MS. HRMS analyses of the compounds were conducted using an ultrahigh-resolution electrospray ionization time-of-flight mass spectrometer (Waters Xevo G2-XS QTOF, Waters, Medford, MA, USA) and an electron impact ionization time-of-flight mass spectrometer(Q Exactive™ GC Orbitrap™ GC-MS/MS, Thermo Fisher Scientific, Waltham, MA, USA). TLC was performed using 0.25 mm silica plates, and flash column chromatography was carried out with 200−300 mesh silica gel supplied by Qingdao Haiyang Chemical Co., Ltd. (Qingdao, China). ^1^H NMR, ^13^C NMR and ^19^F NMR spectrum were recorded on Bruker Ultrashield TM 300 MHz instruments (Bruker Corporation, Billerica, MA, USA) and were calibrated using residual undeuterated solvent (TMS δ 0.00 ppm, CDCl_3_ δ 7.26 ppm, ^1^H NMR; CDCl_3_ δ 77.16 ppm ^13^C NMR). Data were reported as follows: chemical shift, multiplicity (s = singlet, d = doublet, t = triplet, q = quartet, dd = doublet of doublets, td = triplet of doublets, qd = quartet of doublets, m = multiplet), coupling constants (Hz) and integration.

### Experimental Procedure

Synthesis of silyl enol ether [21]. Under anhydrous and oxygen-free conditions, a 10 mL glass vial was charged with acetophenone (0.6 mmol, 1.0 equiv.), trimethylchlorosilane (0.72 mmol, 1.2 equiv.) and triethylamine (1.44 mmol, 2.4 equiv.), followed by the slow addition of a NaI solution in acetonitrile (0.6 mL, 0.72 mmol, 1.2 equiv., 1.2 mmol/mL) at room temperature. The mixture was stirred at 25 °C under N_2_ conditions and monitored by TLC until the reaction was complete. The reaction was completed in approximately 40 min. The reaction mixture was filtered and washed with ethyl acetate, followed by removal of the solvent under reduced pressure at room temperature to afford the desired product.

Synthesis of *β*-Keto Sulfides. In a typical experiment procedure, a 10 mL glass vial was charged with 1-Phenyl-1-trimethylsiloxyethylene derived from acetophenone (0.6 mmol, 1.0 equiv.), thiophenol (1.2 mmol, 2 equiv.) and MeOH (6 mL). The mixture was stirred at 25 °C under ambient air conditions and monitored by TLC, GC-MS and LC-MS until the reaction was complete. H_2_O was added, and the aqueous phase was extracted with EA (3 × 10 mL). The combined organic layers were dried over anhydrous Na_2_SO_4_ and concentrated under reduced pressure to afford the crude product. The residue was purified by column chromatography on silica gel using a mixture of EA and n-hexane (1:19, *v*/*v*) as the eluent to yield the desired products.

Synthesis of 3-methyl-2-phenyl-2,3-dihydrobenzo[*b*][1,4]oxathiine (Compound **G**) [16]. **F** (0.4 mmol, 1.0 equiv.) in DCM (0.05 M) under N_2_ was cooled to 0 °C and treated dropwise with TFA (4 mmol, 10 equiv.) followed by Et_3_SiH (1.6 mmol, 4 equiv.). The mixture was stirred at 0–25 °C until TLC showed complete consumption of starting material (2–10 h), then poured into ice-cold saturated NaHCO_3_, extracted with DCM, washed with brine, dried over Na_2_SO_4_, and purified by flash chromatography (EA/n-hexanes, 1:5) to afford 3-methyl-2-phenyl-2,3-dihydrobenzo[*b*][1,4]oxathiine in 88% yield.

Synthesis of *β*-hydroxy sulfide (H) [19]. NaBH_4_ (1.0 mmol) was added to a cooled solution (0 °C) of **3-2a** (0.5 mmol) in MeOH/DCM (4/1, 10 mL). The reaction mixture was stirred at room temperature for 0.5 h and then concentrated to remove the solvent. After that, the reaction was quenched by the addition of saturated aqueous NH_4_Cl (20.0 mL), and the resulting mixture was extracted with ethyl acetate (10.0 mL × 3). The combined organic layers were washed with brine, dried over Na_2_SO_4_, and concentrated under reduced pressure. The residue was purified by column chromatography on silica gel using (EA/n-hexanes, 1:8) as the eluent to afford the *β*-hydroxy sulfide.

Synthesis of benzothiophene (I) [14]. Polyphosphoric acid (2 mL) was heated to 90 °C and **3-2a** (0.5 mmol) was added under mechanical stirring, while maintaining the temperature below 94 °C. The mixture was stirred at 90 °C for 3 h, allowed to cool to 70 °C, and the resultant viscous liquid was poured into rapidly stirring ice-cold water (50 mL). The resulting precipitate was collected by filtration and dried in air overnight. The solid was slurried in acetone under reflux for 1 h, the mixture was cooled to room temperature, and the solids were filtered off. The resulting solid was washed with acetone and dried in vacuo to afford benzothiophene.

Synthesis of phenethyl p henyl sulfane (J) [18]. AlCl_3_ (2 mmol) was added at room temperature under an inert atmosphere to a solution of dry THF (5 mL) containing compound **3a** (0.5 mmol). After brief stirring, LiAlH_4_ (0.5 mmol) was introduced in one portion, and the mixture was stirred for 10 min until TLC indicated the complete disappearance of the starting sulfide. The reaction was quenched by slow addition of saturated aqueous Na_2_CO_3_ (5 mL). The aqueous layer was extracted with Et_2_O (3 × 5 mL), the combined organic phases were washed with brine, dried over Na_2_SO_4_, and the solvent was evaporated to afford the sulfide in 84% yield.

Synthesis of *β*-keto sulfone (**K**) [17]. 60% m-CPBA (2 equiv.) was added portion-wise to a 0 °C solution of the sulfide (0.5 mmol) in DCM (2.5 mL). After stirring at room temperature for 6 h, the reaction mixture was monitored by TLC until the reaction was complete. The mixture was then diluted with hexanes, filtered, washed with aqueous Na_2_SO_3_ and water, and dried over MgSO_4_. The residue was purified by column chromatography on silica gel using EA and n-hexane (1:5) as the eluent to provide the *β*-keto sulfone.

1-phenyl-1-trimethylsiloxyethylene (**1a**). White oil. 98% yield, ^1^H NMR (300 MHz, CDCl_3_) δ 7.56–7.49 (m, 2H), 7.28–7.17 (m, 3H), 4.84 (d, *J* = 1.7 Hz, 1H), 4.36 (d, *J* = 1.7 Hz, 1H), 0.20 (s, 9H). ^13^C NMR (75 MHz, CDCl_3_) δ 155.78, 137.63, 128.34, 128.19, 125.33, 91.19, 0.21.

1-(*p*-tolyl)-2-(*p*-tolylthio)ethan-1-one (**3-1a**). 85% yield, yellow solid. (m.p. 55–57 °C). ^1^H NMR (300 MHz, CDCl_3_) δ 7.85–7.76 (m, 2H), 7.30–7.24 (m, 2H), 7.22 (d, *J* = 8.1 Hz, 2H), 7.06 (d, *J* = 8.0 Hz, 2H), 4.16 (s, 2H), 2.38 (s, 3H), 2.28 (s, 3H).^13^C NMR (75 MHz, CDCl_3_) δ 193.81, 144.26, 137.29, 132.86, 131.25, 131.06, 129.82, 129.32, 128.80, 41.70, 21.69, 21.08. HRMS (EI): *m*/*z* calculated for C_16_H_16_OS^+^[M]^+^: 256.0916, found: 256.0914

1-(4-methoxyphenyl)-2-(*p*-tolylthio)ethan-1-one (**3-1b**). 83% yield, yellow solid. (m.p. 58–61 °C). ^1^H NMR (300 MHz, CDCl_3_) δ 7.94–7.88 (m, 2H), 7.30 (d, *J* = 8.2 Hz, 2H), 7.08 (d, *J* = 8.4 Hz, 2H), 6.94–6.89 (m, 2H), 4.17 (s, 2H), 3.85 (s, 3H), 2.30 (s, 3H). ^13^C NMR (75 MHz, CDCl_3_) δ 192.85, 163.70, 137.29, 131.22, 131.16, 131.04, 129.84, 128.36, 113.81, 55.51, 41.56, 21.10. HRMS (EI): *m*/*z* calculated for C_16_H_16_O_2_S^+^[M]^+^: 272.0866, found: 272.0863.

1-(4-(*t*-butyl)phenyl)-2-(*p*-tolylthio)ethan-1-one (**3-1c**). 74% yield, yellow solid. (m.p. 48–50 °C). ^1^H NMR (300 MHz, CDCl_3_) δ 7.95–7.87 (m, 2H), 7.52–7.44 (m, 2H), 7.32 (d, *J* = 8.2 Hz, 2H), 7.10 (d, *J* = 7.7 Hz, 2H), 4.22 (s, 2H), 2.32 (s, 3H), 1.36 (s, 9H). ^13^C NMR (75 MHz, CDCl_3_) δ 193.76, 157.13, 137.29, 132.81, 131.28, 131.11, 129.84, 128.69, 125.61, 41.69, 35.15, 31.08, 21.12. HRMS (EI): *m*/*z* calculated for C_19_H_22_OS^+^[M]^+^: 298.1386, found: 298.1383.

1-(4-fluorophenyl)-2-(*p*-tolylthio)ethanone (**3-1d**). 75% yield, yellow solid. (m.p. 68–72 °C). ^1^H NMR (300 MHz, CDCl_3_) δ 7.99–7.88 (m, 2H), 7.27 (d, *J* = 8.1 Hz, 2H), 7.09 (td, *J* = 8.4, 2.0 Hz, 4H), 4.15 (s, 2H), 2.29 (s, 3H). ^13^C NMR (75 MHz, CDCl_3_) δ 192.69, 165.83 (d, *J* = 255.4 Hz), 137.67, 131.78 (d, *J* = 3.0 Hz), 131.56, 131.45 (d, *J* = 9.4 Hz), 130.61, 129.93, 115.77 (d, *J* = 22.0 Hz), 41.66, 21.11. ^19^F NMR (282 MHz, CDCl_3_) δ −104.33 (tt, *J* = 8.6, 5.4 Hz). HRMS (EI): *m*/*z* calculated for C_15_H_13_FOS^+^[M]^+^: 260.0666, found: 260.0664.

1-(4-chlorophenyl)-2-(*p*-tolylthio)ethan-1-one (**3-1e**). 71% yield, yellow solid. (m.p. 107–109 °C). ^1^H NMR (300 MHz, CDCl_3_) δ 7.90–7.82 (m, 2H), 7.45–7.39 (m, 2H), 7.29–7.25 (m, 2H), 7.09 (d, *J* = 7.7 Hz, 2H), 4.15 (s, 2H), 2.31 (s, 3H). ^13^C NMR (75 MHz, CDCl_3_) δ 193.12, 139.94, 137.94, 133.77, 131.85, 130.48, 130.28, 130.05, 129.07, 41.80, 21.24. HRMS (EI): *m*/*z* calculated for C_15_H_13_ClOS^+^[M]^+^: 276.0370, found: 276.0368.

1-(4-bromophenyl)-2-(*p*-tolylthio)ethan-1-one (**3-1f**). 86% yield, yellow solid. (m.p. 110–114 °C). ^1^H NMR (300 MHz, CDCl_3_) δ 7.81–7.72 (m, 2H), 7.60–7.53 (m, 2H), 7.26 (d, *J* = 8.2 Hz, 2H), 7.08 (d, *J* = 7.9 Hz, 2H), 4.13 (s, 2H), 2.30 (s, 3H). ^13^C NMR (75 MHz, CDCl_3_) δ 193.22, 137.83, 134.09, 131.98, 131.73, 130.41, 130.30, 129.99, 128.63, 41.67, 21.19. HRMS (EI): *m*/*z* calculated for C_15_H_13_BrOS^+^[M]^+^: 319.9865, found: 319.9861.

1-(*o*-tolyl)-2-(*p*-tolylthio)ethan-1-one (**3-1g**). 75% yield, yellow oil. ^1^H NMR (300 MHz, CDCl_3_) δ 7.56 (d, *J* = 7.9 Hz, 1H), 7.37–7.31 (m, 1H), 7.26–7.17 (m, 4H), 7.05 (d, *J* = 8.0 Hz, 2H), 4.15 (s, 2H), 2.39 (s, 3H), 2.28 (s, 3H). ^13^C NMR (75 MHz, CDCl_3_) δ 197.78, 139.13, 137.21, 136.12, 132.06, 131.68, 131.06, 130.99, 129.81, 128.80, 125.58, 44.08, 21.25, 21.09. HRMS (EI): *m*/*z* calculated for C_16_H_16_OS^+^[M]^+^: 256.0916, found: 256.0914.

1-(*m*-tolyl)-2-(*p*-tolylthio)ethan-1-one (**3-1h**). 73% yield, white solid. (m.p. 72–75 °C). ^1^H NMR (300 MHz, CDCl_3_) δ 7.74 (d, *J* = 6.0 Hz, 2H), 7.41–7.29 (m, 4H), 7.13–7.06 (m, 2H), 4.21 (s, 2H), 2.39 (s, 3H), 2.32 (s, 3H). ^13^C NMR (75 MHz, CDCl_3_) δ 194.36, 138.44, 137.41, 135.42, 134.19, 131.44, 131.02, 129.85, 129.20, 128.51, 125.92, 41.88, 21.34, 21.10. HRMS (EI): *m*/*z* calculated for C_16_H_16_OS^+^[M]^+^: 256.0916, found: 256.0915.

(*S*)-1-phenyl-2-(*p*-tolylthio)propan-1-one (**3-1i**). 89% yield. yellow oil. ^1^H NMR (300 MHz, CDCl_3_) δ 8.00–7.90 (m, 2H), 7.57–7.49 (m, 1H), 7.47–7.38 (m, 2H), 7.25–7.19 (m, 2H), 7.10–7.02 (m, 2H), 4.55 (q, *J* = 6.8 Hz, 1H), 2.30 (s, 3H), 1.49 (d, *J* = 6.8 Hz, 3H). ^13^C NMR (75 MHz, CDCl_3_) δ 196.16, 139.05, 135.82, 135.24, 133.03, 129.77, 128.70, 128.60, 127.76, 46.19, 21.27, 16.88. HRMS (ESI): *m*/*z* calculated for C_16_H_16_NaOS^+^[M+Na]^+^: 279.0814, found: 279.0810.

1-phenyl-2-(*p*-tolylthio)hexan-1-one (**3-1j**). 53% yield. yellow oil. ^1^H NMR (300 MHz, CDCl_3_) δ 7.89–7.78 (m, 2H), 7.48–7.40 (m, 1H), 7.37–7.30 (m, 2H), 7.12 (d, *J* = 8.1 Hz, 2H), 6.97 (d, *J* = 8.0 Hz, 2H), 4.28 (t, *J* = 7.2 Hz, 1H), 2.21 (s, 3H), 1.93–1.66 (m, 2H), 1.41–1.20 (m, 4H), 0.79 (t, *J* = 7.1 Hz, 3H). ^13^C NMR (75 MHz, CDCl_3_) δ 195.90, 138.95, 136.44, 135.13, 132.95, 129.75, 128.59, 128.09, 51.57, 30.56, 29.54, 22.62, 21.27, 14.03. HRMS (ESI): *m*/*z* calculated for C_19_H_22_NaOS^+^[M+Na]^+^: 321.1284, found: 321.1293.

1-(naphthalen-2-yl)-2-(*p*-tolylthio)ethan-1-one (**3-1k**). 86% yield, yellow oil. ^1^H NMR (300 MHz, CDCl_3_) δ 8.39 (s, 1H), 8.01 (dd, *J* = 8.7, 1.8 Hz, 1H), 7.91–7.84 (m, 3H), 7.63–7.52 (m, 2H), 7.38–7.31 (m, 2H), 7.10 (d, *J* = 7.7 Hz, 2H), 4.33 (s, 2H), 2.31 (s, 3H). ^13^C NMR (75 MHz, CDCl_3_) δ 194.17, 137.55, 135.61, 132.69, 132.37, 131.61, 130.95, 130.59, 129.90, 129.63, 128.68, 128.50, 127.77, 126.81, 124.22, 41.92, 21.11. HRMS (EI): *m*/*z* calculated for C_19_H_16_OS^+^[M]^+^: 292.096, found: 292.0914.

1-(*p*-tolylthio)butan-2-one (**3-1l**). 35% yield, white solid. (m.p. 30–35 °C). ^1^H NMR (300 MHz, CDCl_3_) δ 7.27–7.23 (m, 2H), 7.10 (d, *J* = 7.8 Hz, 2H), 3.63 (s, 2H), 2.61 (q, *J* = 7.3 Hz, 2H), 2.31 (s, 3H), 1.04 (t, *J* = 7.3 Hz, 3H). ^13^C NMR (75 MHz, CDCl_3_) δ 206.56, 137.33, 131.12, 130.57, 130.06, 44.41, 34.02, 21.19, 8.00. HRMS (EI): *m*/*z* calculated for C_11_H_14_OS^+^[M]^+^: 194.0760, found: 194.0757.

1-phenyl-2-(phenylthio)ethan-1-one (**3-2a**) [19]. 88% yield, yellow oil. ^1^H NMR (300 MHz, CDCl_3_) δ 8.02–7.92 (m, 2H), 7.64–7.56 (m, 1H), 7.52–7.39 (m, 4H), 4.31 (s, 2H). ^13^C NMR (75 MHz, CDCl_3_) δ 194.19, 135.46, 134.85, 133.62, 130.61, 129.19, 128.81, 127.23, 41.33. HRMS (EI): *m*/*z* calculated for C_14_H_12_OS^+^[M]^+^: 228.0603, found: 228.0601.

1-phenyl-2-(*p*-tolylthio)ethanone (**3-2b**) [22]. 91% yield, yellow oil. ^1^H NMR (300 MHz, CDCl_3_) δ 7.98–7.91 (m, 2H), 7.61–7.53 (m, 1H), 7.45 (dd, *J* = 8.3, 6.8 Hz, 2H), 7.34–7.27 (m, 2H), 7.10 (d, *J* = 8.0 Hz, 2H), 4.22 (s, 2H), 2.32 (s, 3H). ^13^C NMR (75 MHz, CDCl_3_) δ 194.17, 137.47, 135.38, 133.41, 131.44, 130.88, 129.88, 128.71, 128.65, 41.80, 21.12. HRMS (EI): *m*/*z* calculated for C_15_H_14_OS^+^[M]^+^: 242.0760, found: 242.0758.

2-((4-methoxyphenyl)thio)-1-phenylethan-1-one (**3-2c**) [23]. 87% yield, yellow oil. 1H NMR (300 MHz, CDCl_3_) δ 7.98–7.91 (m, 2H), 7.61–7.53 (m, 1H), 7.44 (d, *J* = 7.1 Hz, 2H), 7.31 (d, *J* = 8.2 Hz, 2H), 7.10 (d, *J* = 8.0 Hz, 2H), 4.22 (s, 2H), 2.32 (s, 3H). ^13^C NMR (75 MHz, CDCl_3_) δ 194.29, 159.67, 135.39, 134.58, 13 3.32, 128.69, 128.61, 124.51, 114.68, 55.27, 42.76. HRMS (EI): *m*/*z* calculated for C15H14O2S^+^[M]^+^: 258.0709, found: 258.0708.

2-((4-(*t*-butyl)phenyl)thio)-1-phenylethan-1-one (**3-2d**). 86% yield, yellow oil. ^1^H NMR (300 MHz, CDCl_3_) δ 7.98–7.91 (m, 2H), 7.60–7.53 (m, 1H), 7.48–7.41 (m, 2H), 7.38–7.29 (m, 4H), 4.26 (s, 2H), 1.31 (s, 9H). ^13^C NMR (75 MHz, CDCl_3_) δ 194.26, 150.46, 135.44, 133.41, 131.16, 130.82, 128.70, 128.65, 126.15, 41.62, 34.53, 31.26. HRMS (EI): *m*/*z* calculated for C_18_H_20_OS^+^[M]^+^: 284.1229, found: 284.1227.

2-((4-fluorophenyl)thio)-1-phenylethan-1-one (**3-2e**) [24]. 73% yield, yellow oil. ^1^H NMR (300 MHz, CDCl_3_) δ 7.99–7.84 (m, 2H), 7.61–7.51 (m, 1H), 7.49–7.30 (m, 4H), 7.05–6.86 (m, 2H), 4.19 (s, 2H). ^13^C NMR (75 MHz, CDCl_3_) δ 193.96, 162.40 (d, *J* = 247.7 Hz), 135.26, 133.85 (d, *J* = 8.2 Hz), 133.55, 129.46 (d, *J* = 3.4 Hz), 128.70 (d, *J* = 3.4 Hz), 116.22 (d, *J* = 22.0 Hz), 42.07. ^19^F NM R (282 MHz, CDCl_3_) δ −113.62 (tt, *J* = 8.7, 5.2 Hz). HRMS (EI): *m*/*z* calculated for C_14_H_11_FOS^+^[M]^+^: 246.0509, found: 246.0516.

2-((2-chlorophenyl)thio)-1-phenylethanone (**3-2f**) [23]. 69% yield, yellow solid. (m.p. 82–84 °C). ^1^H NMR (300 MHz, CDCl_3_) δ 7.97–7.88 (m, 2H), 7.61–7.53 (m, 1H), 7.45 (t, *J* = 7.5 Hz, 2H), 7.32–7.26 (m, 2H), 7.25–7.18 (m, 2H), 4.24 (s, 2H). ^13^C NMR (75 MHz, CDCl3) δ 193.74, 135.18, 133.65, 133.24, 133.18, 131.84, 129.20, 128.77, 128.67, 41.19. HRMS (EI): *m*/*z* calculated for C_14_H_11_ClOS^+^[M]^+^: 262.0214, found: 262.0211.

2-((4-bromophenyl)thio)-1-phenylethan-1-one (**3-2g**) [24]. 65% yield, white solid. (m.p. 85–88 °C). ^1^H NMR (300 MHz, CDCl_3_) δ 7.96–7.87 (m, 2H), 7.58 (t, *J* = 7.4 Hz, 1H), 7.49–7.35 (m, 4H), 7.22 (d, *J* = 8.5 Hz, 2H), 4.25 (s, 2H). ^13^C NMR (75 MHz, CDCl_3_) δ 193.74, 135.19, 133.98, 133.71, 132.15, 131.95, 128.81, 128.71, 121.16, 41.04. HRMS (EI): *m*/*z* calculated for C_14_H_11_BrOS^+^[M]^+^: 305.9708, found: 305.9707.

1-phenyl-2-(*o*-tolylthio)ethan-1-one (**3-2h**). 82% yield, yellow solid. (m.p. 57–60 °C). ^1^H NMR (300 MHz, CDCl_3_) δ 8.00–7.92 (m, 2H), 7.62–7.54 (m, 1H), 7.46 (t, *J* = 7.6 Hz, 2H), 7.39–7.32 (m, 1H), 7.21–7.10 (m, 3H), 4.25 (s, 2H), 2.39 (s, 3H). ^13^C NMR (75 MHz, CDCl_3_) δ 194.02, 138.62, 135.32, 133.94, 133.43, 130.27, 130.14, 128.63, 128.62, 126.99, 126.62, 40.44, 20.43. HRMS (EI): *m*/*z* calculated for C_15_H_14_OS^+^[M]^+^: 242.0760, found: 242.0758.

1-phenyl-2-(*m*-tolylthio)ethan-1-one (**3-2i**). 73% yield, yellow oil. ^1^H NMR (300 MHz, CDCl_3_) δ 7.99–7.91 (m, 2H), 7.61–7.54 (m, 1H), 7.46 (t, *J* = 7.5 Hz, 2H), 7.24–7.13 (m, 3H), 7.04 (d, *J* = 6.8 Hz, 1H), 4.28 (s, 2H), 2.31 (s, 3H). ^13^C NMR (75 MHz, CDCl_3_) δ 194.10, 138.82, 135.36, 134.50, 133.45, 131.01, 128.91, 128.66, 127.93, 127.34, 41.17, 21.30. HRMS (EI): *m*/*z* calculated for C_15_H_14_OS^+^[M]^+^: 242.0760, found: 242.0757.

2-((2-isopropylphenyl)thio)-1-phenylethan-1-one (**3-2j**). 75% yield, yellow oil. ^1^H NMR (300 MHz, CDCl_3_) δ 7.92 (d, *J* = 7.0 Hz, 2H), 7.55 (t, *J* = 7.4 Hz, 1H), 7.47–7.36 (m, 3H), 7.27–7.19 (m, 2H), 7.16–7.07 (m, 1H), 4.22 (s, 2H), 3.47 (p, *J* = 6.9 Hz, 1H), 1.16 (d, *J* = 6.9 Hz, 6H). ^13^C NMR (75 MHz, CDCl_3_) δ 194.20, 149.66, 135.47, 133.49, 132.87, 131.51, 128.75, 128.69, 127.82, 126.55, 125.80, 41.75, 30.42, 23.59. HRMS (EI): *m*/*z* calculated for C_17_H_18_OS^+^[M]^+^: 270.1073, found: 270.1072.

2-((2-chlorophenyl)thio)-1-phenylethan-1-one (**3-2k**) [25]. 67% yield, yellow solid. (m.p. 84–87 °C). ^1^H NMR (300 MHz, CDCl_3_) δ 8.00–7.92 (m, 2H), 7.61–7.56 (m, 1H), 7.46 (t, *J* = 7.5 Hz, 2H), 7.40–7.36 (m, 2H), 7.22–7.12 (m, 2H), 4.33 (s, 2H). ^13^C NMR (75 MHz, CDCl_3_) δ 193.79, 135.36, 134.75, 133.99, 133.74, 130.81, 129.92, 128.83, 128.75, 127.99, 127.41, 39.79. HRMS (EI): *m*/*z* calculated for C_14_H_11_ClOS^+^[M]^+^: 262.0214, found: 262.0210.

2-((3,4-dimethoxyphenyl)thio)-1-phenylethanone (**3-2l**). 85% yield, yellow solid. ^1^H NMR (300 MHz, CDCl_3_) δ 7.85–7.77 (m, 2H), 7.50–7.z42 (m, 1H), 7.34 (t, *J* = 7.5 Hz, 2H), 6.90 (dd, *J* = 8.3, 2.1 Hz, 1H), 6.83 (d, *J* = 2.1 Hz, 1H), 6.67 (d, *J* = 8.3 Hz, 1H), 4.06 (s, 2H), 3.74 (s, 3H), 3.70 (s, 3H). ^13^C NMR (75 MHz, CDCl_3_) δ 194.39, 149.14, 148.89, 135.38, 133.33, 128.67, 128.58, 125.63, 124.87, 115.85, 111.46, 55.83, 42.59. HRMS (EI): *m*/*z* calculated for C_16_H_16_O3S^+^[M]^+^: 288.0815, found: 288.0812.

2-(naphthalen-2-ylthio)-1-phenylethan-1-one (**3-2m**) [23]. 77% yield, yellow solid. (m.p. 50–53 °C). ^1^H NMR (300 MHz, CDCl_3_) δ 7.99–7.95 (m, 2H), 7.84–7.72 (m, 4H), 7.61–7.56 (m, 1H), 7.50–7.43 (m, 5H), 4.38 (s, 2H). ^13^C NMR (75 MHz, CDCl_3_) δ 194.14, 135.48, 133.76, 133.66, 132.29, 132.27, 128.92, 128.83, 128.79, 128.05, 127.82, 127.45, 126.72, 126.25, 41.23. HRMS (EI): *m*/*z* calculated for C_18_H_14_OS^+^[M]^+^: 278.0760, found: 278.0758.

1-phenyl-2-(thiophen-2-ylthio)ethanone (**3-2n**). 45% yield, purple oil. ^1^H NMR (300 MHz, CDCl_3_) δ 7.94–7.87 (m, 2H), 7.61–7.55 (m, 1H), 7.49–7.43 (m, 2H), 7.37 (dd, *J* = 5.4, 1.3 Hz, 1H), 7.12 (dd, *J* = 3.6, 1.3 Hz, 1H), 6.95 (dd, *J* = 5.4, 3.6 Hz, 1H), 4.17 (s, 2H). ^13^C NMR (75 MHz, CDCl_3_) δ 194.00, 135.46, 135.44, 133.59, 132.21, 130.73, 128.78, 128.75, 127.80, 45.36. HRMS (EI): *m*/*z* calculated for C_12_H_10_OS_2_^+^[M]^+^: 234.0168, found: 234.0166.

2-((1s, 3s)-adamantan-1-ylthio)-1-phenylethanone (**3-2o**). 30% yield, yellow oil. ^1^H NMR (300 MHz, CDCl_3_) δ 7.92–7.86 (m, 2H), 7.52–7.45 (m, 1H), 7.39 (dd, *J* = 8.3, 6.7 Hz, 2H), 3.79 (s, 2H), 2.00–1.94 (m, 3H), 1.82 (d, *J* = 3.0 Hz, 6H), 1.60 (s, 6H). ^13^C NMR (75 MHz, CDCl_3_) δ 196.68, 135.69, 133.35, 128.89, 128.68, 46.00, 43.30, 36.22, 33.18, 29.79. HRMS (ESI): *m*/*z* calculated for C_18_H_22_NaOS^+^[M+Na]^+^: 309.1284, found: 309.129.

butyl 2-((2-oxo-2-phenylethyl)thio)acetate (**3-2p**). 35% yield, yellow oil. ^1^H NMR (300 MHz, CDCl_3_) δ 7.99–7.93 (m, 2H), 7.61–7.54 (m, 1H), 7.49–7.43 (m, 2H), 4.11 (t, *J* = 6.7 Hz, 2H), 4.03 (s, 2H), 3.32 (s, 2H), 1.64–1.55 (m, 2H), 1.40–1.30 (m, 2H), 0.91 (t, *J* = 7.3 Hz, 3H). ^13^C NMR (75 MHz, CDCl_3_) δ 194.10, 170.04, 135.40, 133.64, 128.82, 128.70, 65.48, 37.78, 33.44, 30.60, 19.14, 13.77. HRMS (EI): *m*/*z* calculated for C_14_H_18_O_3_S^+^[M]^+^: 266.0971, found: 266.0969.

2,2′-(1,2-phenylenebis(sulfanediyl))bis(1-phenylethan-1-one) (**3-2q**). 36% yield, yellow oil. ^1^H NMR (300 MHz, CDCl_3_) δ 7.95–7.87 (m, 4H), 7.55 (t, *J* = 7.4 Hz, 2H), 7.45–7.33 (m, 6H), 7.12–7.15 (m, 2H), 4.28 (s, 4H). ^13^C NMR (75 MHz, CDCl_3_) δ 194.07, 136.18, 135.40, 133.56, 131.14, 128.73, 128.70, 127.79, 40.27. HRMS (ESI): *m*/*z* calculated for C_22_H_18_NaO_2_S_2_^+^[M+Na]^+^: 401.0640, found: 401.0631.

1-(4-methoxyphenyl)-2-((3-methoxyphenyl)thio)ethan-1-one (**D**). 82% yield, yellow oil. ^1^H NMR (300 MHz, CDCl_3_) δ 8.00–7.87 (m, 2H), 7.18 (t, *J* = 7.9 Hz, 1H), 6.91 (d, *J* = 9.1 Hz, 4H), 6.73 (dd, *J* = 8.3, 1.6 Hz, 1H), 4.25 (s, 2H), 3.85 (s, 3H), 3.75 (s, 3H). ^13^C NMR (75 MHz, CDCl_3_) δ 192.73, 163.83, 159.82, 136.49, 131.06, 129.88, 128.34, 121.97, 115.11, 113.89, 112.67, 55.55, 55.28, 40.74. HRMS (EI): *m*/*z* calculated for C_16_H_16_O_3_S^+^[M]^+^: 288.0815, found: 288.0812.

6-methoxy-2-(4-methoxyphenyl)benzo[*b*]thiophene (**E**). 87% yield, white solid. (m.p. 163–165 °C). ^1^H NMR (300 MHz, CDCl_3_) δ 7.66–7.54 (m, 3H), 7.37–7.26 (m, 2H), 7.01–6.89 (m, 3H), 3.88 (s, 3H), 3.85 (s, 3H). ^13^C NMR (75 MHz, CDCl_3_) δ 159.59, 157.30, 141.64, 140.74, 135.03, 127.55, 127.40, 124.03, 117.89, 114.49, 114.44, 105.00, 55.76, 55.53. HRMS (EI): *m*/*z* calculated for C_15_H_14_O_2_S^+^[M]^+^: 270.0709, found: 270.0707.

2-((2-hydroxyphenyl)thio)-1-phenylpropan-1-one (**F**). 75% yield, yellow oil. ^1^H NMR (300 MHz, CDCl_3_) δ 7.98–7.82 (m, 2H), 7.60–7.51 (m, 1H), 7.43 (t, *J* = 7.5 Hz, 2H), 7.35–7.22 (m, 2H), 7.05 (s, 1H), 6.94 (dd, *J* = 8.2, 1.4 Hz, 1H), 6.81 (td, *J* = 7.5, 1.3 Hz, 1H), 4.62 (q, *J* = 7.0 Hz, 1H), 1.48 (d, *J* = 7.0 Hz, 3H). ^13^C NMR (75 MHz, CDCl_3_) δ 197.33, 158.22, 137.71, 135.04, 133.62, 132.31, 128.82, 128.72, 120.67, 115.47, 115.32, 47.24, 17.32. HRMS (EI): *m*/*z* calculated for C_15_H_14_OS^+^[M]^+^: 258.0709, found: 258.0706.

3-methyl-2-phenyl-2,3-dihydrobenzo[*b*][1,4]oxathiine (**G**). 88% yield, yellow oil. ^1^H NMR (300 MHz, CDCl_3_) δ 7.50–7.32 (m, 5H), 7.16–6.90 (m, 4H), 5.50 (s, 1H), 3.43 (q, *J* = 7.1 Hz, 1H), 1.26 (d, *J* = 6.9 Hz, 3H). ^13^C NMR (75 MHz, CDCl_3_) δ 151.49, 139.38, 128.49, 127.92, 127.85, 125.91, 125.64, 121.99, 118.53, 116.90, 76.74, 38.26, 15.20. HRMS (EI): *m*/*z* calculated for C_15_H_14_OS^+^[M]^+^: 242.0760, found: 242.0756.

phenyl-2-(phenylthio)ethan-1-ol (**H**) [19]. 88% yield, yellow oil. ^1^H NMR (300 MHz, CDCl_3_) δ 7.42–7.19 (m, 10H), 4.69 (dd, *J* = 9.4, 3.6 Hz, 1H), 3.29 (dd, *J* = 13.8, 3.6 Hz, 1H), 3.07 (dd, *J* = 13.8, 9.4 Hz, 1H). ^13^C NMR (75 MHz, CDCl3) δ 142.22, 134.98, 130.21, 129.21, 128.63, 128.06, 126.82, 125.94, 71.72, 43.96. HRMS (ESI): *m*/*z* calculated for C_14_H_14_NaOS^+^ [M+Na]^+^: 253.0658, found: 253.0658.

2-phenylbenzo[*b*]thiophene (**I**). 83% yield, white solid. (m.p. 166–170 °C). ^1^H NMR (300 MHz, CDCl_3_) δ 7.90–7.67 (m, 4H), 7.56 (s, 1H), 7.48–7.29 (m, 5H). ^13^C NMR (75 MHz, CDCl_3_) δ 144.38, 140.83, 139.63, 134.43, 129.09, 128.41, 126.64, 124.65, 124.46, 123.70, 122.41, 119.59. HRMS (EI): *m*/*z* calculated for C_14_H_10_S^+^[M]^+^: 210.0498, found: 210.0496.

phenethyl(phenyl)sulfane (**J**). 84% yield, white oil. ^1^H NMR (300 MHz, CDCl_3_) δ 7.41–7.09 (m, 10H), 3.15 (t, *J* = 7.8 Hz, 2H), 2.90 (t, *J* = 7.9 Hz, 2H). ^13^C NMR (75 MHz, CDCl_3_) δ 140.28, 136.45, 129.22, 129.02, 128.60, 126.54, 126.04, 35.69, 35.12. HRMS (EI): *m*/*z* calculated for C_14_H_14_S^+^[M]^+^: 214.0811, found: 214.0812.

1-phenyl-2-(phenylsulfonyl)ethan-1-one (**K**). 75% yield, yellow solid. (m.p. 91–95 °C). ^1^H NMR (300 MHz, CDCl_3_) δ 7.90–7.86 (m, 4H), 7.59–7.35 (m, 6H), 4.75 (s, 2H). ^13^C NMR (75 MHz, CDCl_3_) δ 188.01, 138.68, 135.54, 134.24, 134.11, 129.10, 128.72, 128.36, 63.10. HRMS (ESI): *m*/*z* calculated for C_14_H_12_NaO_3_S^+^[M+Na]^+^: 283.0399, found: 283.0400.

## 4. Conclusions

In conclusion, a simple and efficient synthetic approach has been developed, enabling the direct synthesis of various *β*-keto sulfides without the use of any catalysts under air atmosphere. In this context, enol silyl ethers can be readily and efficiently synthesized from ketones. Importantly, this method offers notable advantages in terms of practicality, including inexpensive and readily available starting materials, mild reaction conditions, ease of product purification, and high chemoselectivity. Furthermore, a plausible reaction mechanism has been proposed.

## Figures and Tables

**Table 1:** Optimization of the reaction conditions *^a^*.

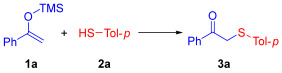					
Entry	2a (eq.)	Solvent	Atmosphere	Time (h)	Yield (%) *^b^*
1	2	Toluene	air	4	32
2	2	MeCN	air	4	20
3	2	DMSO	air	4	35
4	2	THF	air	4	38
5	2	DMF	air	4	34
6	2	DCM	air	4	56
7	2	1,4-Dioxane	air	4	64
8	2	H_2_O	air	4	28
9	2	EtOH	air	4	78
10	2	MeOH	air	4	95
11	1	MeOH	air	4	64
12	1.5	MeOH	air	4	93
13	2	MeOH	air	1	95
14	2	MeOH	N_2_	1	17

*^a^ *Reaction conditions: **1a** (0.2 mmol), solvent (2 mL), r.t.; *^b^ * GC-MS yield.

## Data Availability

The original contributions presented in this study are included in the article/supplementary material. Further inquiries can be directed to the corresponding authors.

## Supplementary Materials

The following supporting information can be downloaded at: https://www.mdpi.com/article/10.3390/molecules30194032/s1, Radical inhibition experiment.
