# Supplementary material for: Thiofunctionalization of Silyl Enol Ether: An Efficient Approach for the Synthesis of β-Keto Sulfides
Source: Molecules. 2025 Oct 9;30(19):4032. doi: 10.3390/molecules30194032 (PMC12526227; doi:10.3390/molecules30194032)
Supplement: Supplementary file 1 [file molecules-30-04032-s001.zip › molecules-3884736-supplementary.pdf]

Supporting Information

Thiofunctionalization of Silyl Enol Ether: An Efficient Approach for the Synthesis of *β*-Keto Sulfides

Xinyao Zhao, Hexia Ye, Yajie Fu, Haibo Liu*, Xiaojing Bi*,

State Key Laboratory of Chemistry for NBC Hazards Protection, Beijing 102205, China;

xy01253@yeah.net (X.Z.); yehexia6688@yeah.net (H.Y.); yajief2022@163.com (Y.F.)

*Correspondence:hbbnu@126.com (H.L.); xiaojingbimail@yeah.net (X.B.)

Radical inhibition experiment

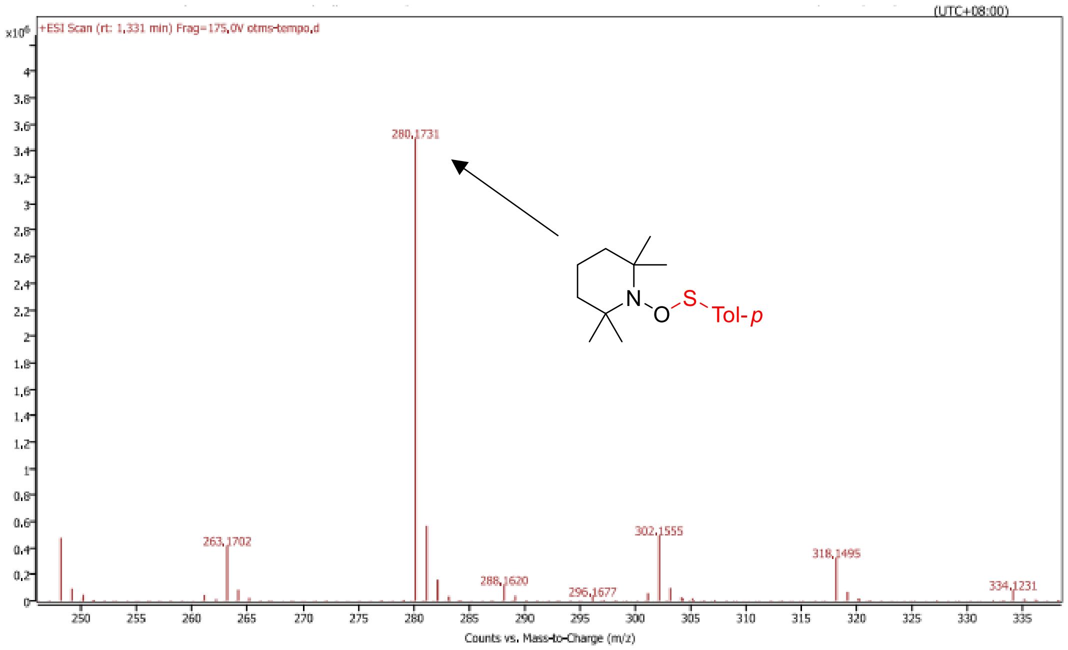

HRMS (ESI): m/z calculated for $C_{16}H_{26}NOS^{+}[M+H]^{+}$: 280.1730, found: 280.1731.

Characterization Data

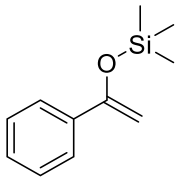

1-Phenyl-1-trimethylsiloxyethylene (1a)

98% yield, White oil. ^{1}H NMR (300 MHz, $CDCl_3$) δ 7.56 – 7.49 (m, 2H), 7.28 – 7.17 (m, 3H), 4.84 (d, J = 1.7 Hz, 1H), 4.36 (d, *J* = 1.7 Hz, 1H), 0.20 (s, 9H). ^{13}C NMR (75 MHz, $CDCl_3$) δ 155.78, 137.63, 128.34, 128.19, 125.33, 91.19, 0.21.

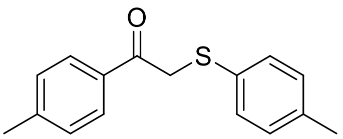

1-(*p*-tolyl)-2-(*p*-tolylthio)ethan-1-one (3-1a)

85% yield, yellow solid. (m.p. 55-57°C). ^{1}H NMR (300 MHz, $CDCl_3$) δ 7.85 – 7.76 (m, 2H), 7.30 – 7.24 (m, 2H), 7.22 (d, J = 8.1 Hz, 2H), 7.06 (d, J = 8.0 Hz, 2H), 4.16 (s, 2H), 2.38 (s, 3H), 2.28 (s, 3H). ^{13}C NMR (75 MHz, $CDCl_3$) δ 193.81, 144.26, 137.29, 132.86, 131.25, 131.06, 129.82, 129.32, 128.80, 41.70, 21.69, 21.08.

HRMS (EI): m/z calculated for $C_{16}H_{16}OS^{+}[M]^{+}$: 256.0916, found: 256.0914.

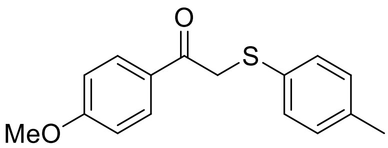

1-(4-methoxyphenyl)-2-(*p*-tolylthio)ethan-1-one (3-1b)

83% yield, yellow solid. (m.p. 58-61°C). ^{1}H NMR (300 MHz, $CDCl_3$) δ 7.94 – 7.88 (m, 2H), 7.30 (d, J = 8.2 Hz, 2H), 7.08 (d, J = 8.4 Hz, 2H), 6.94 – 6.89 (m, 2H), 4.17 (s, 2H), 3.85 (s, 3H), 2.30 (s, 3H). ^{13}C NMR (75 MHz, $CDCl_3$) δ 192.85, 163.70, 137.29, 131.22, 131.16, 131.04, 129.84, 128.36, 113.81, 55.51, 41.56, 21.10.

HRMS (EI): m/z calculated for $C_{16}H_{16}O_2S^+[M]^+$: 272.0866, found: 272.0863.

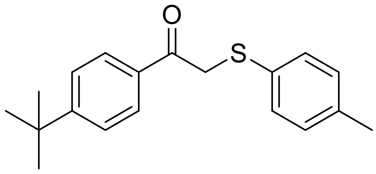

1-(4-(tert-butyl)phenyl)-2-(*p*-tolylthio)ethan-1-one (3-1c)

74% yield, yellow solid. (m.p. 48-50°C). ^{1}H NMR (300 MHz, $CDCl_3$) δ 7.95 – 7.87 (m, 2H), 7.52 – 7.44 (m, 2H), 7.32 (d, J = 8.2 Hz, 2H), 7.10 (d, J = 7.7 Hz, 2H), 4.22 (s, 2H), 2.32 (s, 3H), 1.36 (s, 9H). ^{13}C NMR (75 MHz, $CDCl_3$) δ 193.76, 157.13, 137.29, 132.81, 131.28, 131.11, 129.84, 128.69, 125.61, 41.69, 35.15, 31.08, 21.12.

HRMS (EI): m/z calculated for $C_{19}H_{22}OS^+[M]^+$: 298.1386, found: 298.1383.

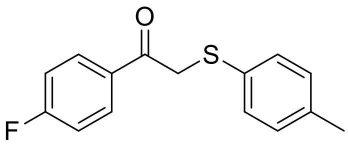

1-(4-fluorophenyl)-2-(*p*-tolylthio)ethanone (3-1d)

75% yield, yellow solid. (m.p. 68-72°C). ^{1}H NMR (300 MHz, $CDCl_3$) δ 7.99 – 7.88 (m, 2H), 7.27 (d, J = 8.1 Hz, 2H), 7.09 (td, J = 8.4, 2.0 Hz, 4H), 4.15 (s, 2H), 2.29 (s, 3H). ^{13}C NMR (75 MHz, $CDCl_3$) δ 192.69, 165.83 (d, J = 255.4 Hz), 137.67, 131.78 (d, J = 3.0 Hz), 131.56, 131.45 (d, J = 9.4 Hz), 130.61, 129.93, 115.77 (d, J = 22.0 Hz), 41.66, 21.11. ^{19}F NMR (282 MHz, $CDCl_3$) δ −104.33 (tt, J = 8.6, 5.4 Hz).

HRMS (EI): m/z calculated for $C_{15}H_{13}FOS^+[M]^+$: 260.0666, found: 260.0664.

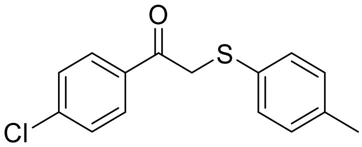

1-(4-chlorophenyl)-2-(*p*-tolylthio)ethan-1-one (3-1e)

71% yield, yellow solid. (m.p. 107-109°C). ^{1}H NMR (300 MHz, $CDCl_3$) δ 7.90 – 7.82 (m, 2H), 7.45 – 7.39 (m, 2H), 7.29 – 7.25 (m, 2H), 7.09 (d, J = 7.7 Hz, 2H), 4.15 (s, 2H), 2.31 (s, 3H). ^{13}C NMR (75 MHz, $CDCl_3$) δ 193.12, 139.94, 137.94, 133.77, 131.85, 130.48, 130.28, 130.05, 129.07, 41.80, 21.24.

HRMS (EI): m/z calculated for $C_{15}H_{13}ClOS^+[M]^+$: 276.0370, found: 276.0368.

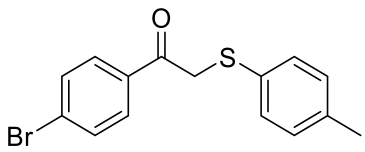

1-(4-bromophenyl)-2-(*p*-tolylthio)ethan-1-one (3-1f)

86% yield, yellow solid. (m.p. 110-114°C). ^{1}H NMR (300 MHz, $CDCl_3$) δ 7.81 – 7.72 (m, 2H), 7.60 – 7.53 (m, 2H), 7.26 (d, *J* = 8.2 Hz, 2H), 7.08 (d, *J* = 7.9 Hz, 2H), 4.13 (s, 2H), 2.30 (s, 3H). ^{13}C NMR (75 MHz, $CDCl_3$) δ 193.22, 137.83, 134.09, 131.98, 131.73, 130.41, 130.30, 129.99, 128.63, 41.67, 21.19.

HRMS (EI): m/z calculated for $C_{15}H_{13}BrOS^+[M]^+$: 319.9865, found:319.9861.

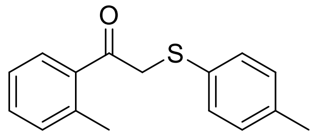

1-(*o*-tolyl)-2-(*p*-tolylthio)ethan-1-one (3-1g)

75% yield, yellow oil. ^{1}H NMR (300 MHz, $CDCl_3$) δ 7.56 (d, *J* = 7.9 Hz, 1H), 7.37 – 7.31 (m, 1H), 7.26 – 7.17 (m, 4H), 7.05 (d, *J* = 8.0 Hz, 2H), 4.15 (s, 2H), 2.39 (s, 3H), 2.28 (s, 3H). ^{13}C NMR (75 MHz, $CDCl_3$) δ 197.78, 139.13, 137.21, 136.12, 132.06, 131.68, 131.06, 130.99, 129.81, 128.80, 125.58, 44.08, 21.25, 21.09.

HRMS (EI): m/z calculated for $C_{16}H_{16}OS^+[M]^+$: 256.0916, found: 256.0914.

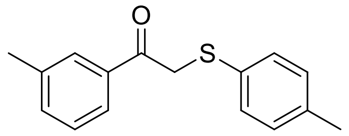

1-(*m*-tolyl)-2-(*p*-tolylthio)ethan-1-one (3-1h)

73% yield, white solid. (m.p. 72-75°C). ^{1}H NMR (300 MHz, $CDCl_3$) δ 7.74 (d, *J* = 6.0 Hz, 2H), 7.41 – 7.29 (m, 4H), 7.13 – 7.06 (m, 2H), 4.21 (s, 2H), 2.39 (s, 3H), 2.32 (s, 3H). ^{13}C NMR (75 MHz, $CDCl_3$) δ 194.36, 138.44, 137.41, 135.42, 134.19, 131.44, 131.02, 129.85, 129.20, 128.51, 125.92, 41.88, 21.34, 21.10.

HRMS (EI): m/z calculated for $C_{16}H_{16}OS^+[M]^+$: 256.0916, found: 256.0915.

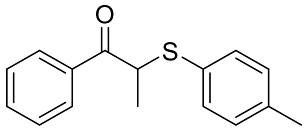

(S)-1-phenyl-2-(*p*-tolylthio)propan-1-one (3-1i)

89% yield. yellow oil. ^{1}H NMR (300 MHz, $CDCl_3$) δ 8.00 – 7.90 (m, 2H), 7.57 – 7.49 (m, 1H), 7.47 – 7.38 (m, 2H), 7.25 – 7.19 (m, 2H), 7.10 – 7.02 (m, 2H), 4.55 (q, *J* = 6.8 Hz, 1H), 2.30 (s, 3H), 1.49 (d, *J* = 6.8 Hz, 3H). ^{13}C NMR (75 MHz, $CDCl_3$) δ 196.16, 139.05, 135.82, 135.24, 133.03, 129.77, 128.70, 128.60, 127.76, 46.19, 21.27, 16.88.

HRMS (ESI): m/z calculated for $C_{16}H_{16}NaOS^+[M+Na]^+$: 279.0814, found: 279.0810.

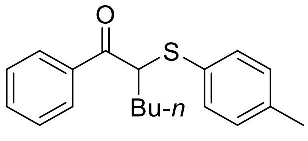

1-phenyl-2-(*p*-tolylthio)hexan-1-one (3-1j)

53% yield. yellow oil. ^{1}H NMR (300 MHz, $CDCl_3$) δ 7.89 – 7.78 (m, 2H), 7.48 – 7.40 (m, 1H), 7.37 – 7.30 (m, 2H), 7.12 (d, *J* = 8.1 Hz, 2H), 6.97 (d, *J* = 8.0 Hz, 2H), 4.28 (t, *J* = 7.2 Hz, 1H), 2.21 (s, 3H), 1.93 – 1.66 (m, 2H), 1.41 – 1.20 (m, 4H), 0.79 (t, *J* = 7.1 Hz, 3H). ^{13}C NMR (75 MHz, $CDCl_3$) δ 195.90, 138.95, 136.44, 135.13, 132.95, 129.75, 128.59, 128.09, 51.57, 30.56, 29.54, 22.62, 21.27, 14.03.

HRMS (ESI): m/z calculated for $C_{19}H_{22}NaOS^+[M+Na]^+$: 321.1284, found: 321.1293.

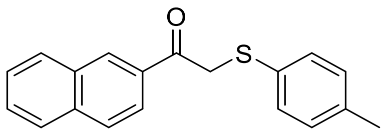

1-(naphthalen-2-yl)-2-(*p*-tolylthio)ethan-1-one (3-1k)

86% yield, yellow oil. ^{1}H NMR (300 MHz, $CDCl_3$) δ 8.39 (s, 1H), 8.01 (dd, *J* = 8.7, 1.8 Hz, 1H), 7.91 – 7.84 (m, 3H), 7.63 – 7.52 (m, 2H), 7.38 – 7.31 (m, 2H), 7.10 (d, *J* = 7.7 Hz, 2H), 4.33 (s, 2H), 2.31 (s, 3H). ^{13}C NMR (75 MHz, $CDCl_3$) δ 194.17, 137.55, 135.61, 132.69, 132.37, 131.61, 130.95, 130.59, 129.90, 129.63, 128.68, 128.50, 127.77, 126.81, 124.22, 41.92, 21.11.

HRMS (EI): m/z calculated for $C_{19}H_{16}OS^+[M]^+$: 292.096, found: 292.0914.

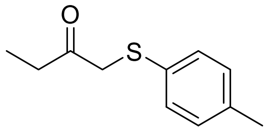

1-(*p*-tolylthio)butan-2-one (3-1l)

35% yield, white solid. (m.p. 30-35°C). ^{1}H NMR (300 MHz, $CDCl_3$) δ 7.27 – 7.23 (m, 2H), 7.10 (d, *J* = 7.8 Hz, 2H), 3.63 (s, 2H), 2.61 (q, *J* = 7.3 Hz, 2H), 2.31 (s, 3H), 1.04 (t, *J* = 7.3 Hz, 3H). ^{13}C NMR (75 MHz, $CDCl_3$) δ 206.56, 137.33, 131.12, 130.57, 130.06, 44.41, 34.02, 21.19, 8.00.

HRMS (EI): m/z calculated for $C_{11}H_{14}OS^+[M]^+$: 194.0760, found: 194.0757.

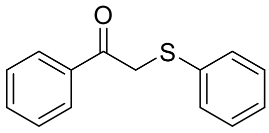

1-phenyl-2-(phenylthio)ethan-1-one (3-2a) [1]

88% yield, yellow oil. ^{1}H NMR (300 MHz, $CDCl_3$) δ 8.02 – 7.92 (m, 2H), 7.64 – 7.56 (m, 1H), 7.52 – 7.39 (m, 4H), 4.31 (s, 2H). ^{13}C NMR (75 MHz, $CDCl_3$) δ 194.19, 135.46, 134.85, 133.62, 130.61, 129.19, 128.81, 127.23, 41.33.

HRMS (EI): m/z calculated for $C_{14}H_{12}OS^+[M]^+$: 228.0603, found: 228.0601.

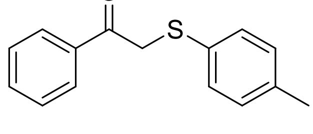

1-phenyl-2-(*p*-tolylthio)ethanone (3-2b) [2]

91% yield, yellow oil. ^{1}H NMR (300 MHz, $CDCl_3$) δ 7.98 – 7.91 (m, 2H), 7.61 – 7.53 (m, 1H), 7.45 (dd, *J* = 8.3, 6.8 Hz, 2H), 7.34 – 7.27 (m, 2H), 7.10 (d, *J* = 8.0 Hz, 2H), 4.22 (s, 2H), 2.32 (s, 3H). ^{13}C NMR (75 MHz, $CDCl_3$) δ 194.17, 137.47, 135.38, 133.41, 131.44, 130.88, 129.88,

128.71, 128.65, 41.80, 21.12.
HRMS (EI): m/z calculated for $C_{15}H_{14}OS^{+}[M]^{+}$: 242.0760, found: 242.0758.

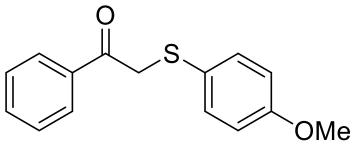

2-((4-methoxyphenyl)thio)-1-phenylethan-1-one (3-2c) [3]

87% yield, yellow oil. ^{1}H NMR (300 MHz, $CDCl_3$) δ 7.98 – 7.91 (m, 2H), 7.61 – 7.53 (m, 1H), 7.44 (d, *J* = 7.1 Hz, 2H), 7.31 (d, *J* = 8.2 Hz, 2H), 7.10 (d, *J* = 8.0 Hz, 2H), 4.22 (s, 2H), 2.32 (s, 3H). ^{13}C NMR (75 MHz, $CDCl_3$) δ 194.29, 159.67, 135.39, 134.58, 133.32, 128.69, 128.61, 124.51, 114.68, 55.27, 42.76.
HRMS (EI): m/z calculated for $C_{15}H_{14}O_2S^{+}[M]^{+}$: 258.0709, found: 258.0708.

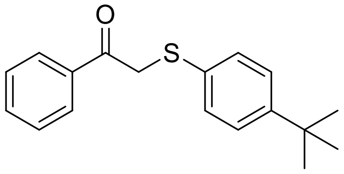

2-((4-(*t*-butyl)phenyl)thio)-1-phenylethan-1-one (3-2d)

86% yield, yellow oil. ^{1}H NMR (300 MHz, $CDCl_3$) δ 7.98 – 7.91 (m, 2H), 7.60 – 7.53 (m, 1H), 7.48 – 7.41 (m, 2H), 7.38 – 7.29 (m, 4H), 4.26 (s, 2H), 1.31 (s, 9H). ^{13}C NMR (75 MHz, $CDCl_3$) δ 194.26, 150.46, 135.44, 133.41, 131.16, 130.82, 128.70, 128.65, 126.15, 41.62, 34.53, 31.26.
HRMS (EI): m/z calculated for $C_{18}H_{20}OS^{+}[M]^{+}$: 284.1229, found: 284.1227.

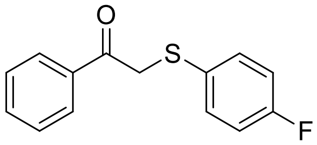

2-((4-fluorophenyl)thio)-1-phenylethan-1-one (3-2e) [4]

73% yield, yellow oil. ^{1}H NMR (300 MHz, $CDCl_3$) δ 7.99 – 7.84 (m, 2H), 7.61 – 7.51 (m, 1H), 7.49 – 7.30 (m, 4H), 7.05 – 6.86 (m, 2H), 4.19 (s, 2H). ^{13}C NMR (75 MHz, $CDCl_3$) δ 193.96, 162.40 (d, *J* = 247.7 Hz), 135.26, 133.85 (d, *J* = 8.2 Hz), 133.55, 129.46 (d, *J* = 3.4 Hz), 128.70 (d, *J* = 3.4 Hz), 116.22 (d, *J* = 22.0 Hz), 42.07. ^{19}F NMR (282 MHz, $CDCl_3$) δ –113.62 (tt, *J* = 8.7, 5.2 Hz).
HRMS (EI): m/z calculated for $C_{14}H_{11}FOS^{+}[M]^{+}$: 246.0509, found: 246.0516.

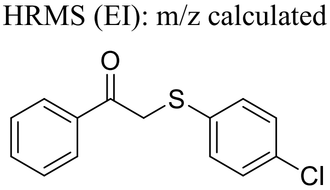

2-((2-chlorophenyl)thio)-1-phenylethanone (3-2f) [3]

69% yield, yellow solid. (m.p. 82-84°C). ^{1}H NMR (300 MHz, $CDCl_3$) δ 7.97 – 7.88 (m, 2H), 7.61 – 7.53 (m, 1H), 7.45 (t, *J* = 7.5 Hz, 2H), 7.32 – 7.26 (m, 2H), 7.25 – 7.18 (m, 2H), 4.24 (s, 2H). ^{13}C NMR (75 MHz, $CDCl_3$) δ 193.74, 135.18, 133.65, 133.24, 133.18, 131.84, 129.20, 128.77, 128.67, 41.19.
HRMS (EI): m/z calculated for $C_{14}H_{11}ClOS^{+}[M]^{+}$: 262.0214, found: 262.0211.

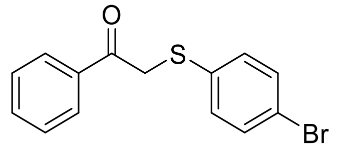

2-((4-bromophenyl)thio)-1-phenylethan-1-one (3-2g) [4]

65% yield, white solid. (m.p. 85-88°C). ^{1}H NMR (300 MHz, $CDCl_3$) δ 7.96 – 7.87 (m, 2H), 7.58 (t, J = 7.4 Hz, 1H), 7.49 – 7.35 (m, 4H), 7.22 (d, J = 8.5 Hz, 2H), 4.25 (s, 2H). ^{13}C NMR (75 MHz, $CDCl_3$) δ 193.74, 135.19, 133.98, 133.71, 132.15, 131.95, 128.81, 128.71, 121.16, 41.04.

HRMS (EI): m/z calculated for $C_{14}H_{11}BrOS^+[M]^+$: 305.9708, found: 305.9707.

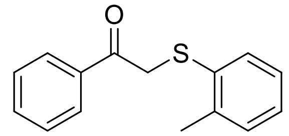

1-phenyl-2-(*o*-tolylthio)ethan-1-one (3-2h)

82% yield, yellow solid. (m.p. 57-60°C). ^{1}H NMR (300 MHz, $CDCl_3$) δ 8.00 – 7.92 (m, 2H), 7.62 – 7.54 (m, 1H), 7.46 (t, J = 7.6 Hz, 2H), 7.39 – 7.32 (m, 1H), 7.21 – 7.10 (m, 3H), 4.25 (s, 2H), 2.39 (s, 3H). ^{13}C NMR (75 MHz, $CDCl_3$) δ 194.02, 138.62, 135.32, 133.94, 133.43, 130.27, 130.14, 128.63, 128.62, 126.99, 126.62, 40.44, 20.43.

HRMS (EI): m/z calculated for $C_{15}H_{14}OS^+[M]^+$: 242.0760, found: 242.0758.

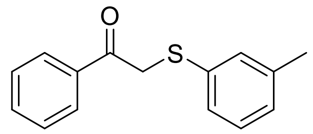

1-phenyl-2-(*m*-tolylthio)ethan-1-one (3-2i)

73% yield, yellow oil. ^{1}H NMR (300 MHz, $CDCl_3$) δ 7.99 – 7.91 (m, 2H), 7.61 – 7.54 (m, 1H), 7.46 (t, J = 7.5 Hz, 2H), 7.24 – 7.13 (m, 3H), 7.04 (d, J = 6.8 Hz, 1H), 4.28 (s, 2H), 2.31 (s, 3H). ^{13}C NMR (75 MHz, $CDCl_3$) δ 194.10, 138.82, 135.36, 134.50, 133.45, 131.01, 128.91, 128.66, 127.93, 127.34, 41.17, 21.30.

HRMS (EI): m/z calculated for $C_{15}H_{14}OS^+[M]^+$: 242.0760, found: 242.0757.

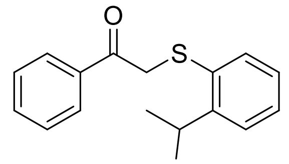

2-((2-isopropylphenyl)thio)-1-phenylethan-1-one (3-2j)

75% yield, yellow oil. ^{1}H NMR (300 MHz, $CDCl_3$) δ 7.92 (d, J = 7.0 Hz, 2H), 7.55 (t, J = 7.4 Hz, 1H), 7.47 – 7.36 (m, 3H), 7.27 – 7.19 (m, 2H), 7.16 – 7.07 (m, 1H), 4.22 (s, 2H), 3.47 (p, J = 6.9 Hz, 1H), 1.16 (d, J = 6.9 Hz, 6H). ^{13}C NMR (75 MHz, $CDCl_3$) δ 194.20, 149.66, 135.47, 133.49, 132.87, 131.51, 128.75, 128.69, 127.82, 126.55, 125.80, 41.75, 30.42, 23.59.

HRMS (EI): m/z calculated for $C_{17}H_{18}OS^+[M]^+$: 270.1073, found: 270.1072.

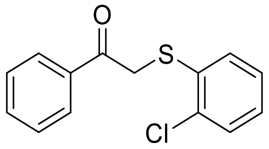

2-((2-chlorophenyl)thio)-1-phenylethan-1-one (3-2k) [5]

67% yield, yellow solid. (m.p. 84-87°C). ^{1}H NMR (300 MHz, $CDCl_3$) δ 8.00 – 7.92 (m, 2H), 7.61 – 7.56 (m, 1H), 7.46 (t, *J* = 7.5 Hz, 2H), 7.40 – 7.36 (m, 2H), 7.22 – 7.12 (m, 2H), 4.33 (s, 2H). ^{13}C NMR (75 MHz, $CDCl_3$) δ 193.79, 135.36, 134.75, 133.99, 133.74, 130.81, 129.92, 128.83, 128.75, 127.99, 127.41, 39.79.

HRMS (EI): m/z calculated for $C_{14}H_{11}ClOS^{+}[M]^{+}$: 262.0214, found: 262.0210.

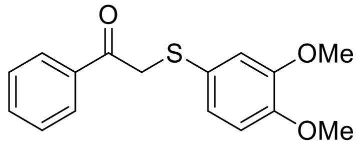

2-((3,4-dimethoxyphenyl)thio)-1-phenylethanone (3-2l)

85% yield, yellow soild. ^{1}H NMR (300 MHz, $CDCl_3$) δ 7.85 – 7.77 (m, 2H), 7.50 – 7.42 (m, 1H), 7.34 (t, *J* = 7.5 Hz, 2H), 6.90 (dd, *J* = 8.3, 2.1 Hz, 1H), 6.83 (d, *J* = 2.1 Hz, 1H), 6.67 (d, *J* = 8.3 Hz, 1H), 4.06 (s, 2H), 3.74 (s, 3H), 3.70 (s, 3H). ^{13}C NMR (75 MHz, $CDCl_3$) δ 194.39, 149.14, 148.89, 135.38, 133.33, 128.67, 128.58, 125.63, 124.87, 115.85, 111.46, 55.83, 42.59.

HRMS (EI): m/z calculated for $C_{16}H_{16}O_3S^{+}[M]^{+}$: 288.0815, found: 288.0812.

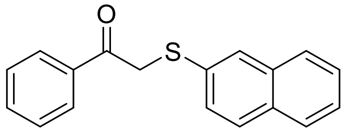

2-(naphthalen-2-ylthio)-1-phenylethan-1-one (3-2m) [3]

77% yield, yellow solid. (m.p. 50-53°C). ^{1}H NMR (300 MHz, $CDCl_3$) δ 7.99 – 7.95 (m, 2H), 7.84 – 7.72 (m, 4H), 7.61 – 7.56 (m, 1H), 7.50 – 7.43 (m, 5H), 4.38 (s, 2H). ^{13}C NMR (75 MHz, $CDCl_3$) δ 194.14, 135.48, 133.76, 133.66, 132.29, 132.27, 128.92, 128.83, 128.79, 128.05, 127.82, 127.45, 126.72, 126.25, 41.23.

HRMS (EI): m/z calculated for $C_{18}H_{14}OS^{+}[M]^{+}$: 278.0760, found: 278.0758.

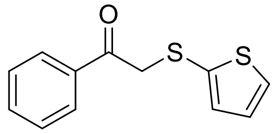

1-phenyl-2-(thiophen-2-ylthio)ethanone (3-2n)

45% yield, purple oil. ^{1}H NMR (300 MHz, $CDCl_3$) δ 7.94 – 7.87 (m, 2H), 7.61 – 7.55 (m, 1H), 7.49 – 7.43 (m, 2H), 7.37 (dd, *J* = 5.4, 1.3 Hz, 1H), 7.12 (dd, *J* = 3.6, 1.3 Hz, 1H), 6.95 (dd, *J* = 5.4, 3.6 Hz, 1H), 4.17 (s, 2H). ^{13}C NMR (75 MHz, $CDCl_3$) δ 194.00, 135.46, 135.44, 133.59, 132.21, 130.73, 128.78, 128.75, 127.80, 45.36.

HRMS (EI): m/z calculated for $C_{12}H_{10}OS_2^{+}[M]^{+}$: 234.0168, found: 234.0166.

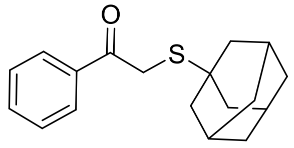

2-((1s, 3s)-adamantan-1-ylthio)-1-phenylethanone (3-2o)

30% yield, yellow oil. ^{1}H NMR (300 MHz, CDCl$_3$) δ 7.92 – 7.86 (m, 2H), 7.52 – 7.45 (m, 1H), 7.39 (dd, J = 8.3, 6.7 Hz, 2H), 3.79 (s, 2H), 2.00 – 1.94 (m, 3H), 1.82 (d, J = 3.0 Hz, 6H), 1.60 (s, 6H). ^{13}C NMR (75 MHz, CDCl$_3$) δ 196.68, 135.69, 133.35, 128.89, 128.68, 46.00, 43.30, 36.22, 33.18, 29.79.

HRMS (ESI): m/z calculated for $C_{18}H_{22}NaOS^+[M+Na]^+$: 309.1284, found: 309.129.

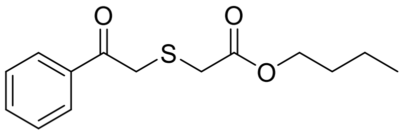

butyl 2-((2-oxo-2-phenylethyl)thio)acetate (3-2p)

35% yield, yellow oil. ^{1}H NMR (300 MHz, CDCl$_3$) δ 7.99 – 7.93 (m, 2H), 7.61 – 7.54 (m, 1H), 7.49 – 7.43 (m, 2H), 4.11 (t, J = 6.7 Hz, 2H), 4.03 (s, 2H), 3.32 (s, 2H), 1.64 – 1.55 (m, 2H), 1.40 – 1.30 (m, 2H), 0.91 (t, J = 7.3 Hz, 3H). ^{13}C NMR (75 MHz, CDCl$_3$) δ 194.10, 170.04, 135.40, 133.64, 128.82, 128.70, 65.48, 37.78, 33.44, 30.60, 19.14, 13.77.

HRMS (EI): m/z calculated for $C_{14}H_{18}O_3S^+[M]^+$: 266.0971, found: 266.0969.

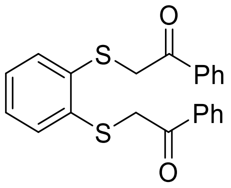

2,2'-(1,2-phenylenebis(sulfanediyl))bis(1-phenylethan-1-one) (3-2q)

36% yield, yellow oil. ^{1}H NMR (300 MHz, CDCl$_3$) δ 7.95 – 7.87 (m, 4H), 7.55 (t, J = 7.4 Hz, 2H), 7.45 – 7.33 (m, 6H), 7.12 – 7.15 (m, 2H), 4.28 (s, 4H). ^{13}C NMR (75 MHz, CDCl$_3$) δ 194.07, 136.18, 135.40, 133.56, 131.14, 128.73, 128.70, 127.79, 40.27.

HRMS (ESI): m/z calculated for $C_{22}H_{18}NaO_2S_2^+[M+Na]^+$: 401.0640, found: 401.0631.

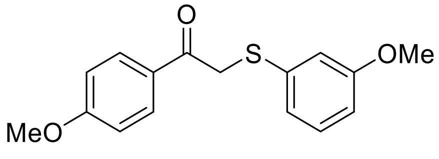

1-(4-methoxyphenyl)-2-((3-methoxyphenyl)thio)ethan-1-one (D)

82% yield, yellow oil. ^{1}H NMR (300 MHz, CDCl$_3$) δ 8.00 – 7.87 (m, 2H), 7.18 (t, J = 7.9 Hz, 1H), 6.91 (d, J = 9.1 Hz, 4H), 6.73 (dd, J = 8.3, 1.6 Hz, 1H), 4.25 (s, 2H), 3.85 (s, 3H), 3.75 (s, 3H). ^{13}C NMR (75 MHz, CDCl$_3$) δ 192.73, 163.83, 159.82, 136.49, 131.06, 129.88, 128.34, 121.97, 115.11, 113.89, 112.67, 55.55, 55.28, 40.74.

HRMS (EI): m/z calculated for $C_{16}H_{16}O_3S^+[M]^+$: 288.0815, found: 288.0812.

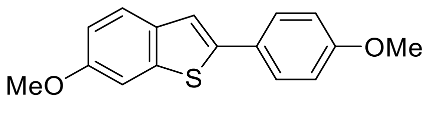

6-methoxy-2-(4-methoxyphenyl)benzo[*b*]thiophene (E)

87% yield, white solid. (m.p. 163-165°C). ^{1}H NMR (300 MHz, CDCl$_3$) δ 7.66 – 7.54 (m, 3H), 7.37 – 7.26 (m, 2H), 7.01 – 6.89 (m, 3H), 3.88 (s, 3H), 3.85 (s, 3H). ^{13}C NMR (75 MHz, CDCl$_3$) δ 159.59, 157.30, 141.64, 140.74, 135.03, 127.55, 127.40, 124.03, 117.89, 114.49, 114.44, 105.00, 55.76, 55.53.

HRMS (EI): m/z calculated for $C_{15}H_{14}O_2S^+$[M]$^+$: 270.0709, found: 270.0707.

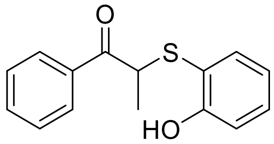

2-((2-hydroxyphenyl)thio)-1-phenylpropan-1-one (F)

75% yield, yellow oil. ^{1}H NMR (300 MHz, $CDCl_3$) δ 7.98 – 7.82 (m, 2H), 7.60 – 7.51 (m, 1H), 7.43 (t, *J* = 7.5 Hz, 2H), 7.35 – 7.22 (m, 2H), 7.05 (s, 1H), 6.94 (dd, *J* = 8.2, 1.4 Hz, 1H), 6.81 (td, *J* = 7.5, 1.3 Hz, 1H), 4.62 (q, *J* = 7.0 Hz, 1H), 1.48 (d, *J* = 7.0 Hz, 3H). ^{13}C NMR (75 MHz, $CDCl_3$) δ 197.33, 158.22, 137.71, 135.04, 133.62, 132.31, 128.82, 128.72, 120.67, 115.47, 115.32, 47.24, 17.32.

HRMS (EI): m/z calculated for $C_{15}H_{14}OS^+$[M]$^+$: 258.0709, found: 258.0706.

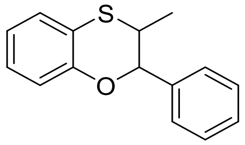

3-methyl-2-phenyl-2,3-dihydrobenzo[*b*][1, 4]oxathiine (G)

88% yield, yellow oil. ^{1}H NMR (300 MHz, $CDCl_3$) δ 7.50 – 7.32 (m, 5H), 7.16 – 6.90 (m, 4H), 5.50 (s, 1H), 3.43 (q, J = 7.1 Hz, 1H), 1.26 (d, J = 6.9 Hz, 3H). ^{13}C NMR (75 MHz, $CDCl_3$) δ 151.49, 139.38, 128.49, 127.92, 127.85, 125.91, 125.64, 121.99, 118.53, 116.90, 78.97, 38.26, 15.20.

HRMS (EI): m/z calculated for $C_{15}H_{14}OS^+$[M]$^+$: 242.0760, found: 242.0756.

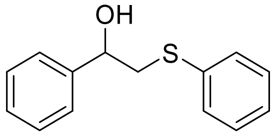

1-phenyl-2-(phenylthio)ethan-1-ol (H) [1]

88% yield, yellow oil. ^{1}H NMR (300 MHz, $CDCl_3$) δ 7.42 – 7.19 (m, 10H), 4.69 (dd, *J* = 9.4, 3.6 Hz, 1H), 3.29 (dd, *J* = 13.8, 3.6 Hz, 1H), 3.07 (dd, *J* = 13.8, 9.4 Hz, 1H). ^{13}C NMR (75 MHz, $CDCl_3$) δ 142.22, 134.98, 130.21, 129.21, 128.63, 128.06, 126.82, 125.94, 71.72, 43.96.

HRMS (ESI): m/z calculated for $C_{14}H_{14}NaOS^+$[M+Na]$^+$: 253.0658, found: 253.0658.

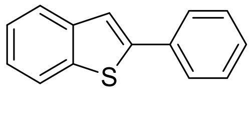

2-phenylbenzo[b]thiophene (I)

83% yield, white solid. (m.p. 166-170°C). ^{1}H NMR (300 MHz, $CDCl_3$) δ 7.90 – 7.67 (m, 4H), 7.56 (s, 1H), 7.48 – 7.29 (m, 5H). ^{13}C NMR (75 MHz, $CDCl_3$) δ 144.38, 140.83, 139.63, 134.43, 129.09, 128.41, 126.64, 124.65, 124.46, 123.70, 122.41, 119.59.

HRMS (EI): m/z calculated for $C_{14}H_{10}S^+$[M]$^+$: 210.0498, found: 210.0496.

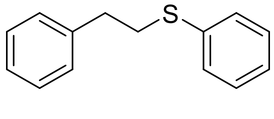

phenethyl(phenyl)sulfane (J)

84% yield, white oil. ^{1}H NMR (300 MHz, $CDCl_3$) δ 7.41 – 7.09 (m, 10H), 3.15 (t, *J* = 7.8 Hz,

2H), 2.90 (t, J = 7.9 Hz, 2H). ^{13}C NMR (75 MHz, $CDCl_3$) δ 140.28, 136.45, 129.22, 129.02, 128.60, 126.54, 126.04, 35.69, 35.12.
HRMS (EI): m/z calculated for $C_{14}H_{14}S^+[M]^+$: 214.0811, found: 214.0812.

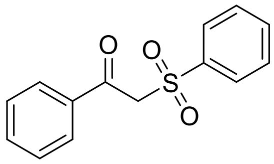

1-phenyl-2-(phenylsulfonyl)ethan-1-one (K)
75% yield, yellow solid. (m.p. 91-95°C).^{1}H NMR (300 MHz, $CDCl_3$) δ 7.90 – 7.86 (m, 4H), 7.59 – 7.35 (m, 6H), 4.75 (s, 2H). ^{13}C NMR (75 MHz, $CDCl_3$) δ 188.01, 138.68, 135.54, 134.24, 134.11, 129.10, 128.72, 128.36, 63.10.
HRMS (ESI): m/z calculated for $C_{14}H_{12}NaO_3S^+[M+Na]^+$: 283.0399, found: 283.0400.

^{13}C NMR Signal Assignment in Compound 3-1a

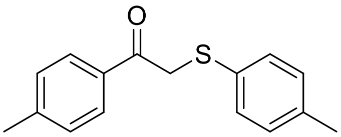

1-(*p*-tolyl)-2-(*p*-tolylthio)ethan-1-one (3-1a)
^{13}C NMR (75 MHz, $CDCl_3$)
The two methyl carbon atoms, 21.69, 21.08; The 12 carbon atoms of two benzene ring, 144.26, 137.29, 132.86, 131.25, 131.06, 129.82, 129.32, 128.80. Carbon atom C-5 δ 193.81; Carbon atom C-6, 41.70.

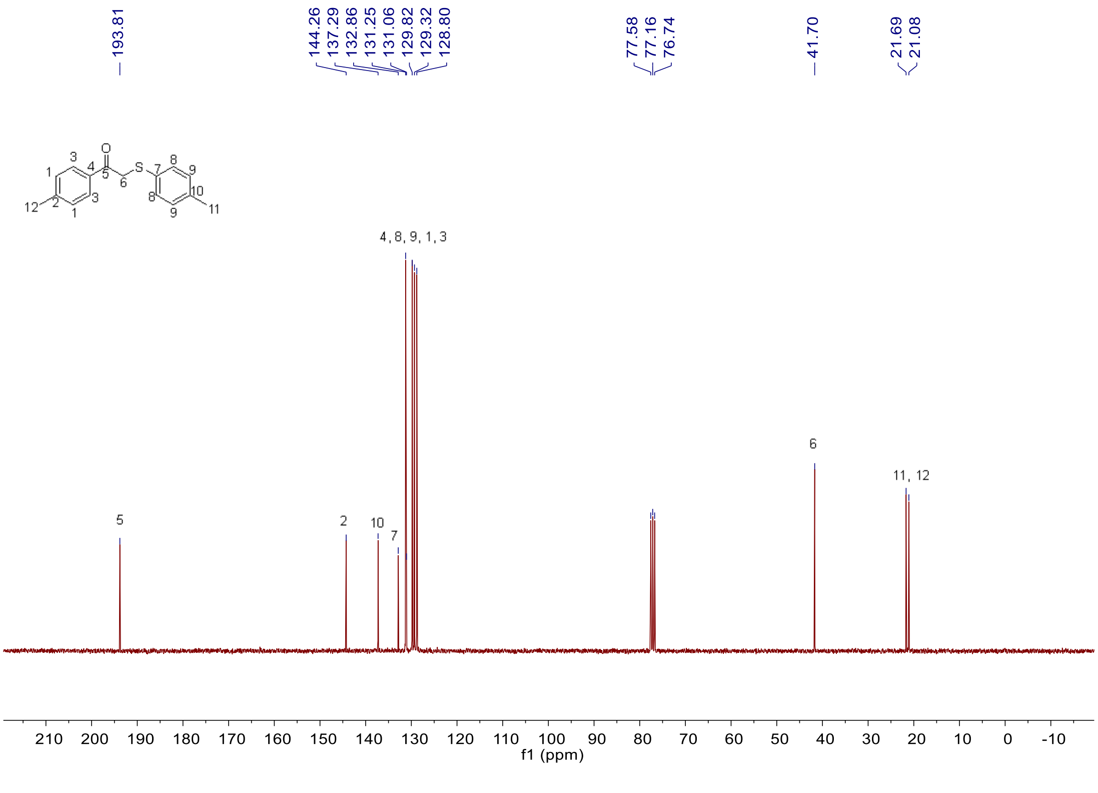

Copies of ^{1}H NMR, ^{13}C NMR and ^{19}F NMR Spectrum

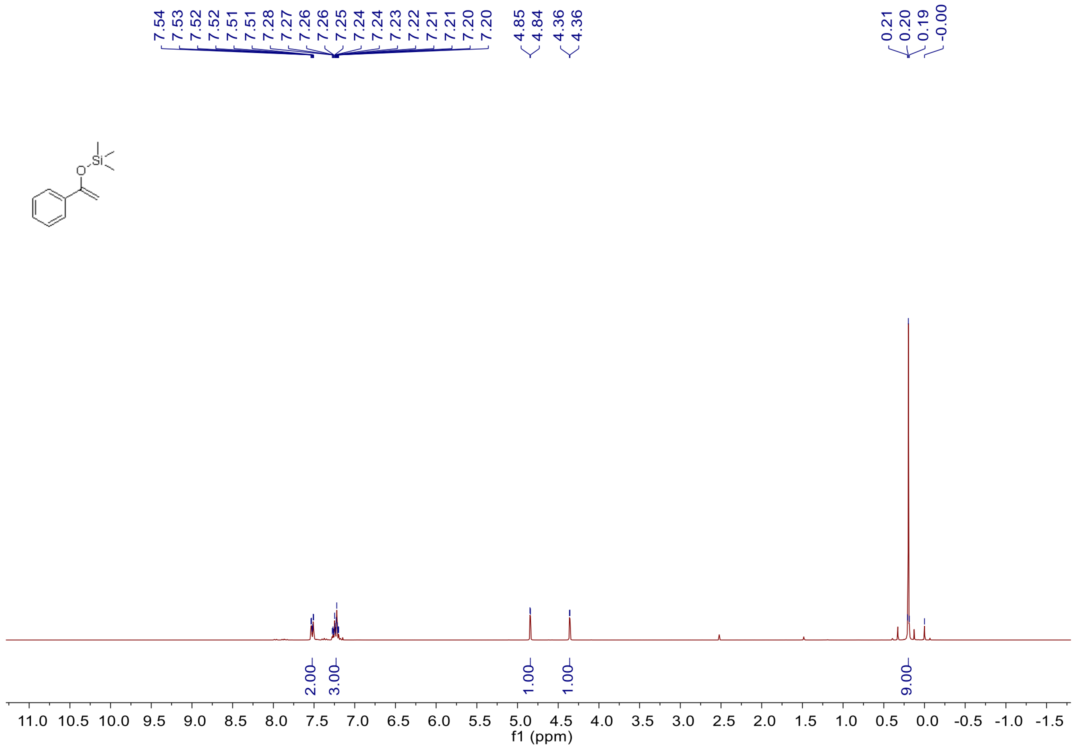

^{1}H NMR of **1a**

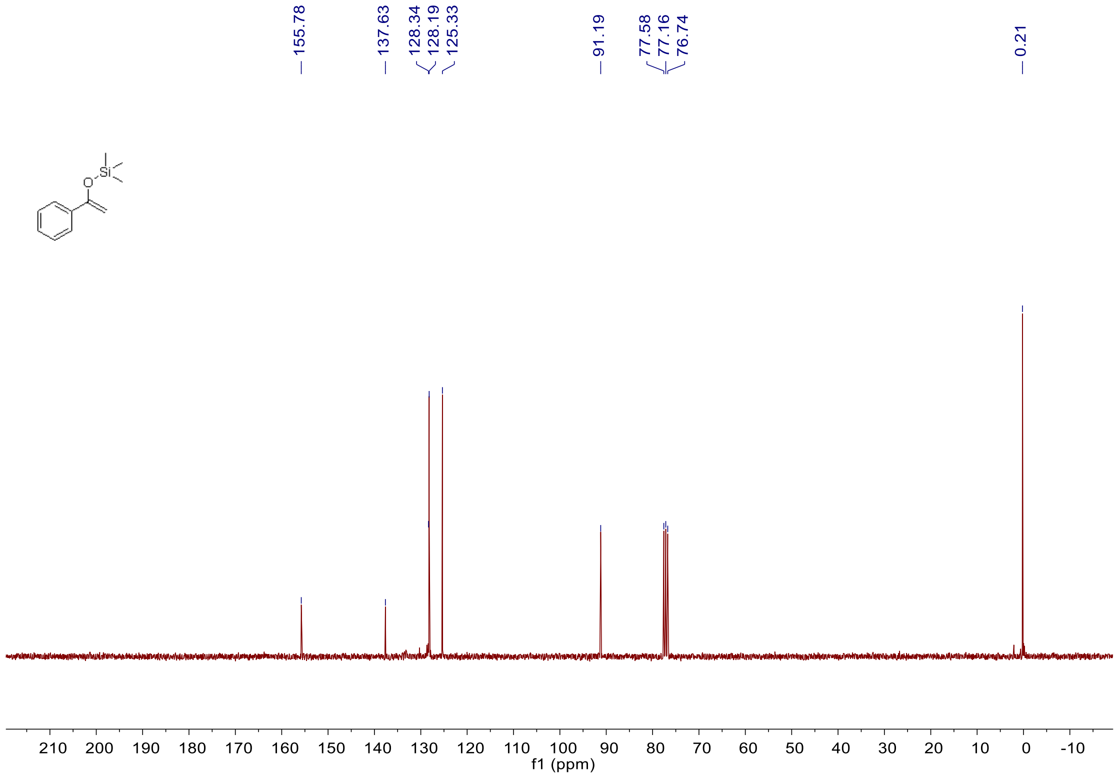

^{13}C NMR of **1a**

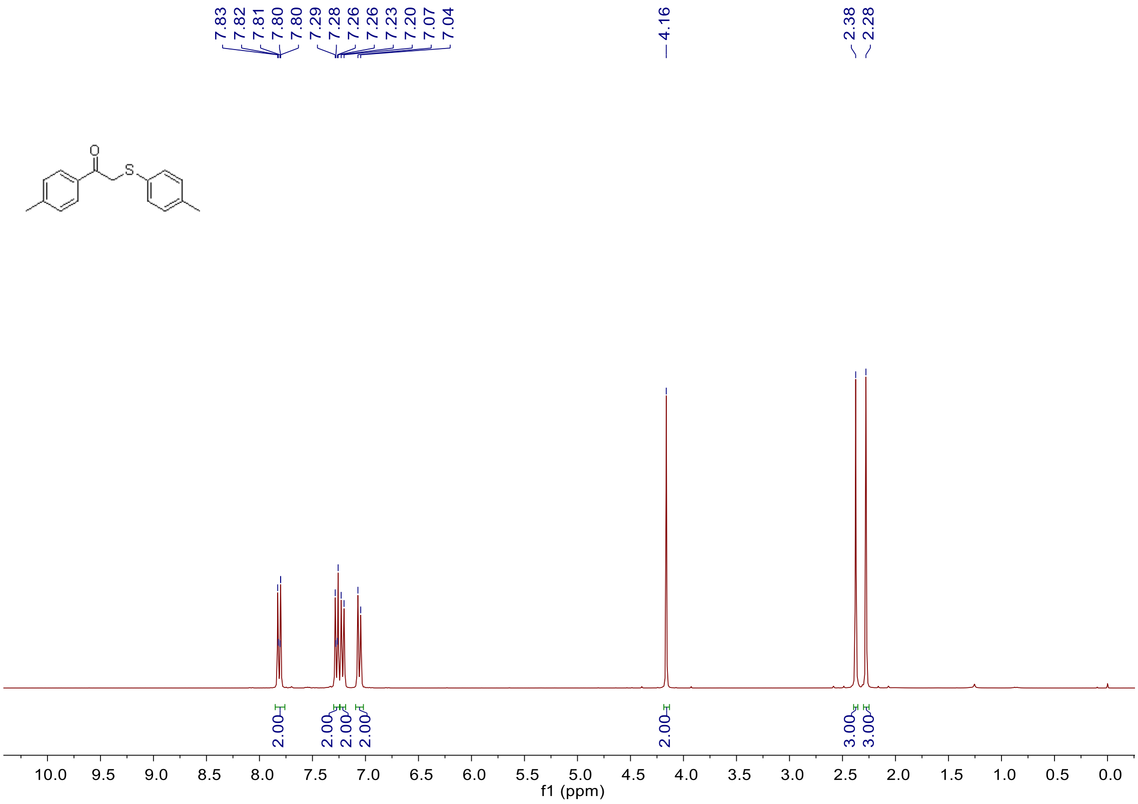

^{1}H NMR of **3-1a**

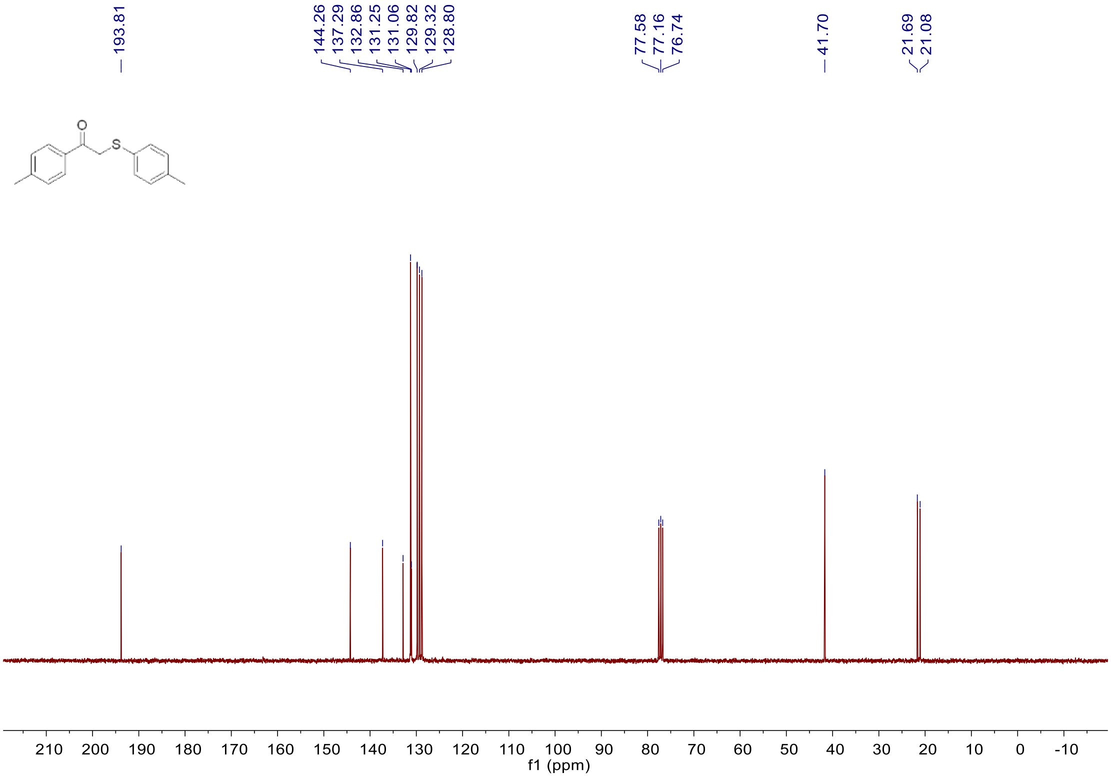

^{13}C NMR of **3-1a**

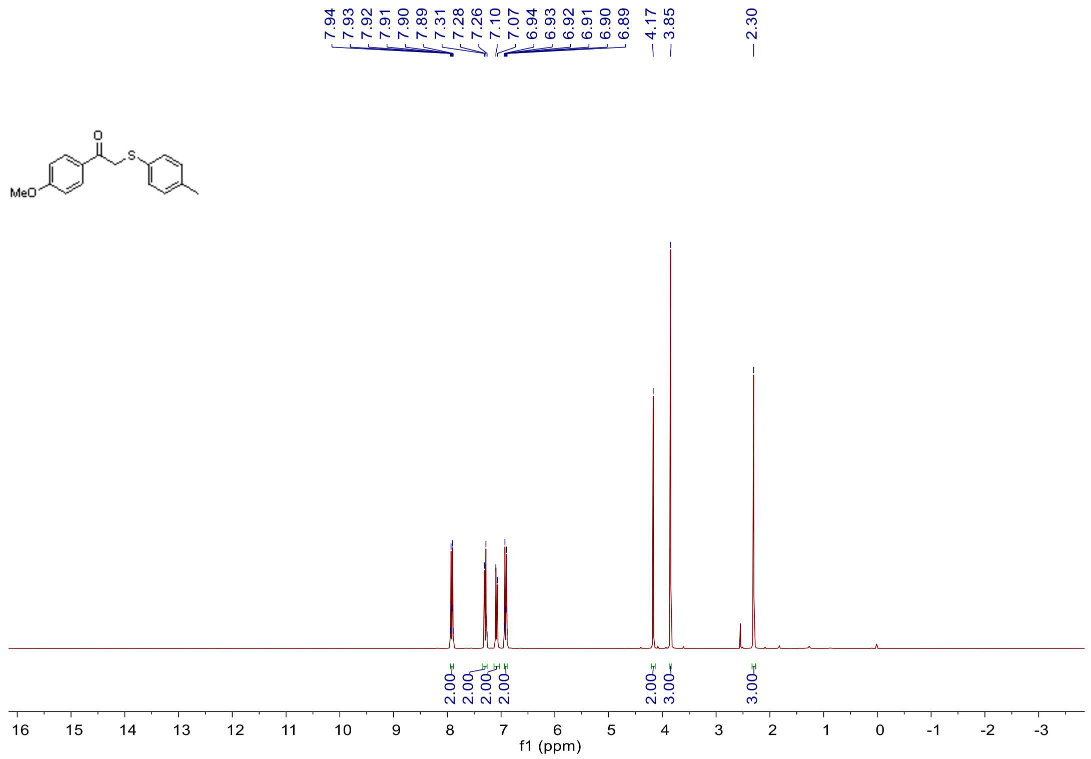

^{1}H NMR of **3-1b**

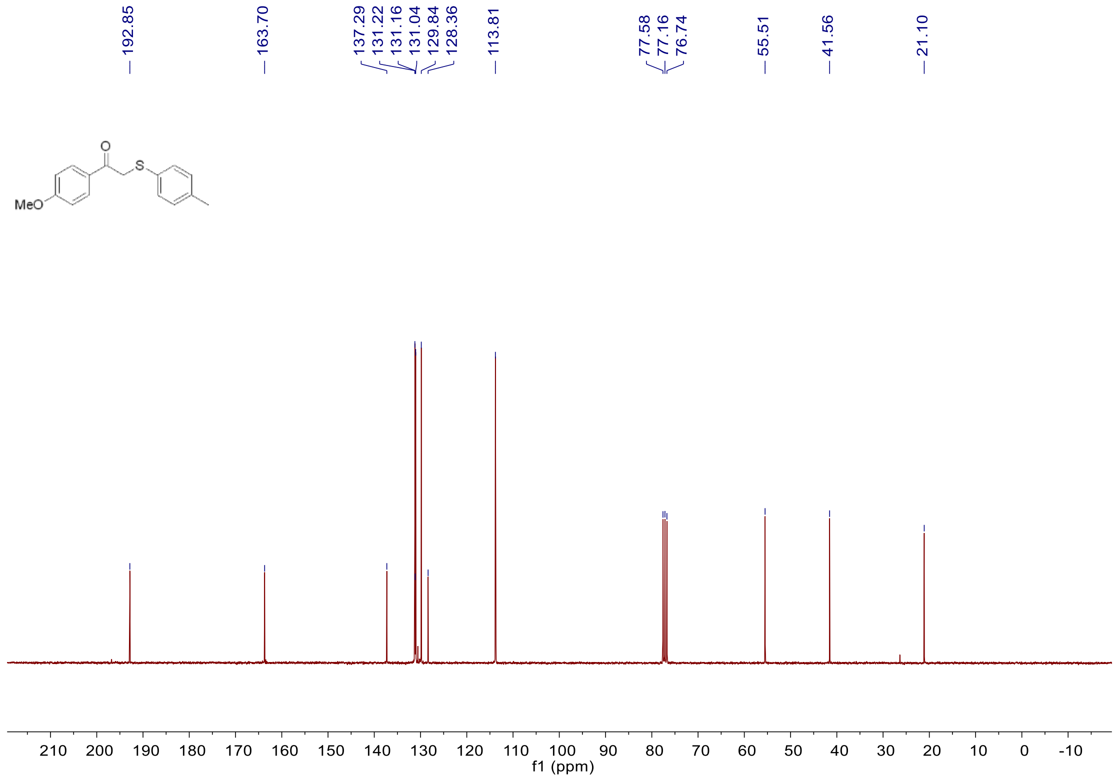

^{13}C NMR of **3-1b**

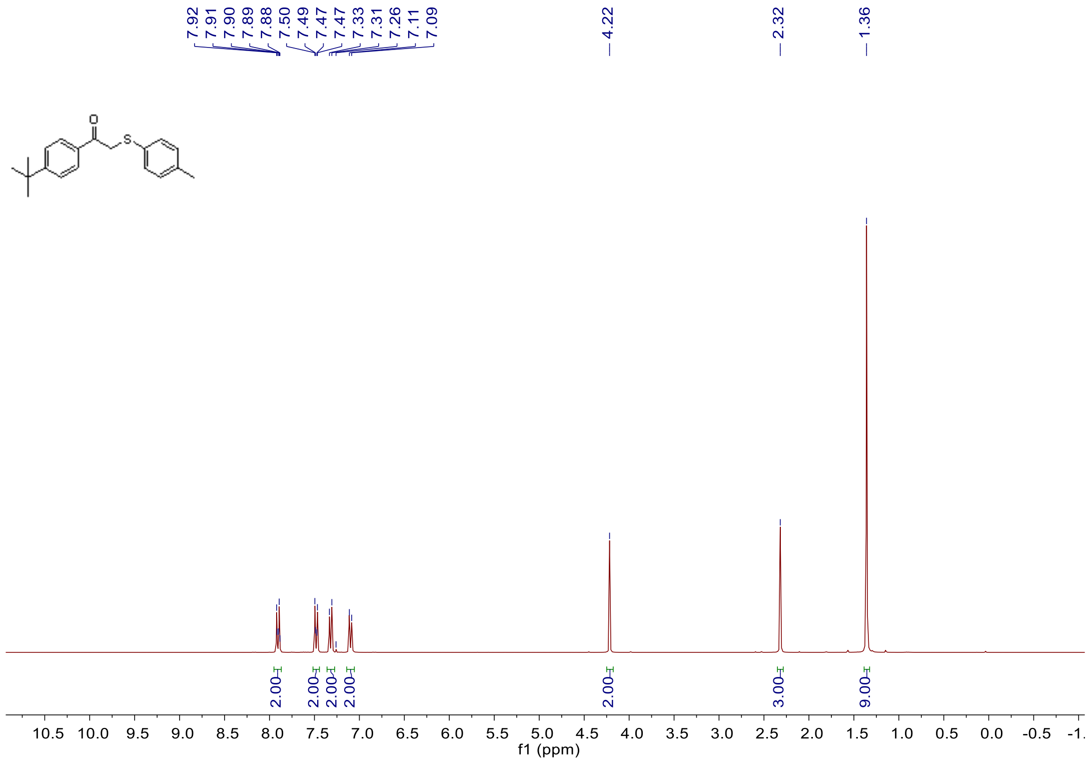

^{1}H NMR of **3-1c**

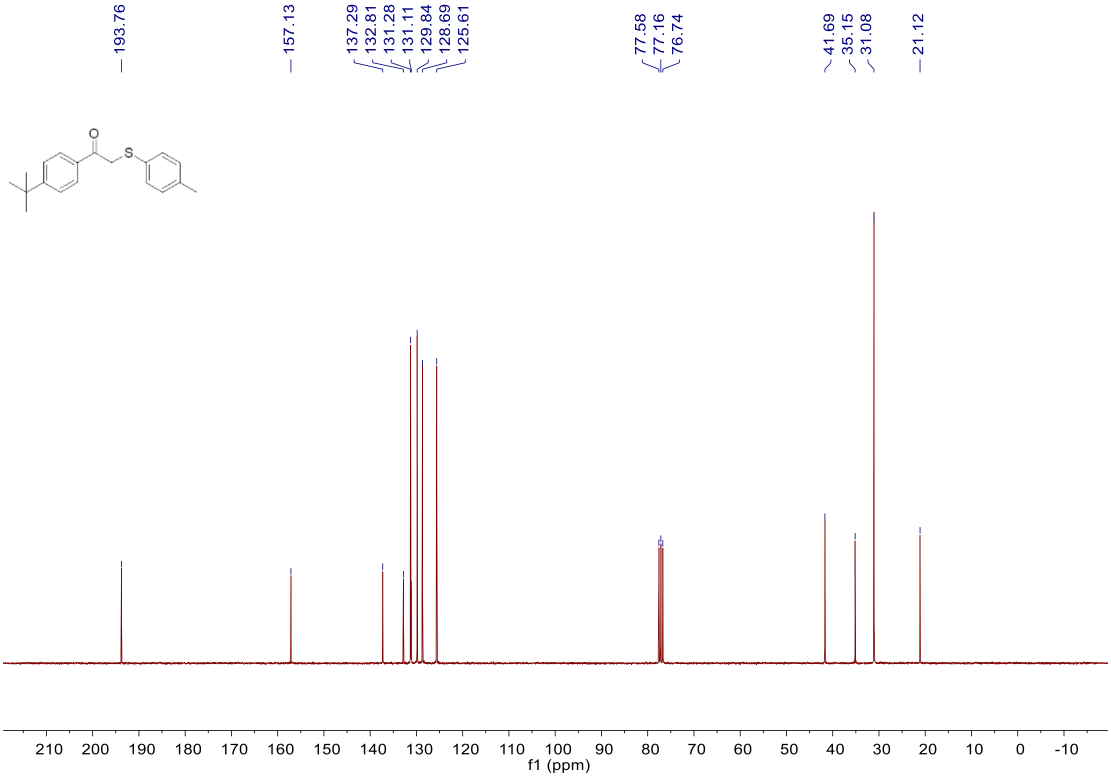

^{13}C NMR of **3-1c**

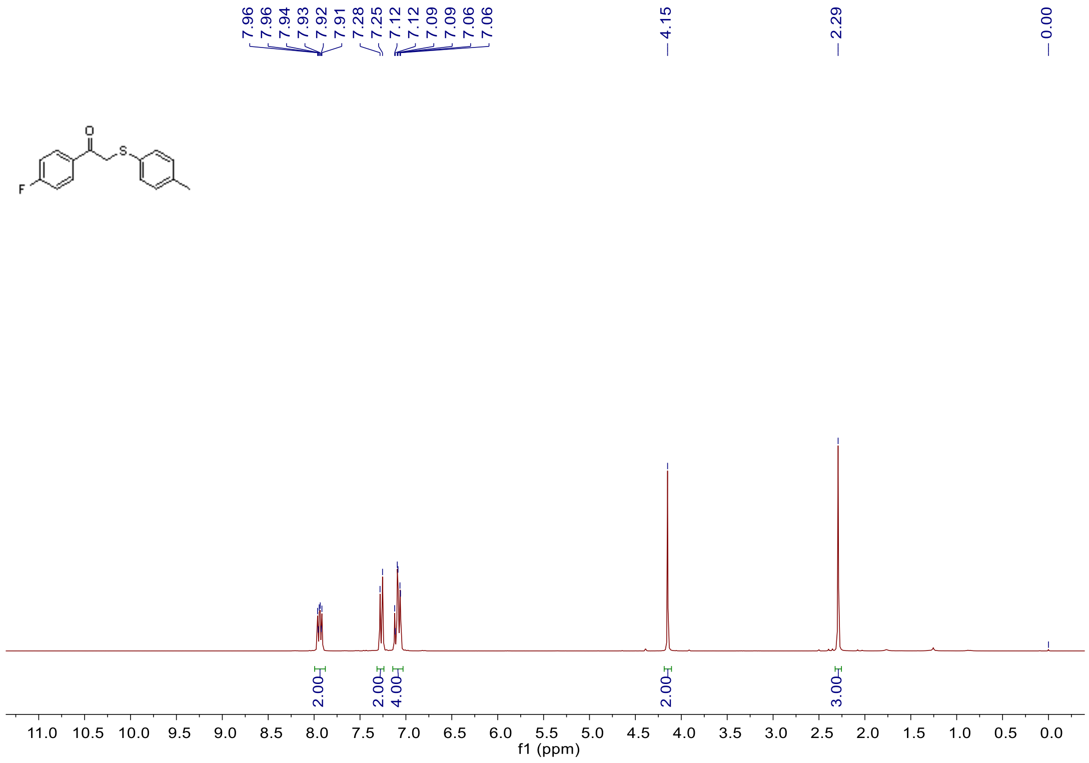

^{1}H NMR of **3-1d**

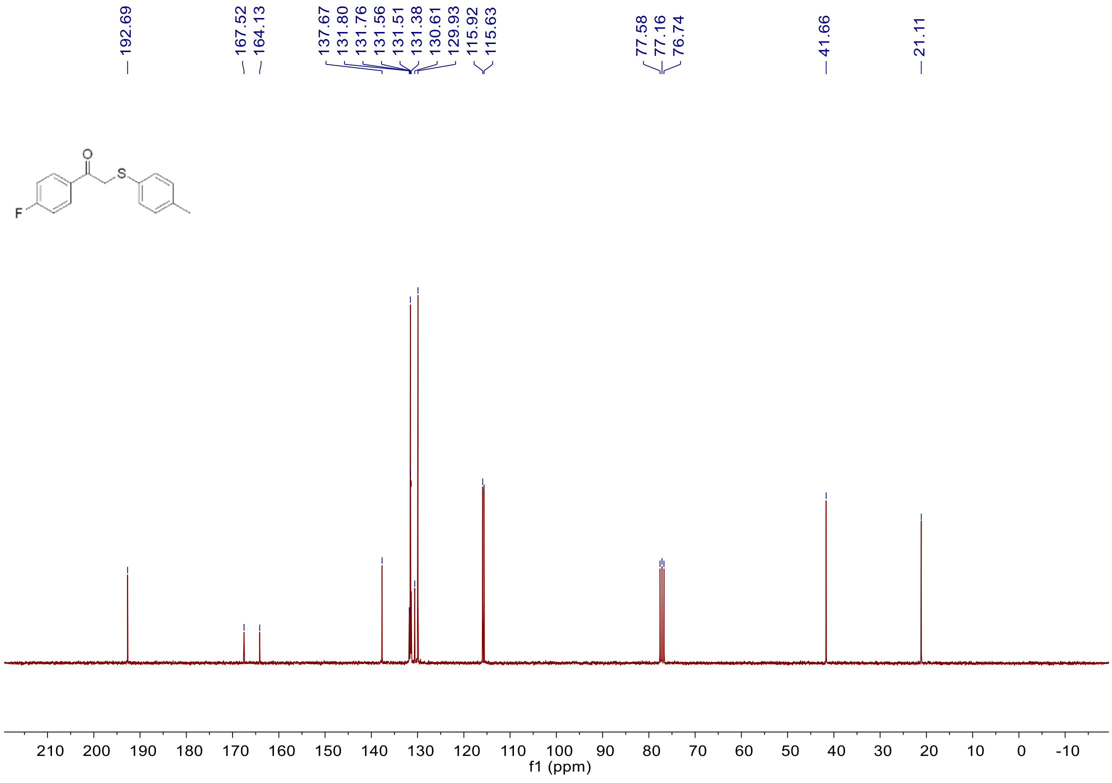

^{13}C NMR of **3-1d**

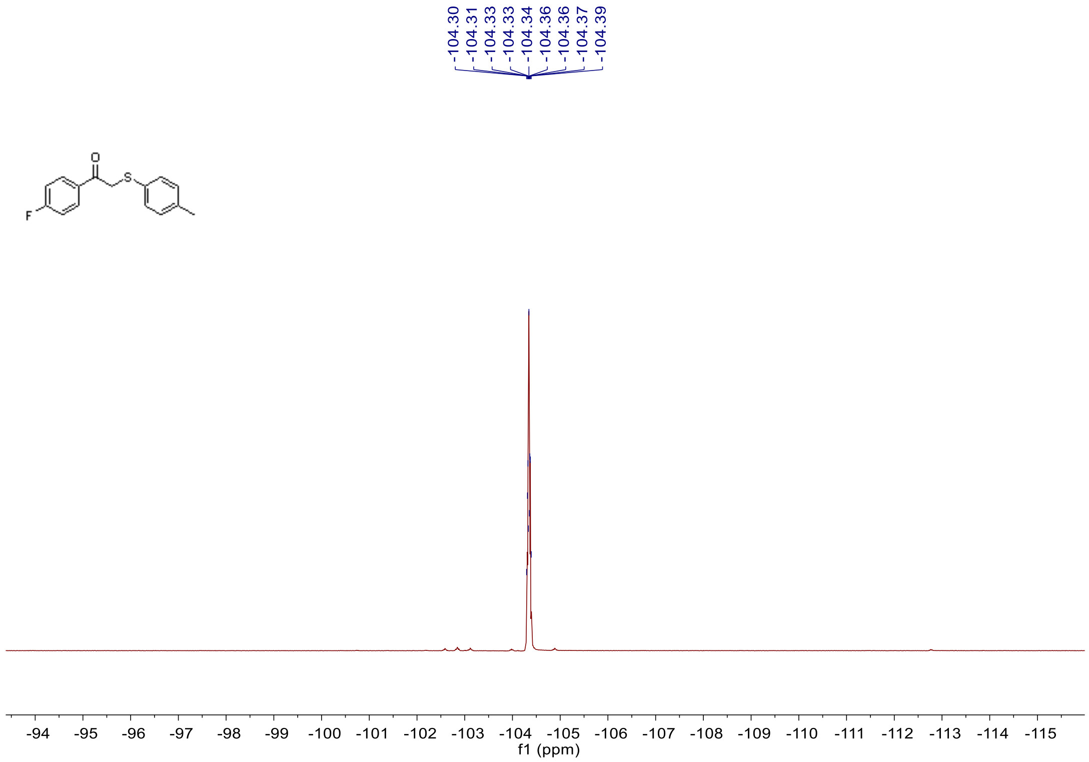

^{19}F NMR of **3-1d**

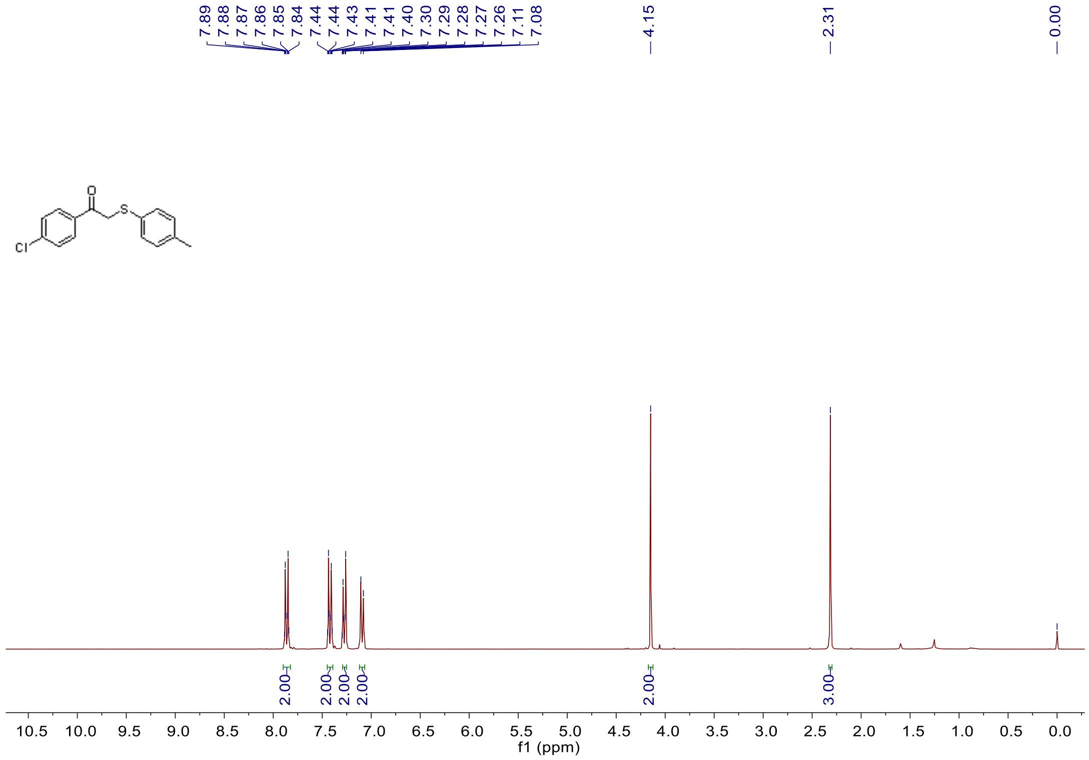

^{1}H NMR of **3-1e**

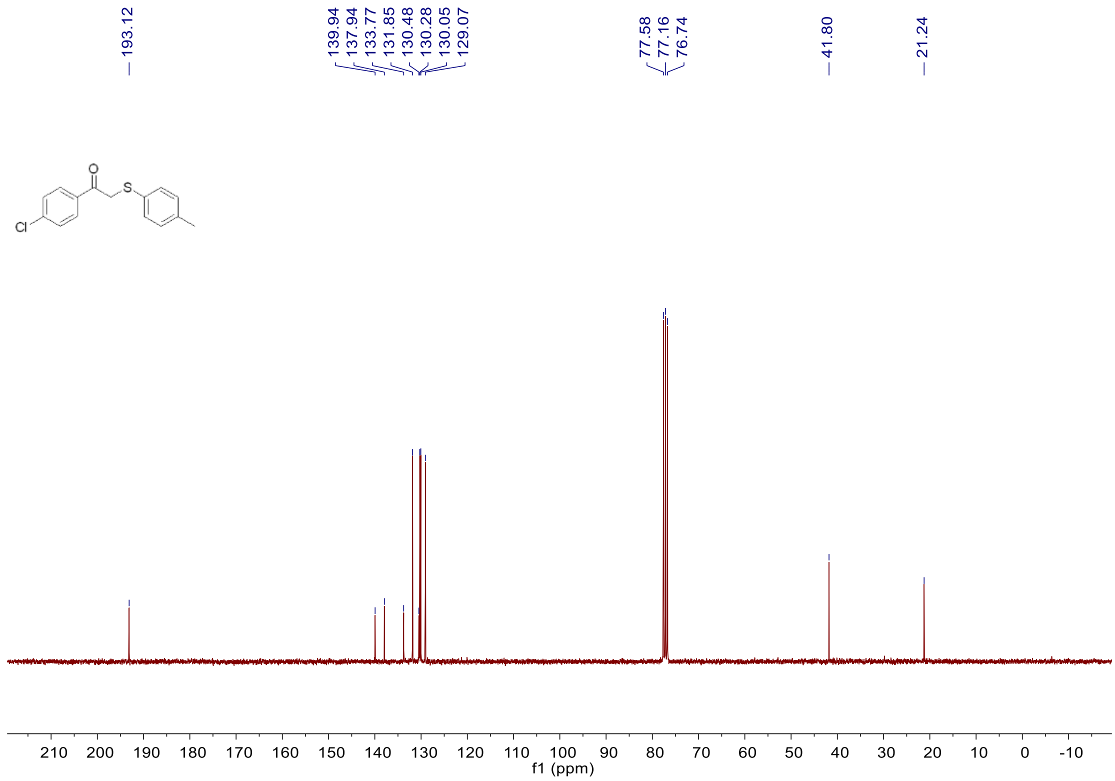

^{13}C NMR of **3-1e**

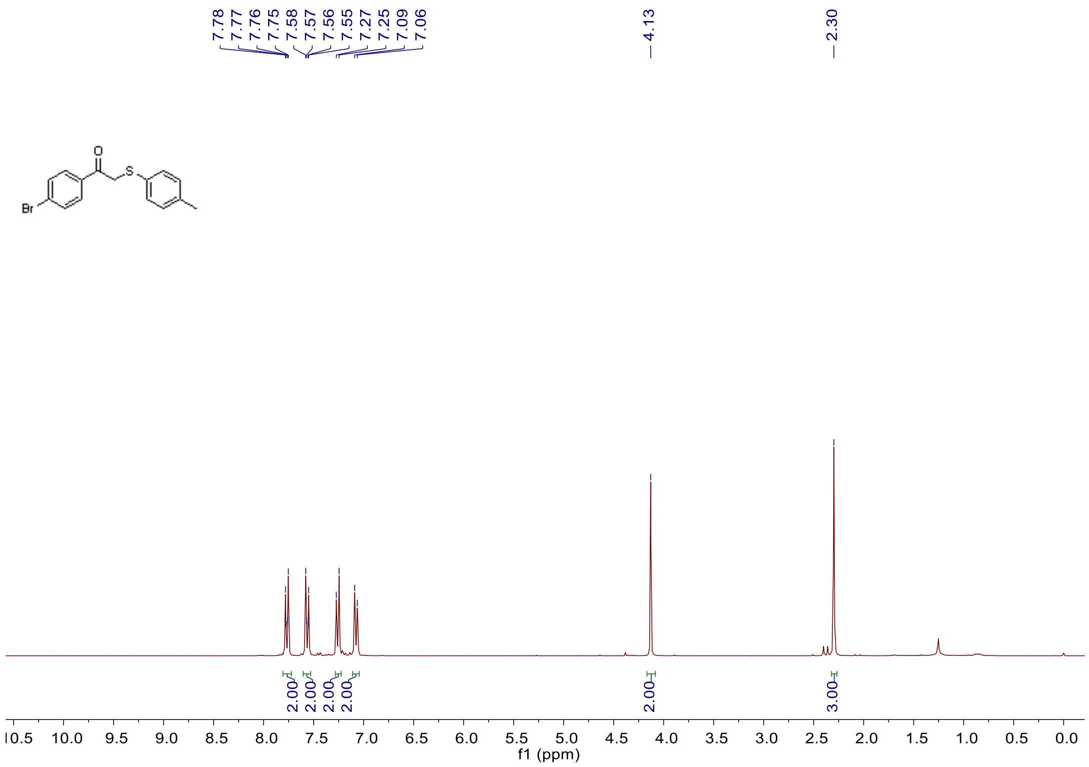

^{1}H NMR of **3-1f**

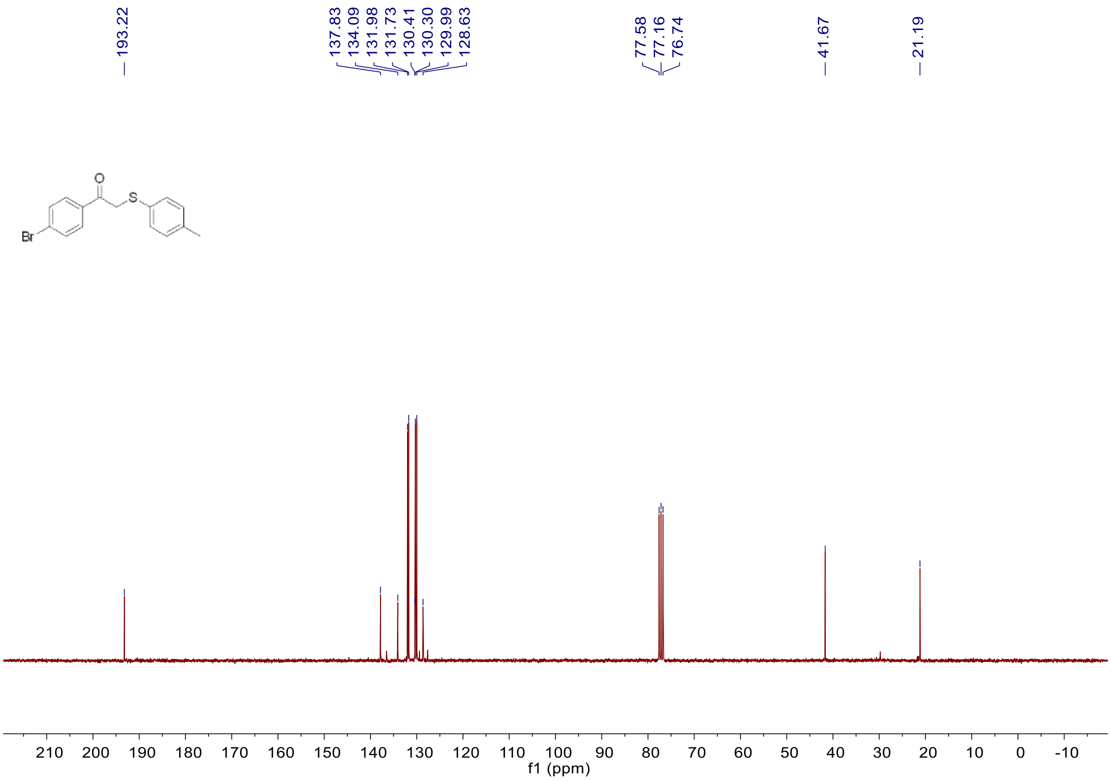

^{13}C NMR of **3-1f**

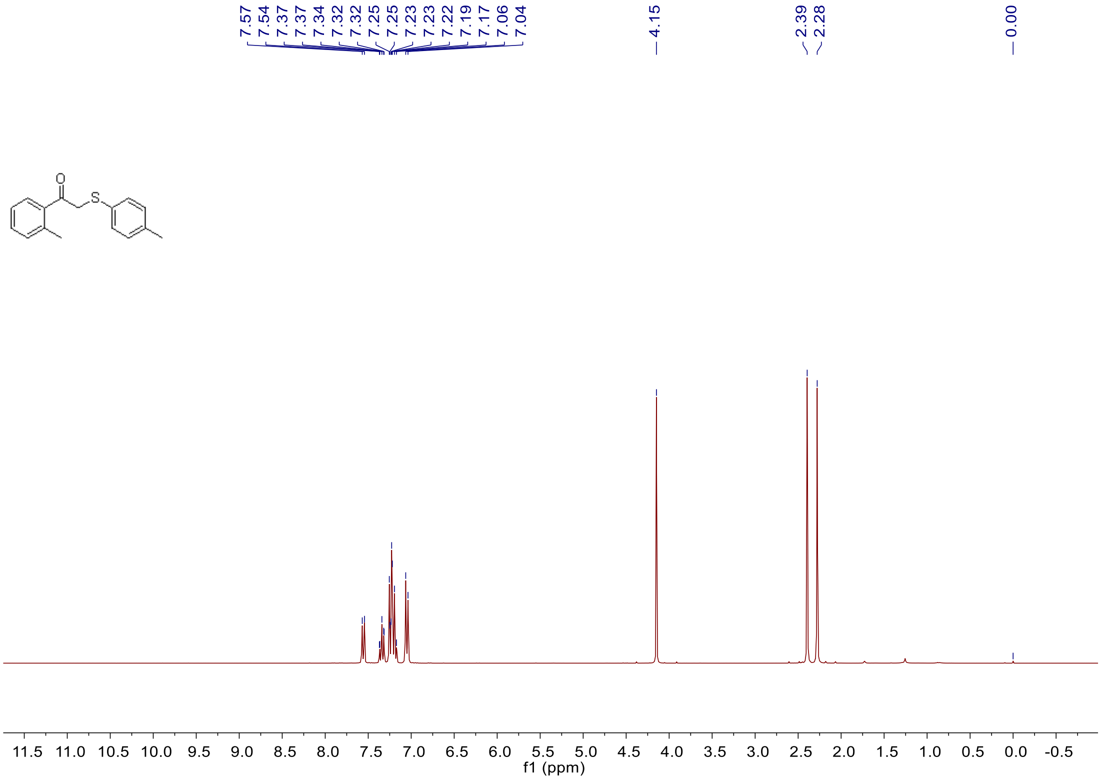

^{1}H NMR of **3-1g**

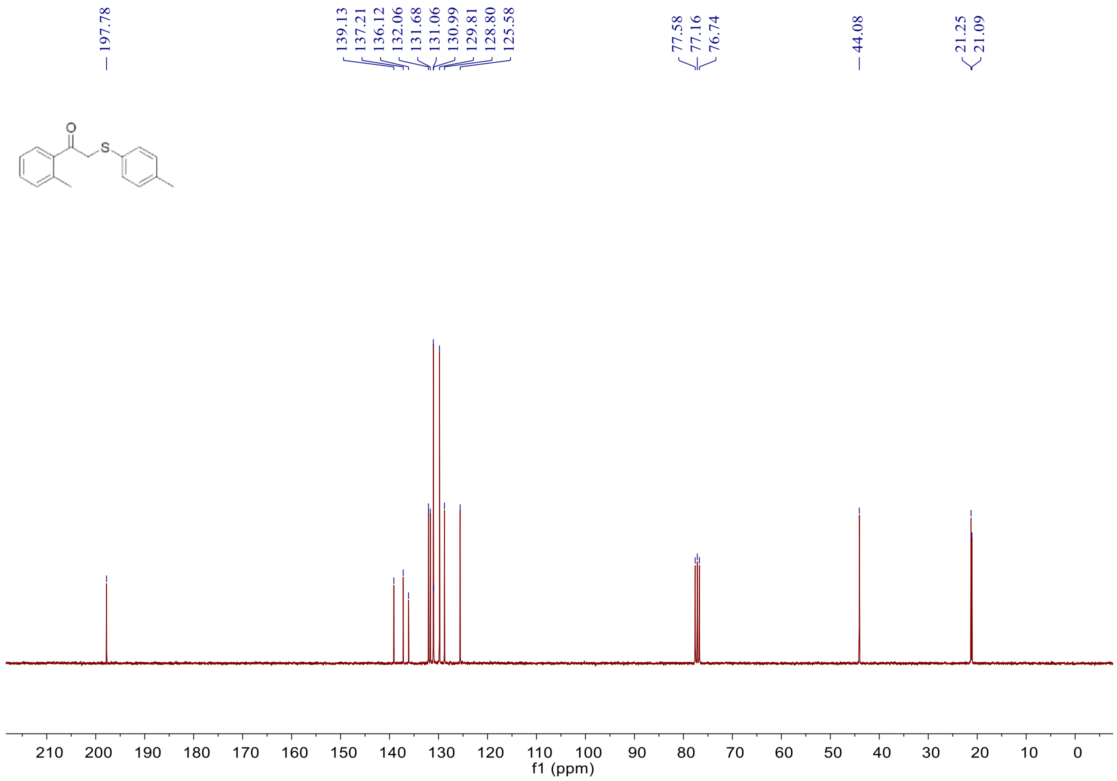

^{13}C NMR of **3-1g**

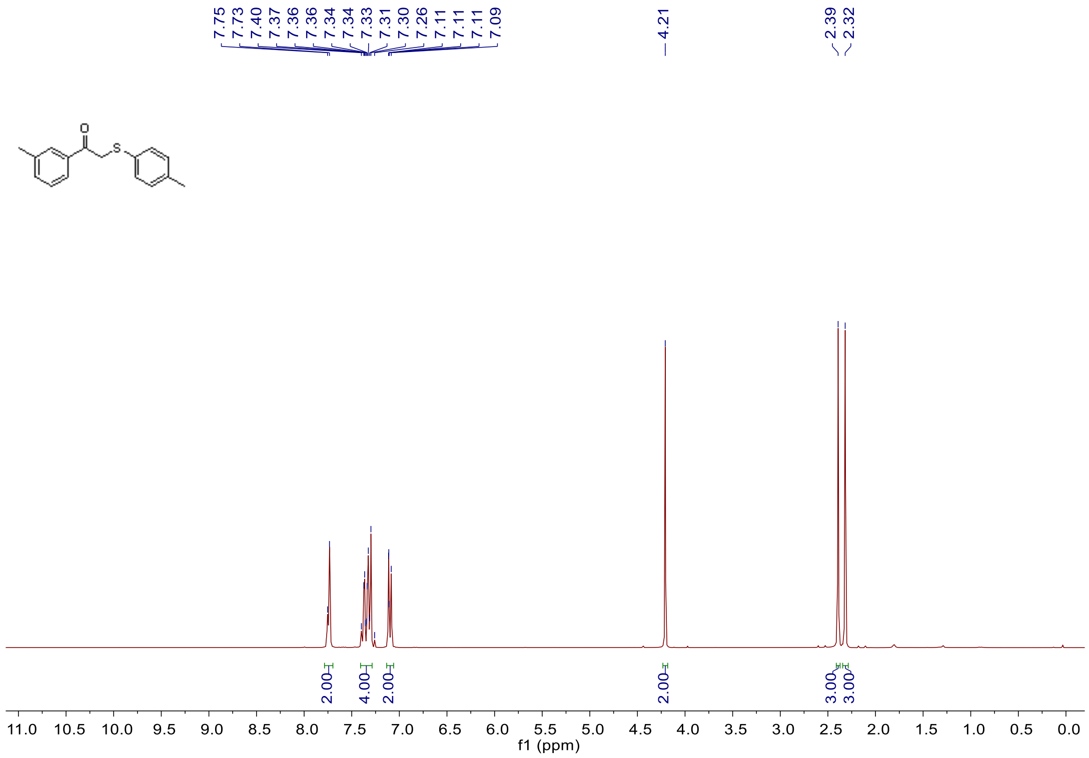

^{1}H NMR of **3-1h**

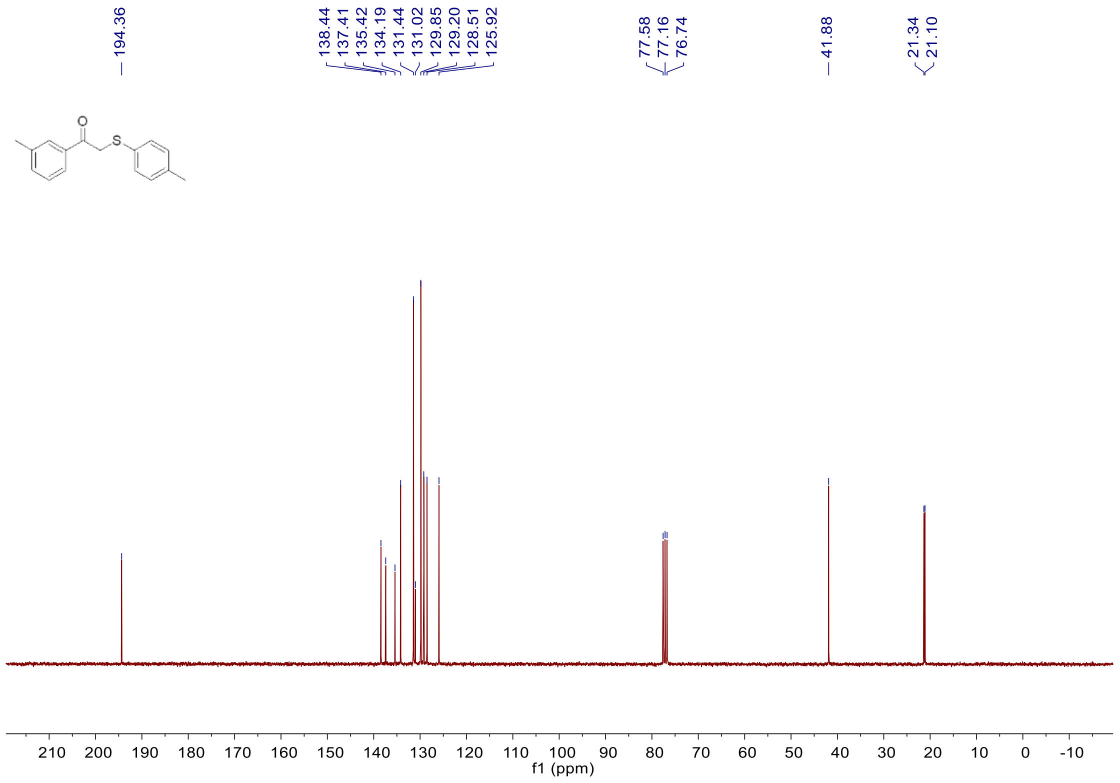

^{13}C NMR of **3-1h**

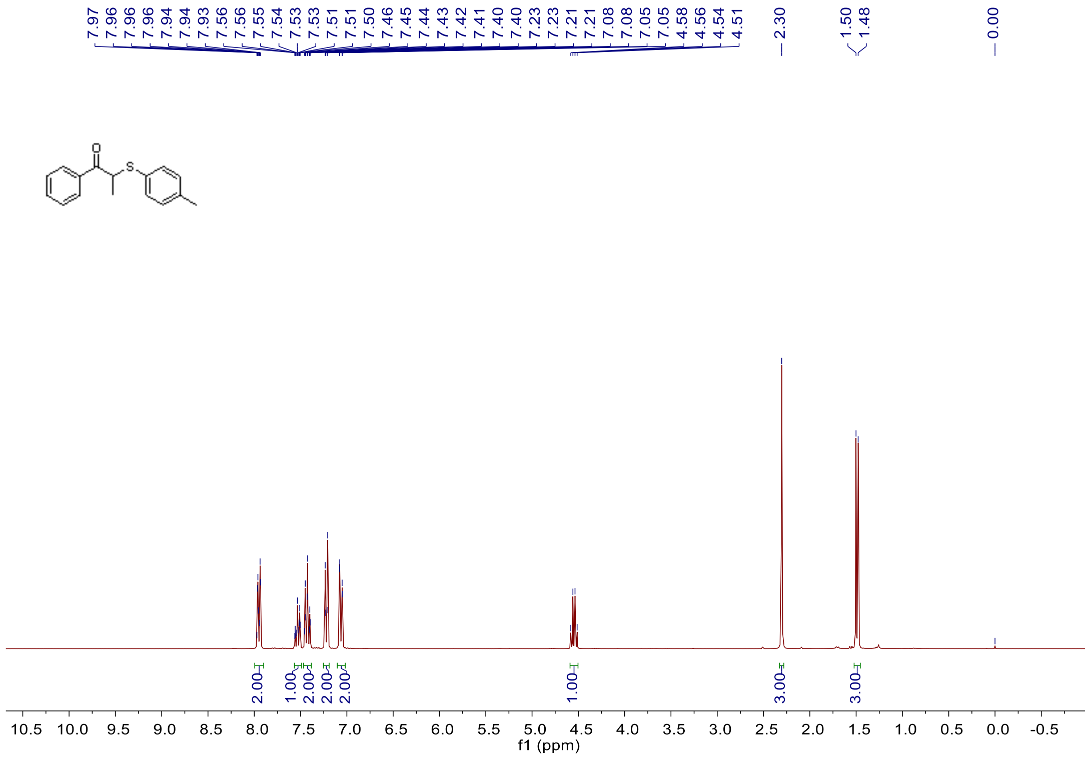

^{1}H NMR of **3-1i**

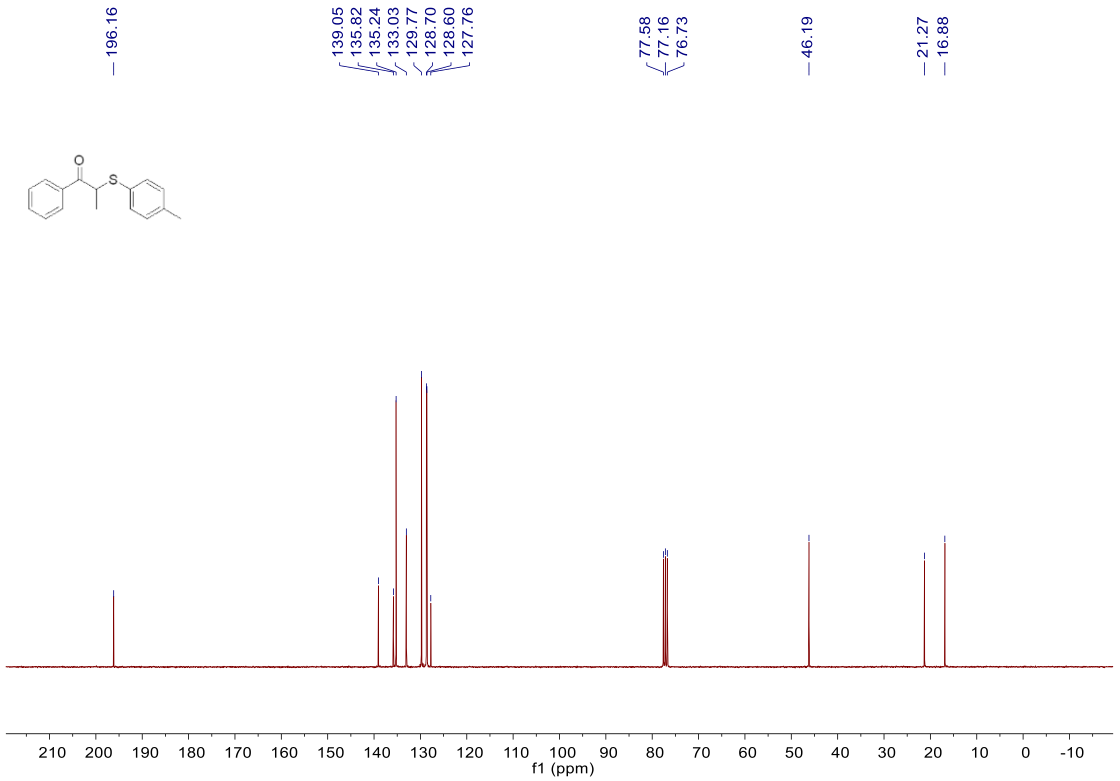

^{13}C NMR of **3-1i**

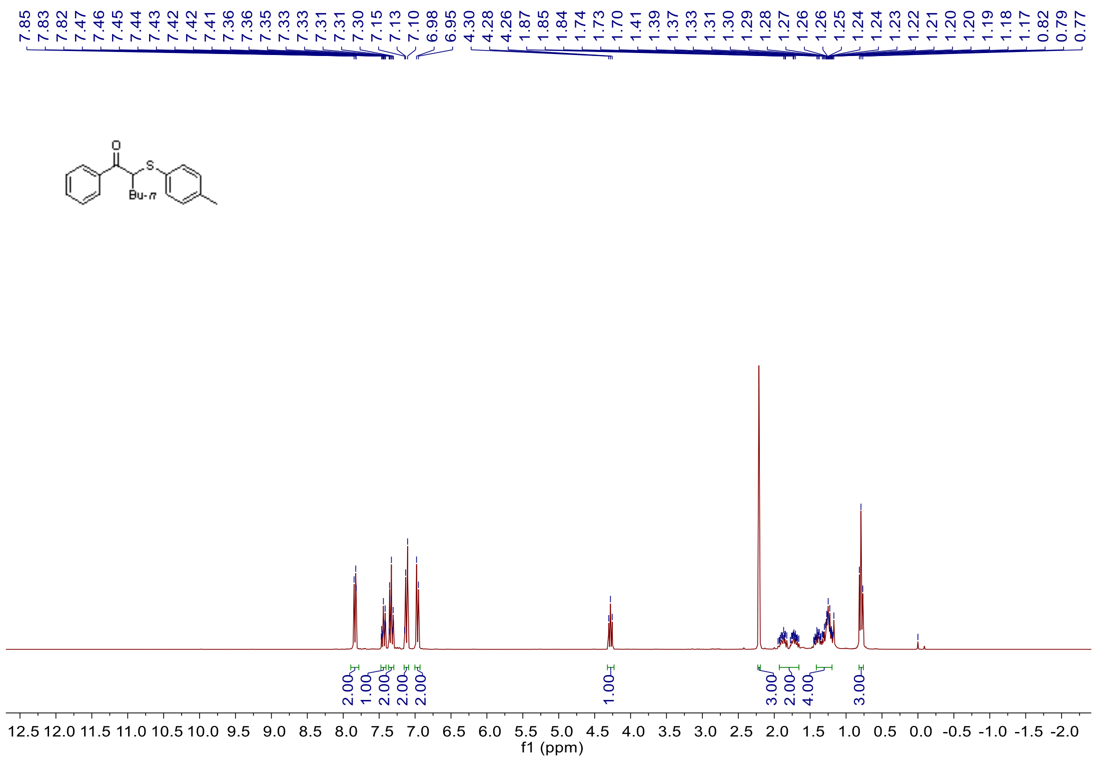

^{1}H NMR of **3-1j**

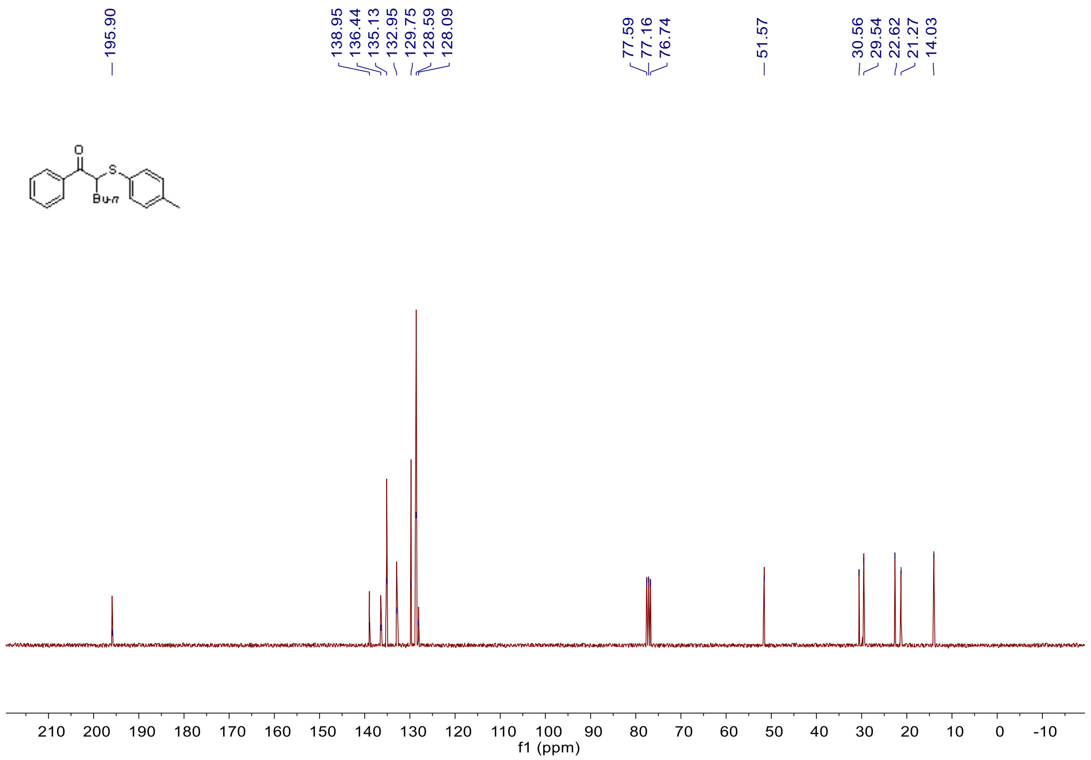

^{13}C NMR of **3-1j**

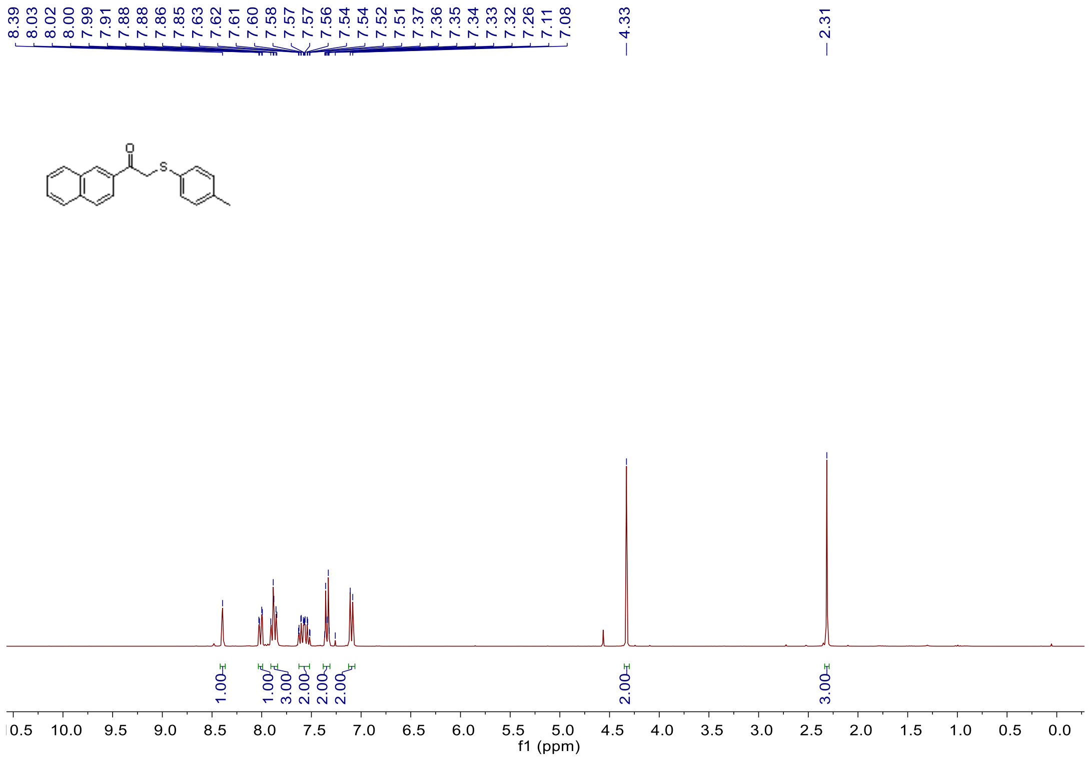

^{1}H NMR of **3-1k**

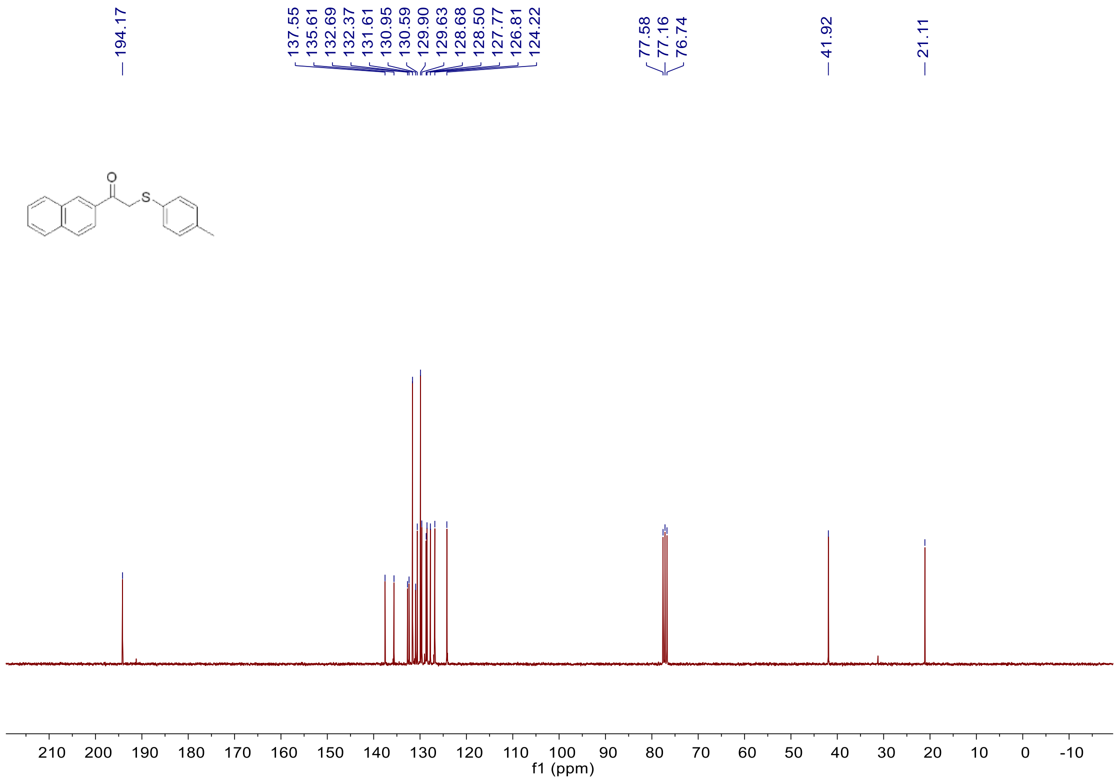

^{13}C NMR of **3-1k**

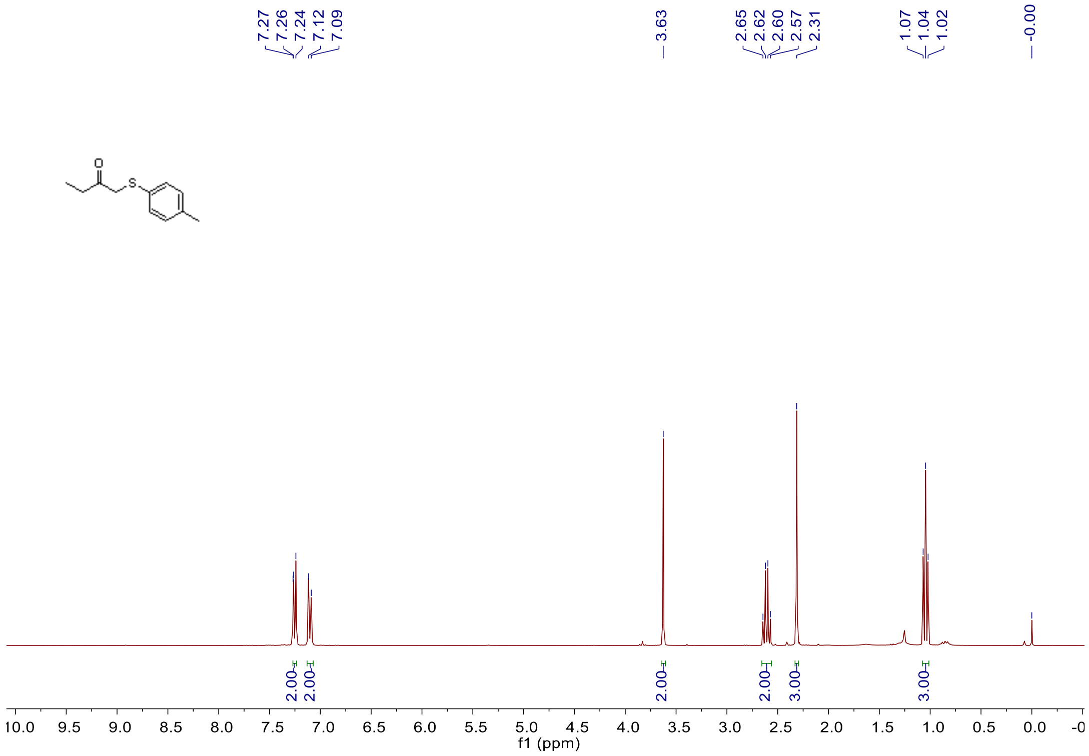

^{1}H NMR of **3-1l**

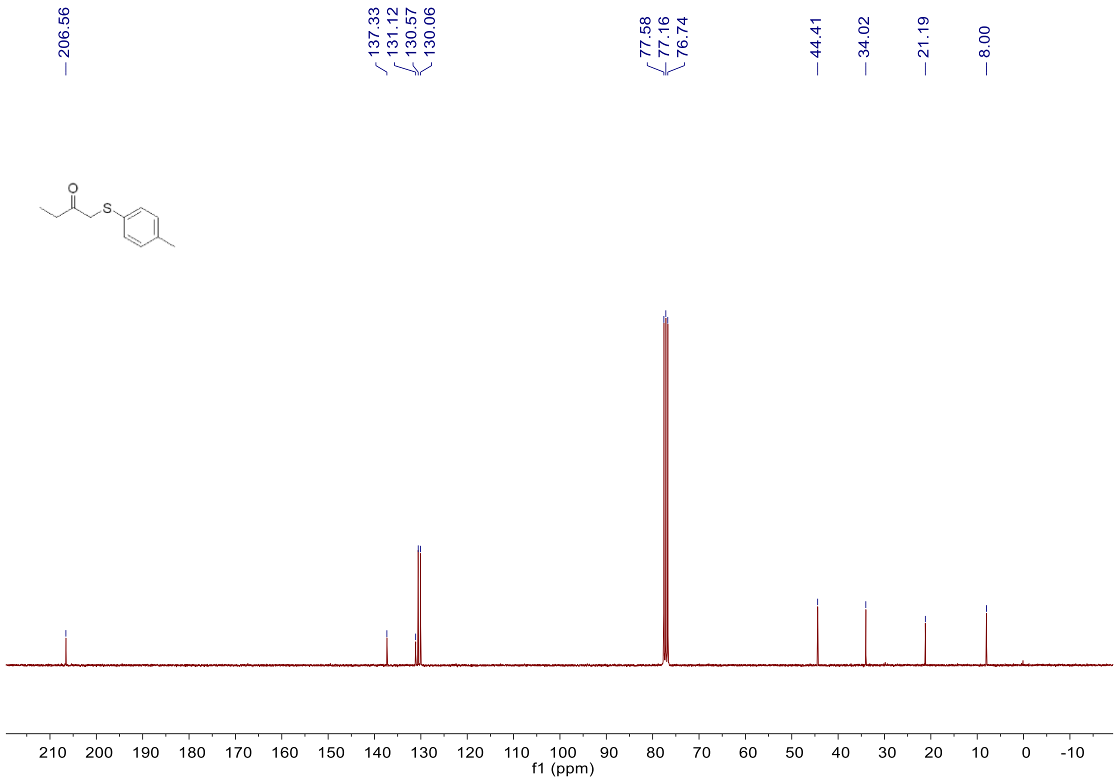

^{13}C NMR of **3-1l**

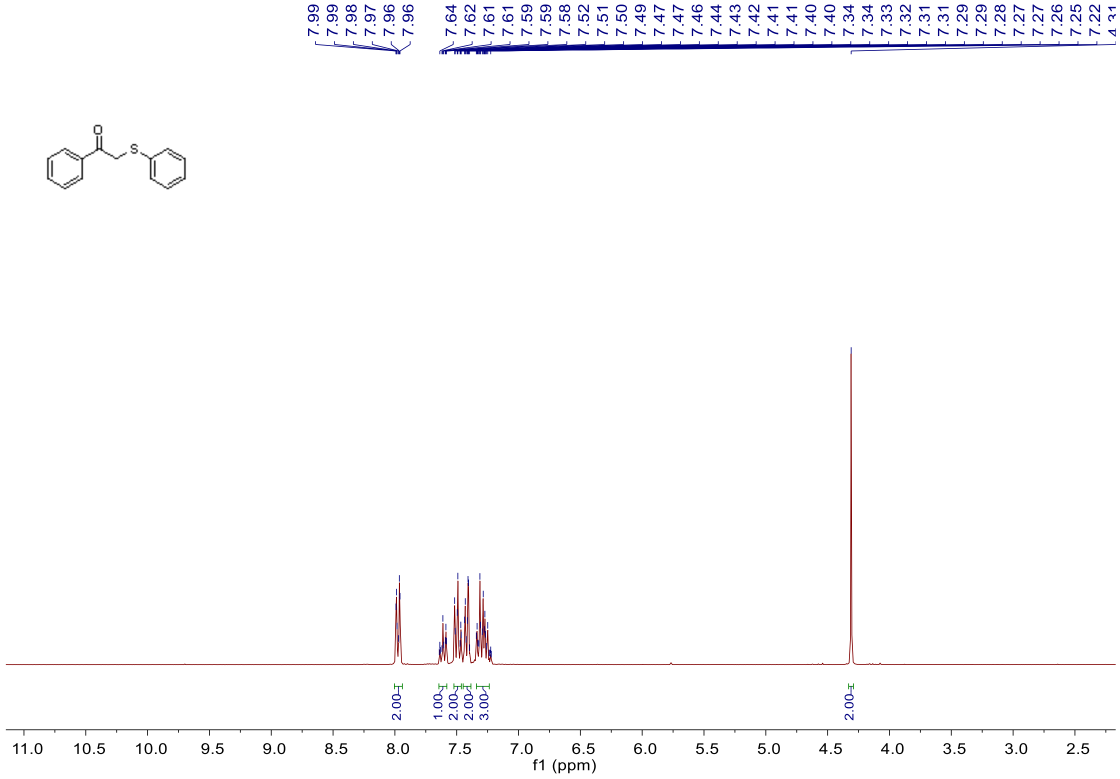

^{1}H NMR of **3-2a**

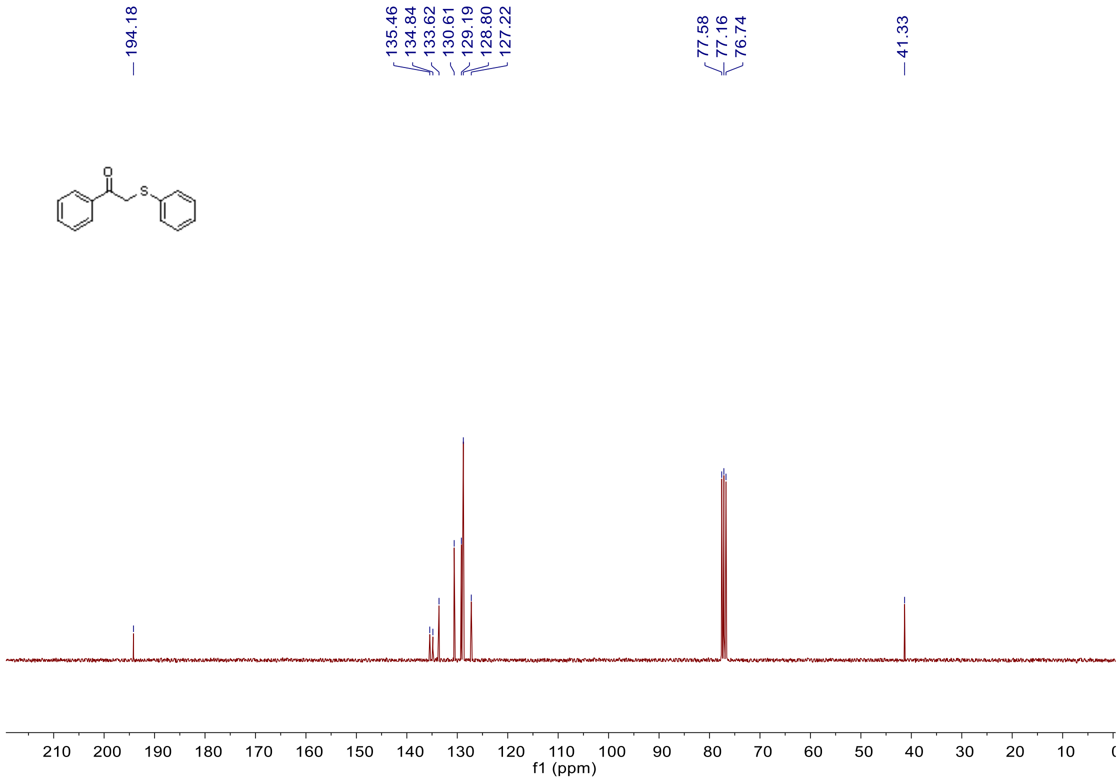

^{13}C NMR of **3-2a**

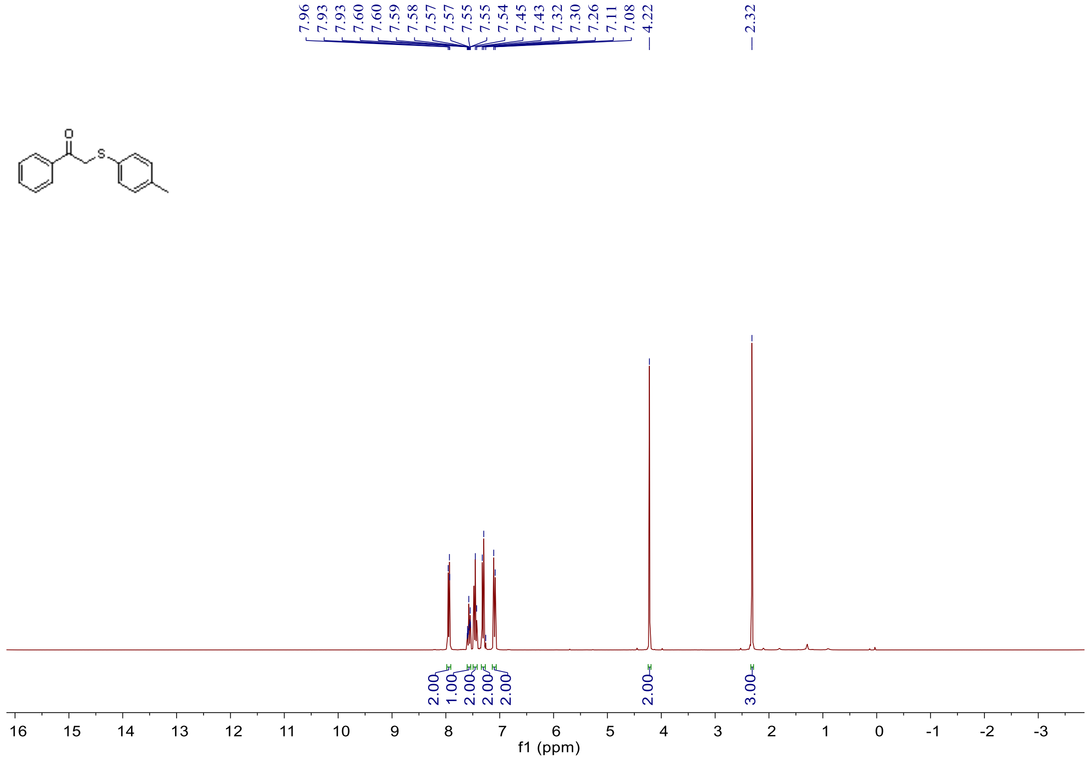

^{1}H NMR of **3-2b**

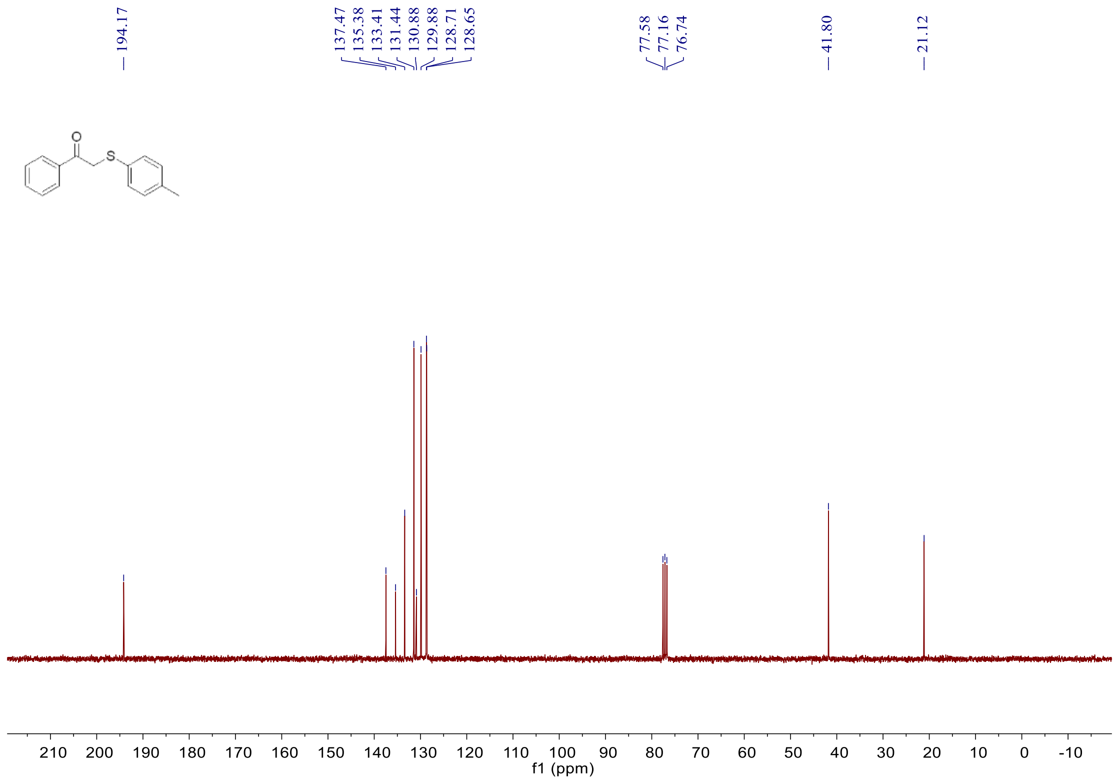

^{13}C NMR of **3-2b**

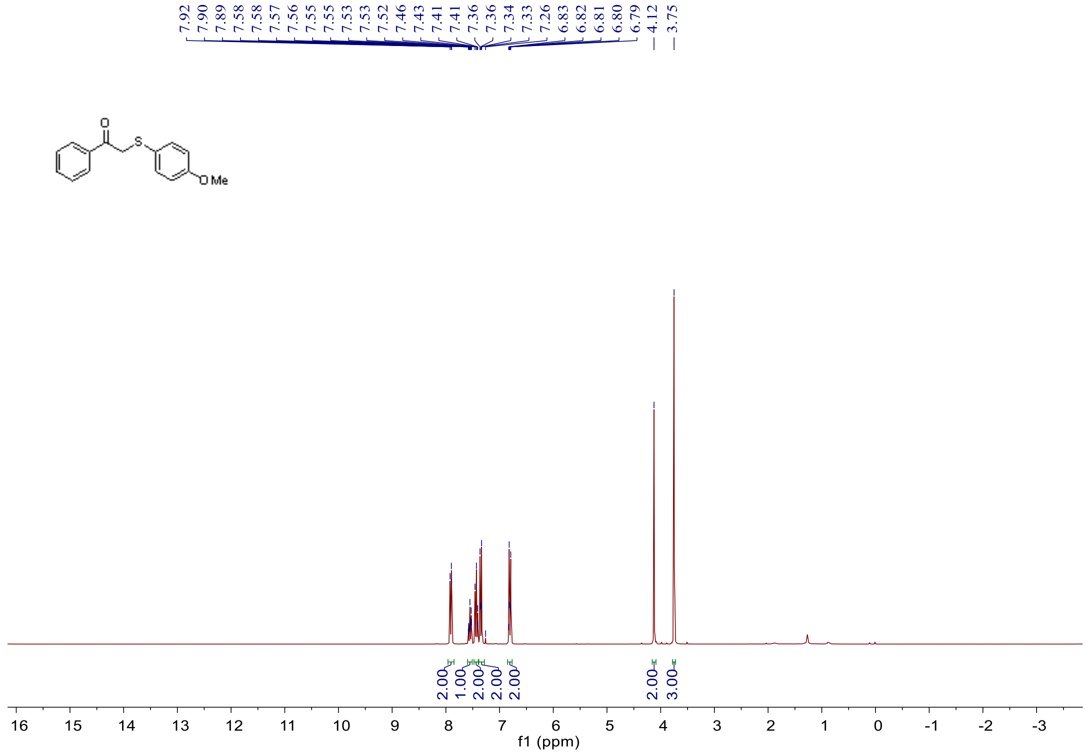

^{1}H NMR of **3-2c**

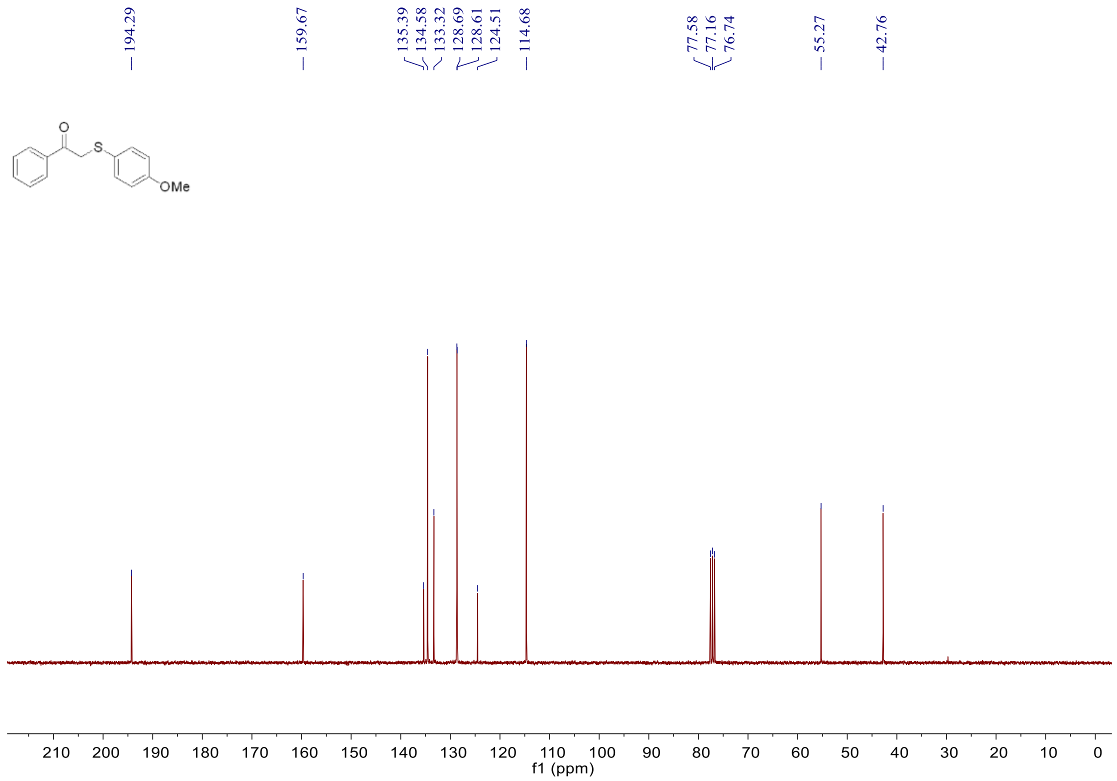

^{13}C NMR of **3-2c**

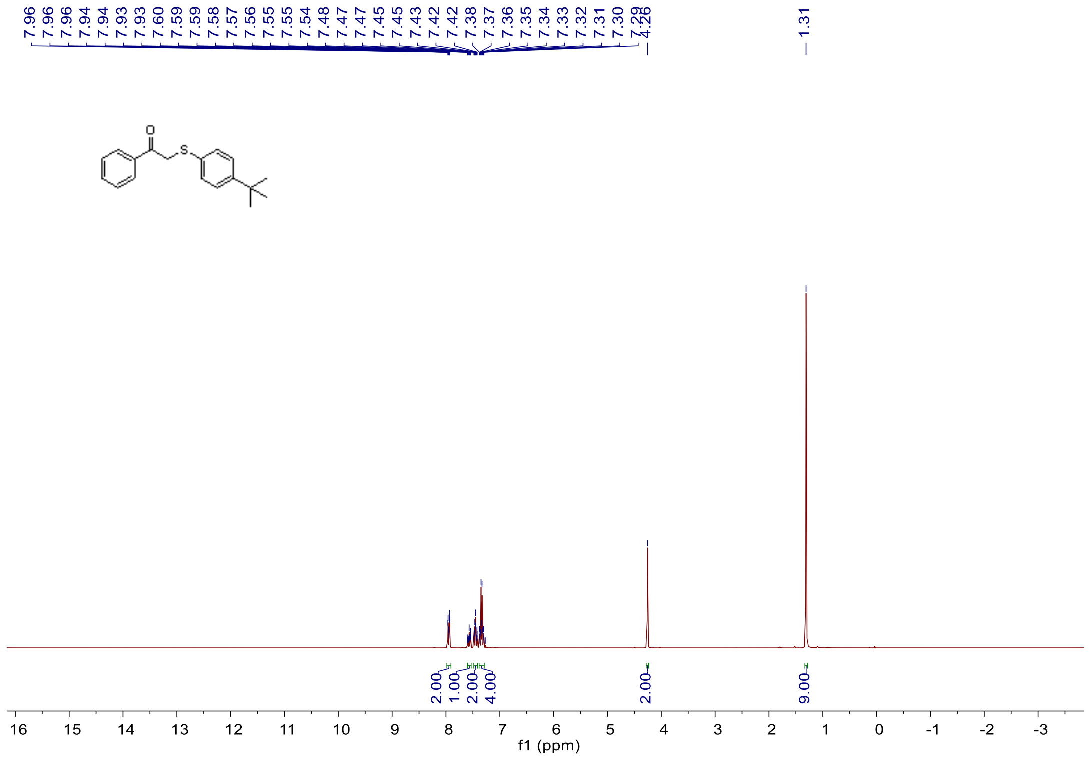

^{1}H NMR of **3-2d**

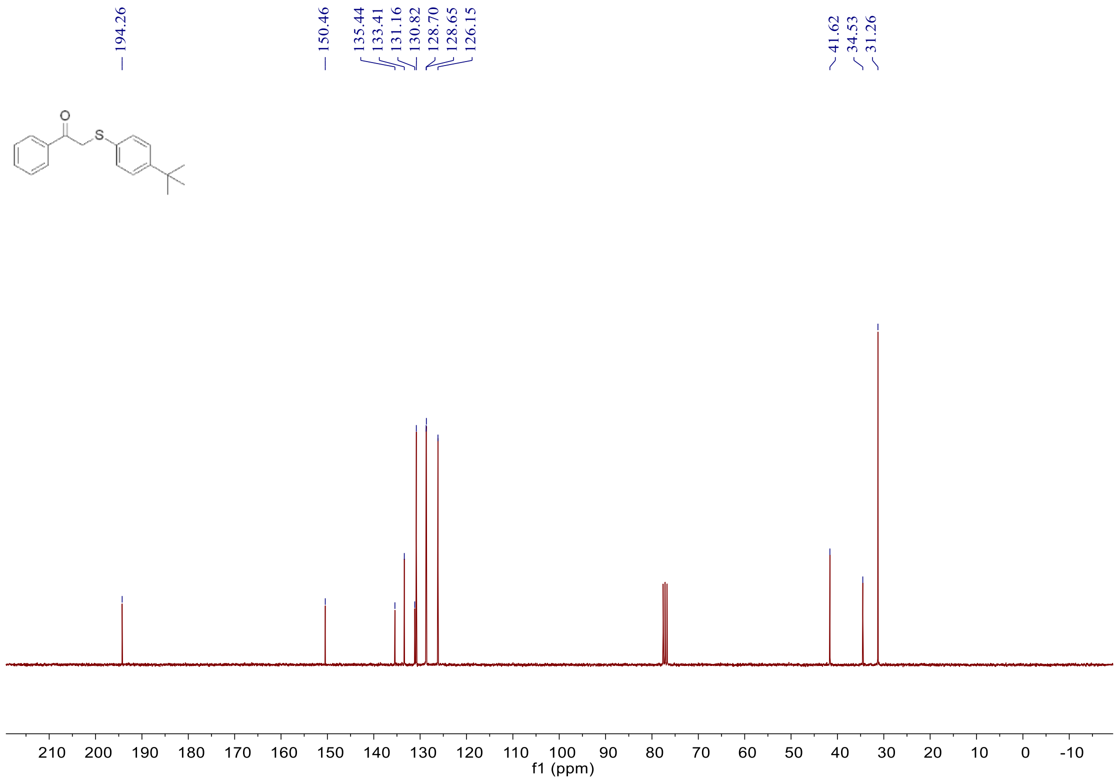

^{13}C NMR of **3-2d**

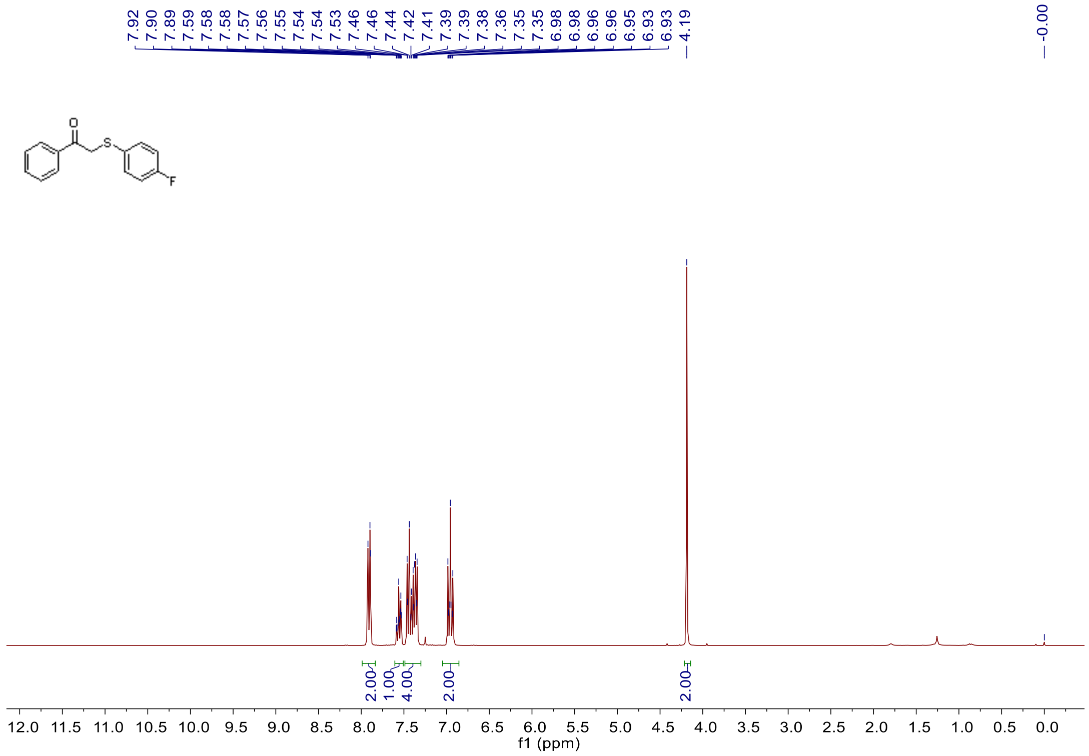

^{1}H NMR of **3-2e**

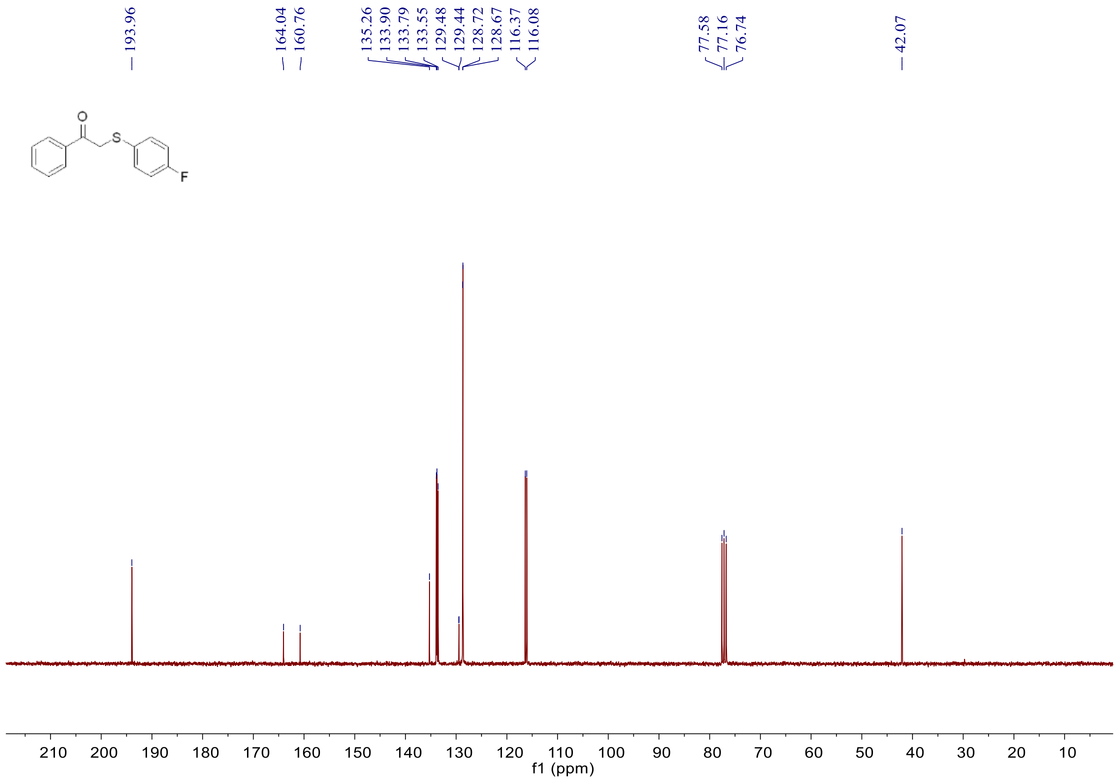

^{13}C NMR of **3-2e**

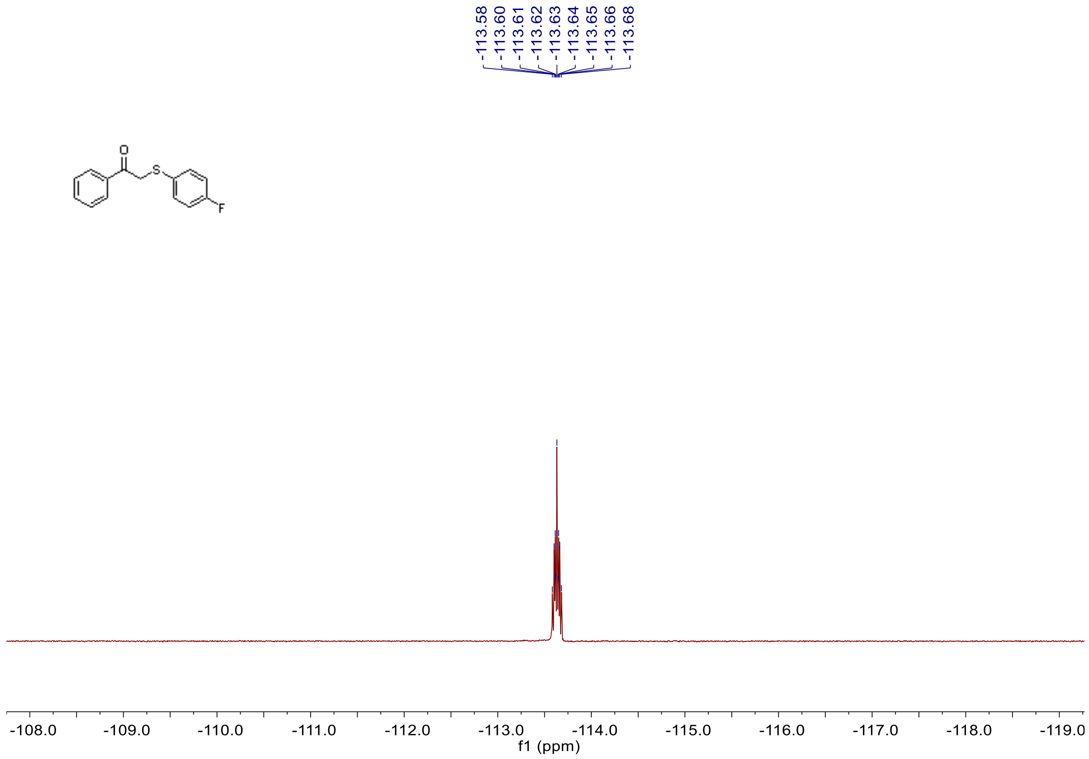

^{19}F NMR of **3-2e**

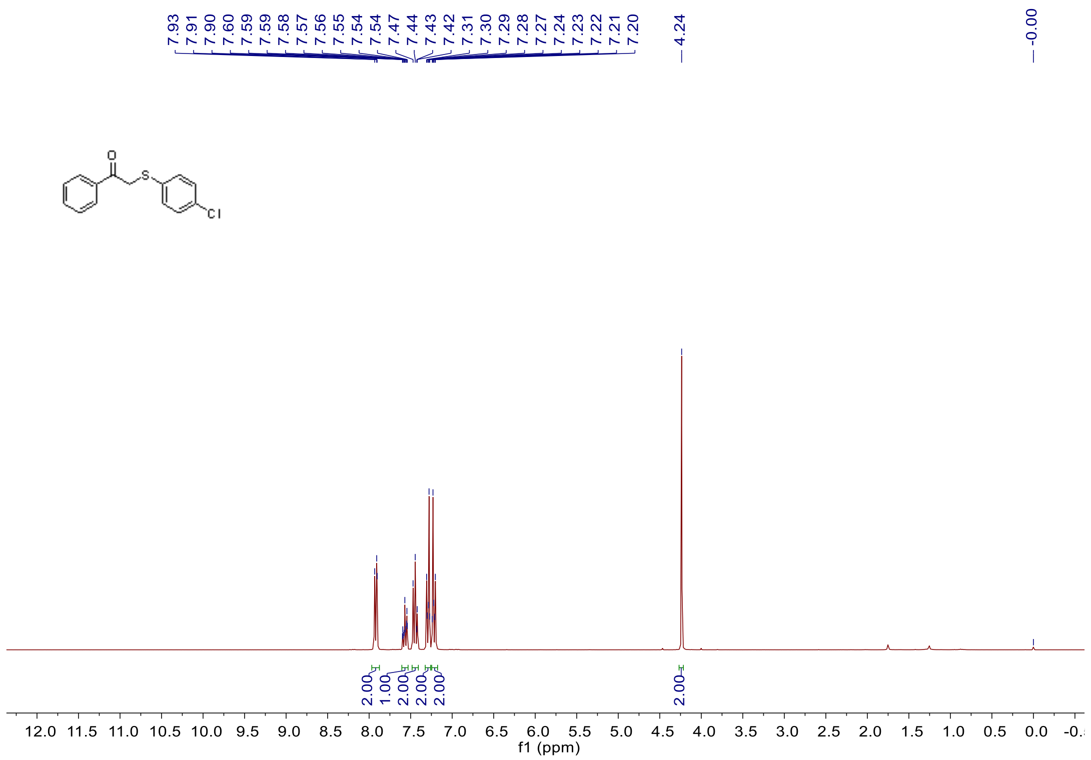

^{1}H NMR of **3-2f**

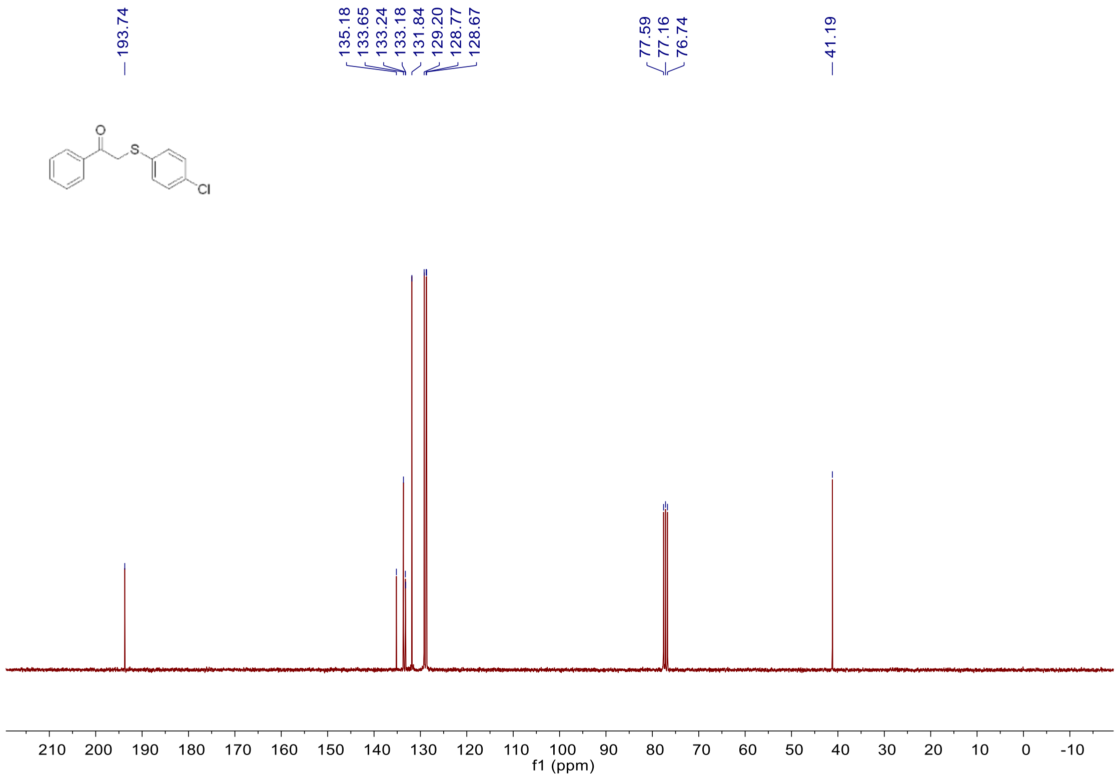

^{13}C NMR of **3-2f**

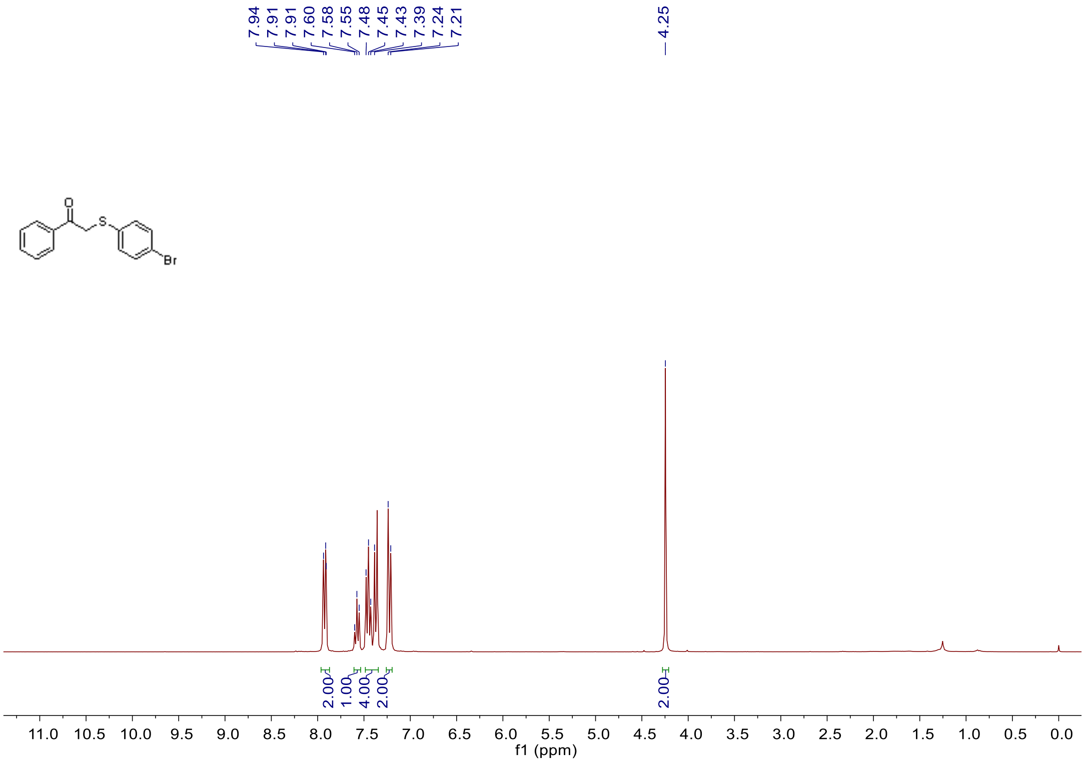

^{1}H NMR of **3-2g**

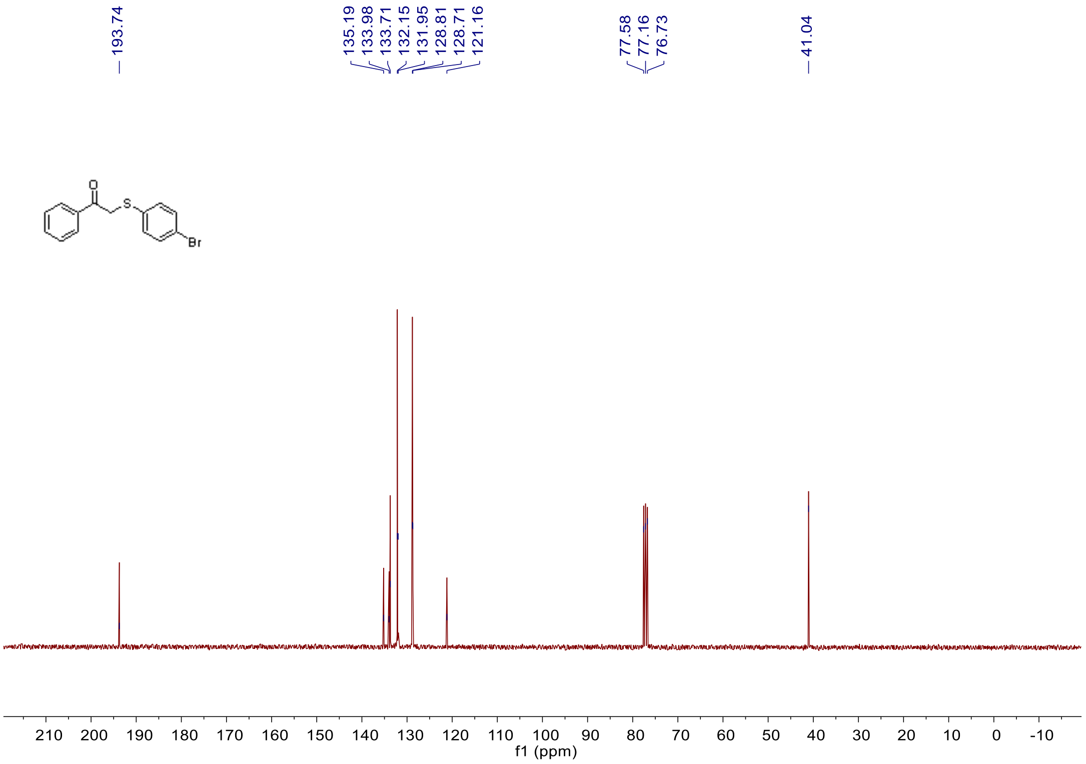

^{13}C NMR of **3-2g**

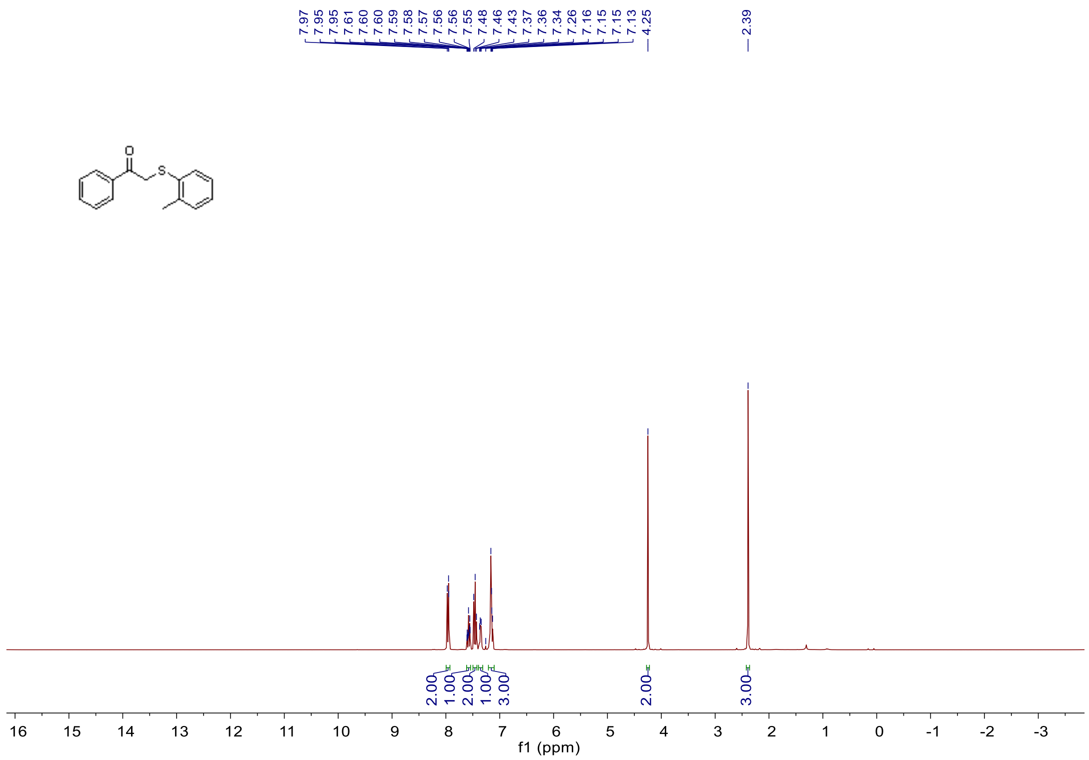

^{1}H NMR of **3-2h**

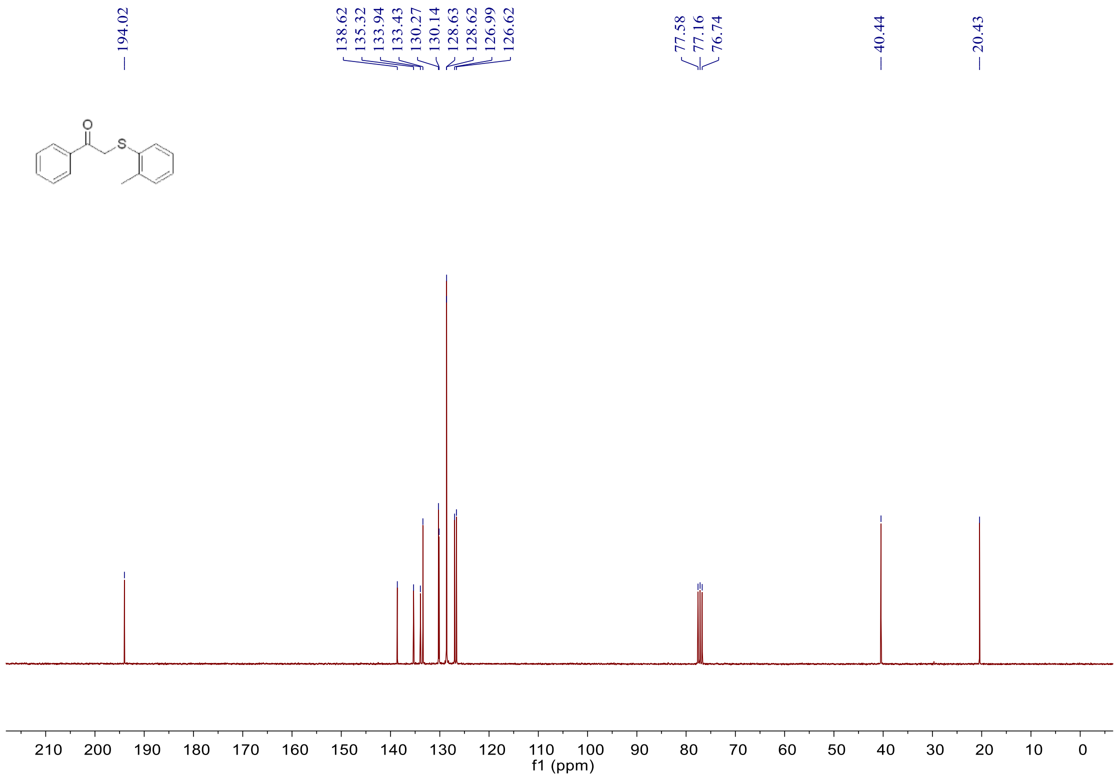

^{13}C NMR of **3-2h**

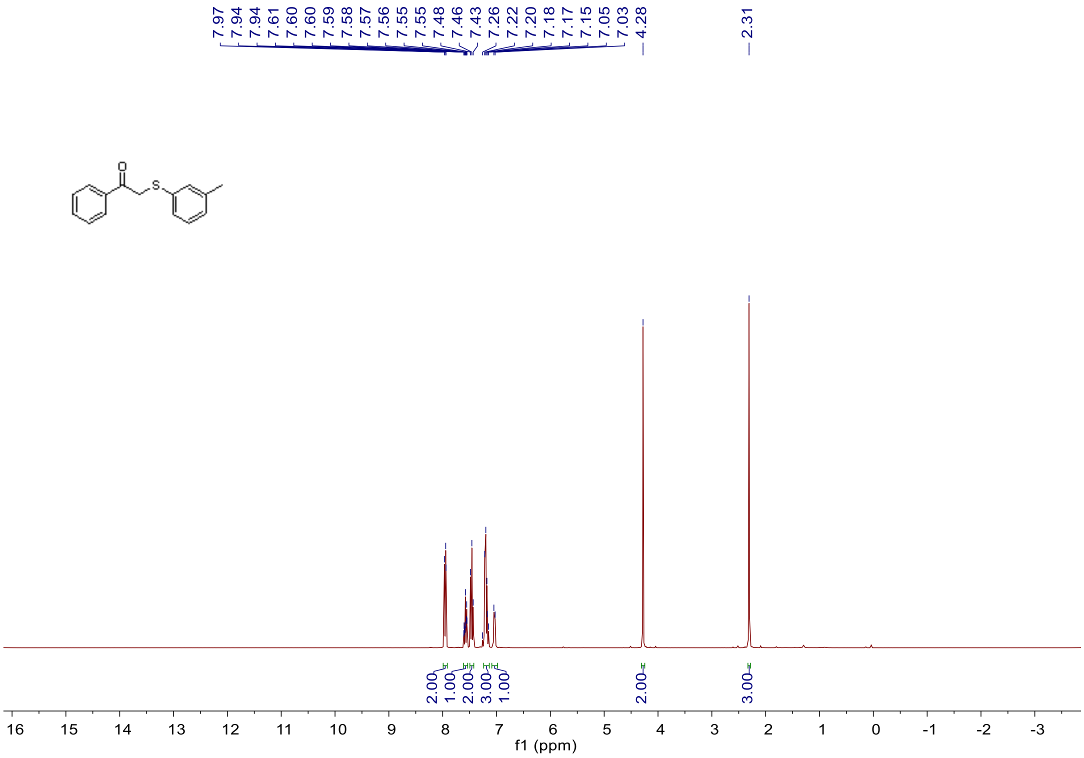

^{1}H NMR of **3-2i**

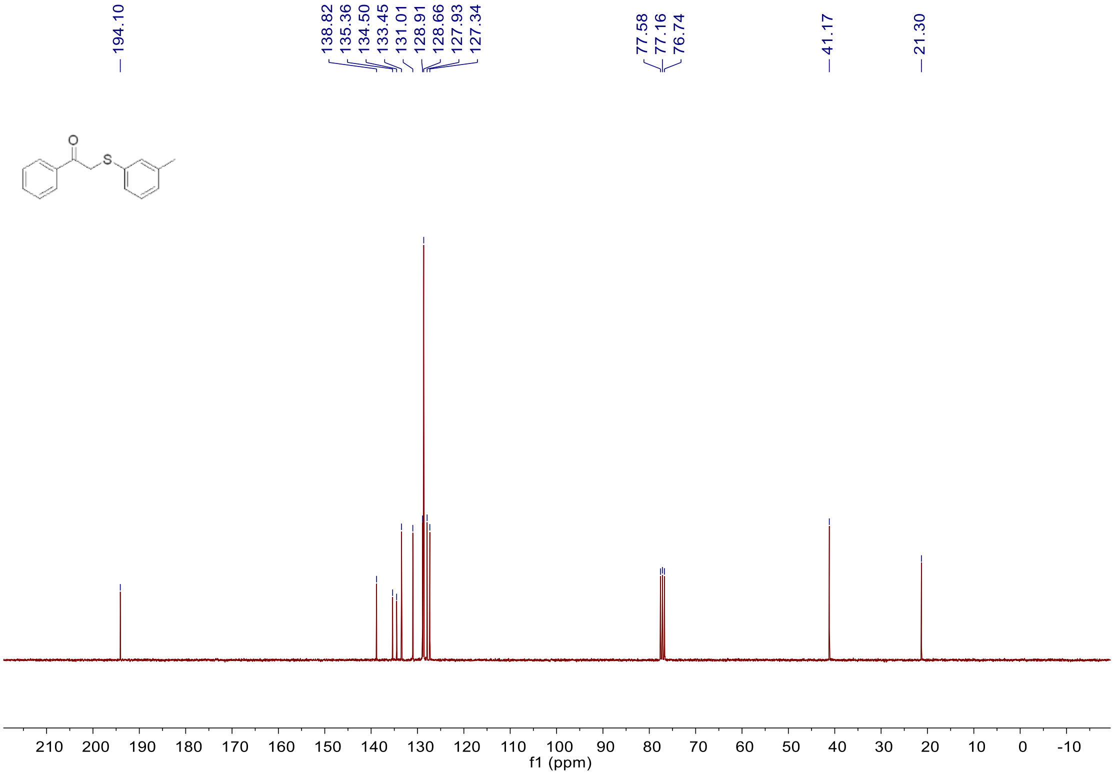

^{13}C NMR of **3-2i**

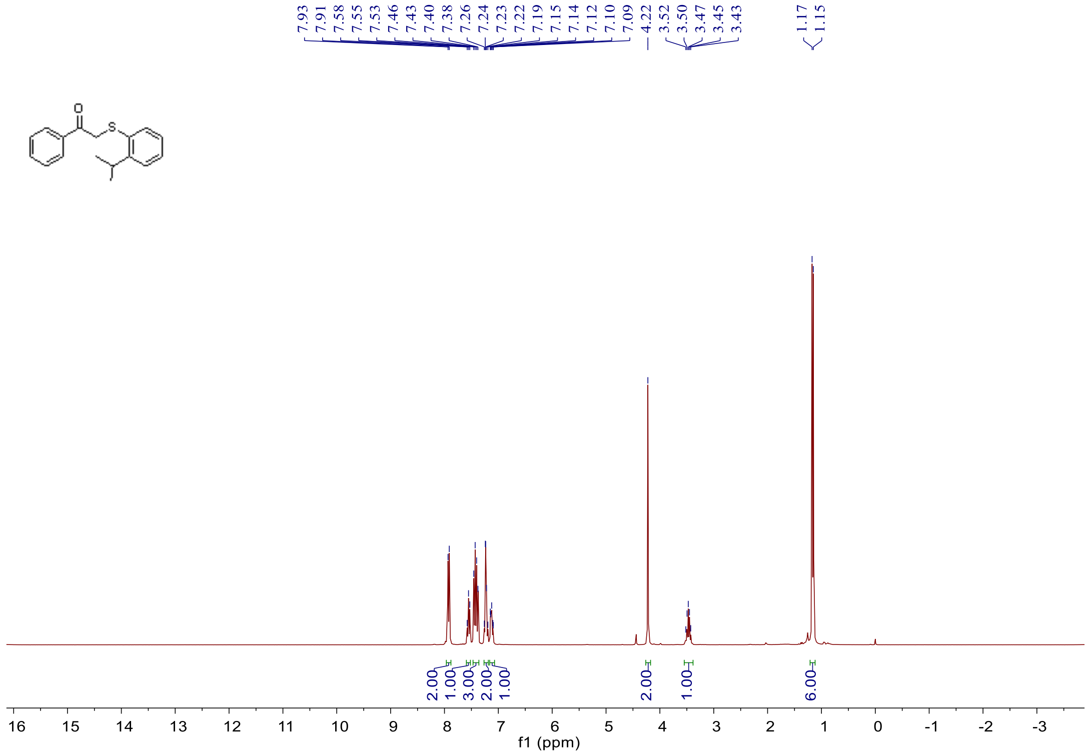

^{1}H NMR of **3-2j**

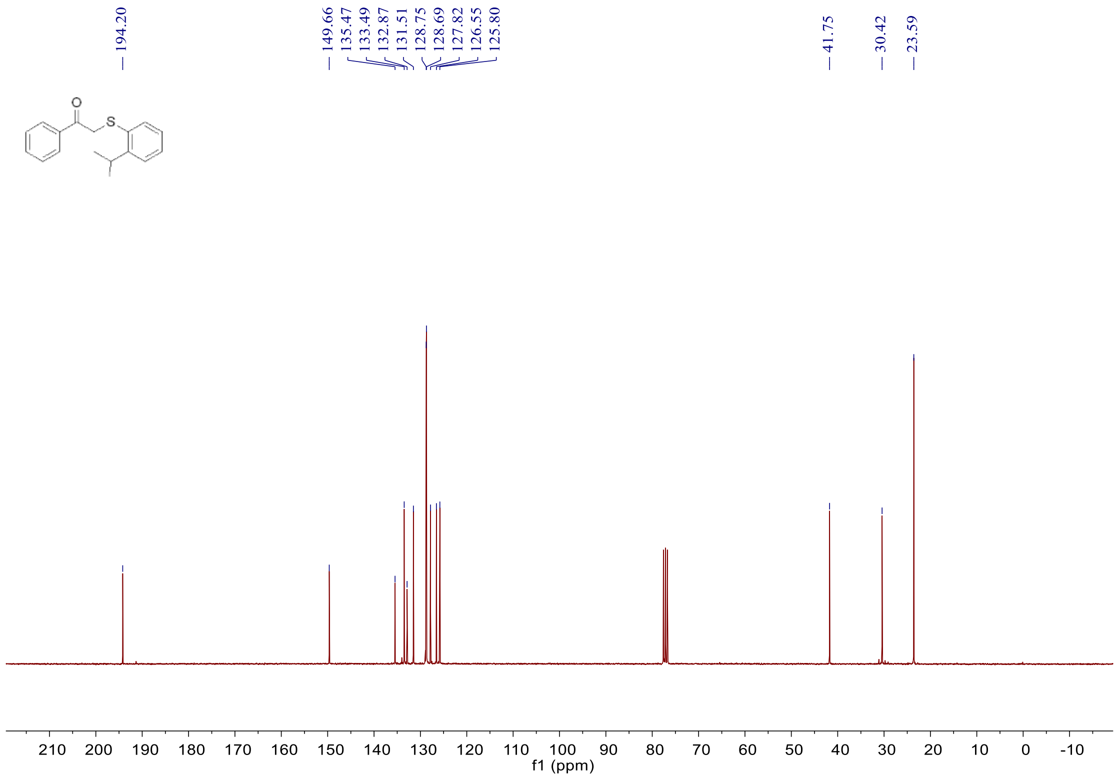

^{13}C NMR of **3-2j**

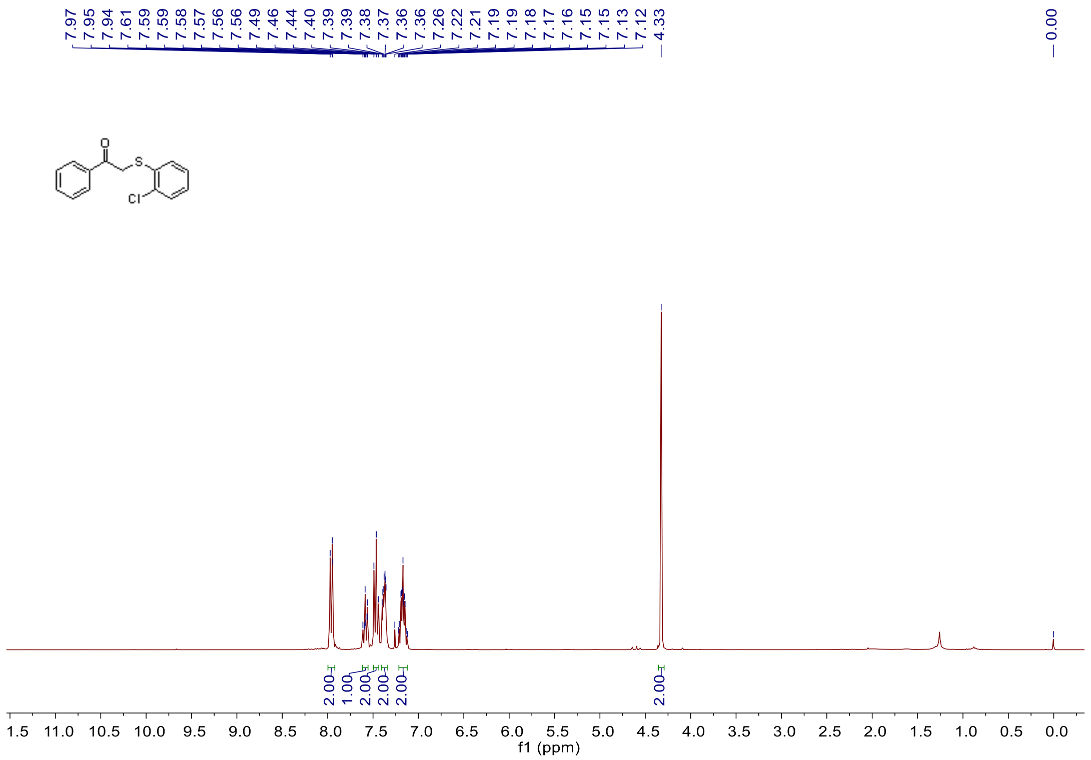

^{1}H NMR of **3-2k**

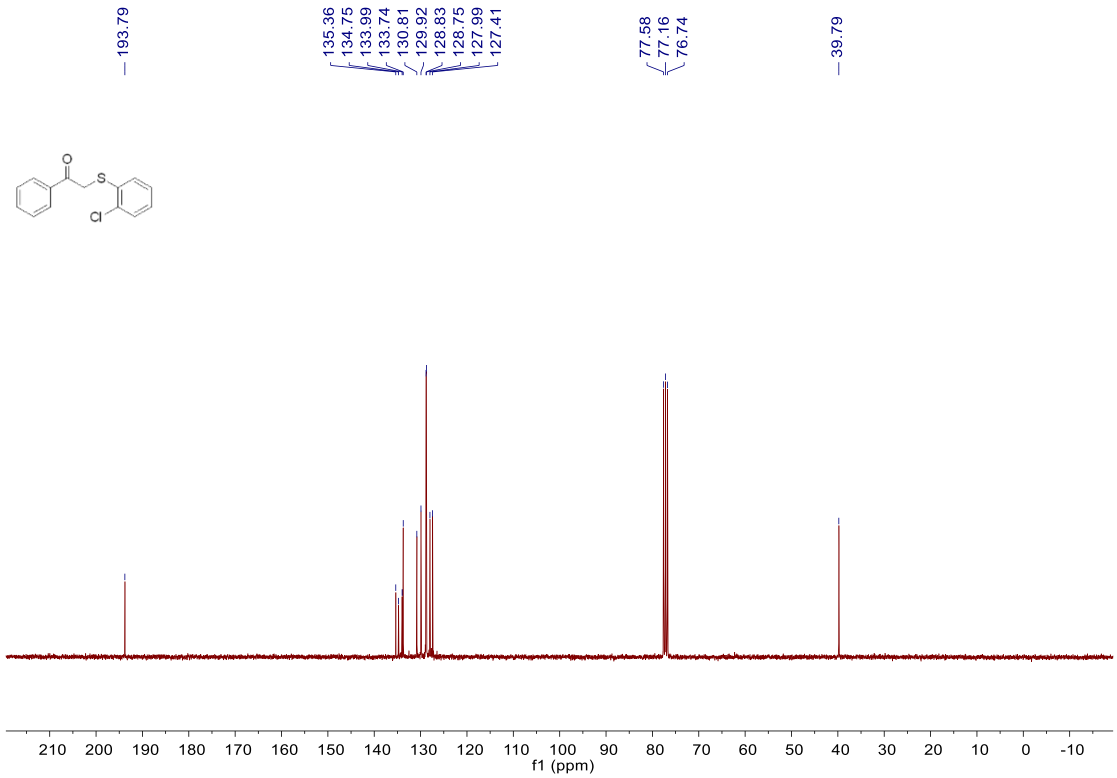

^{13}C NMR of **3-2k**

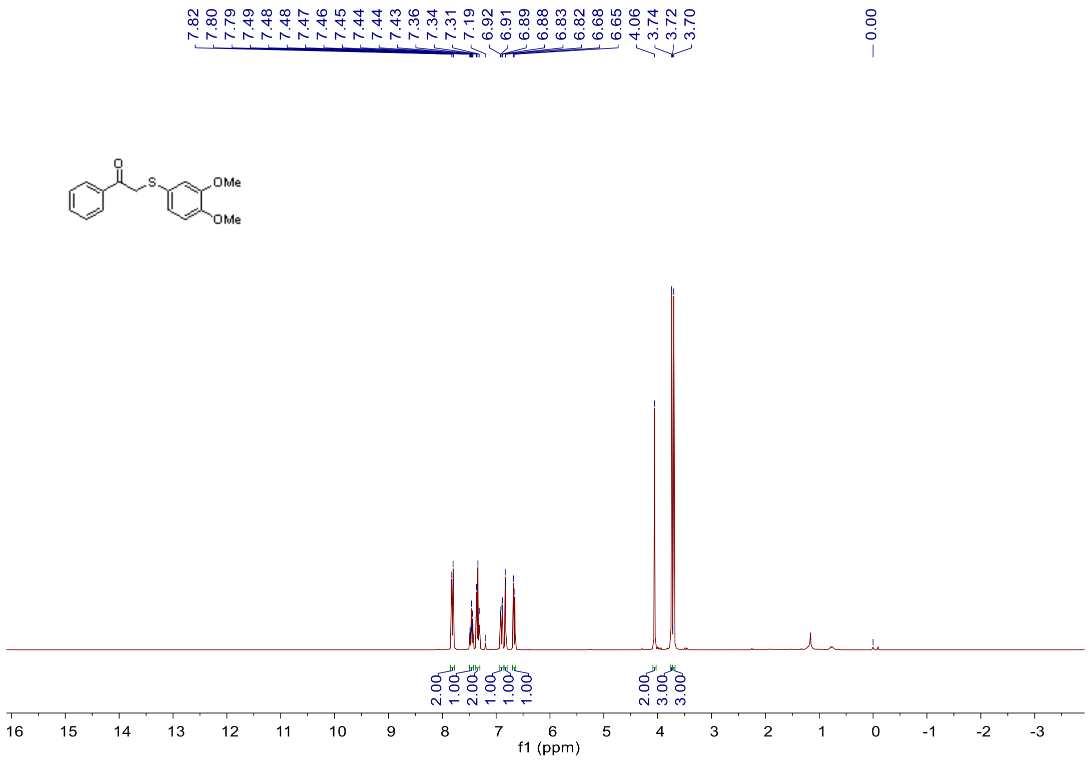

^{1}H NMR of **3-2l**

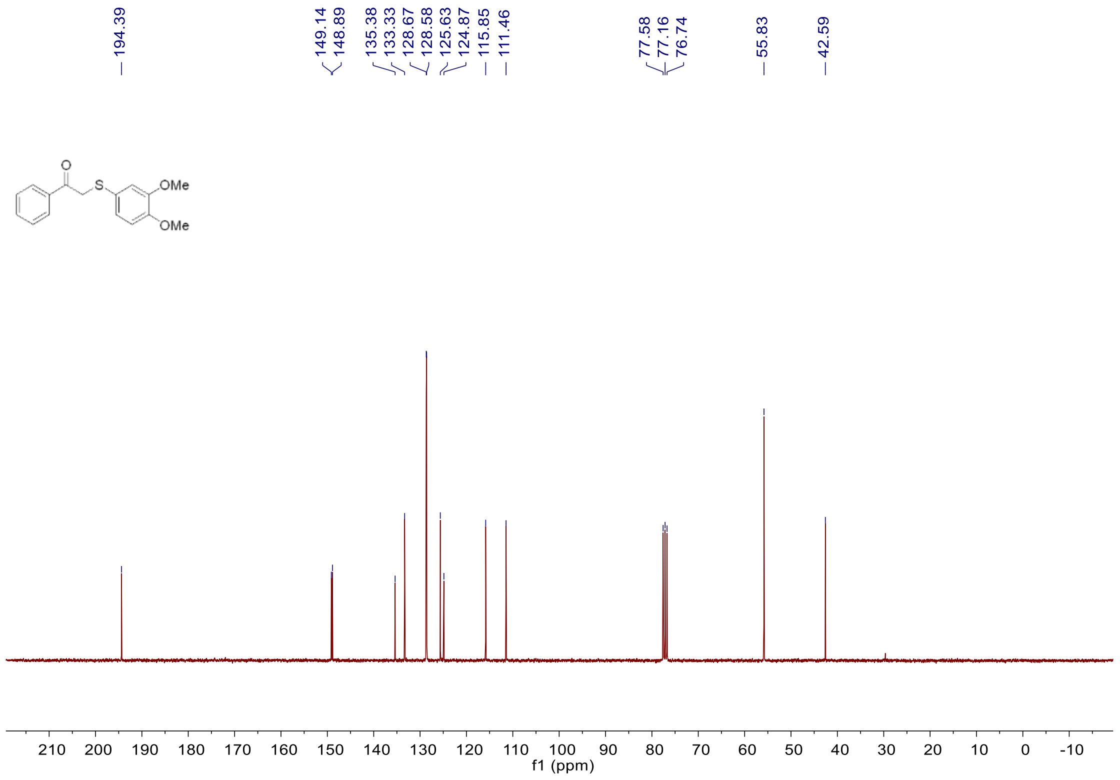

^{13}C NMR of **3-2l**

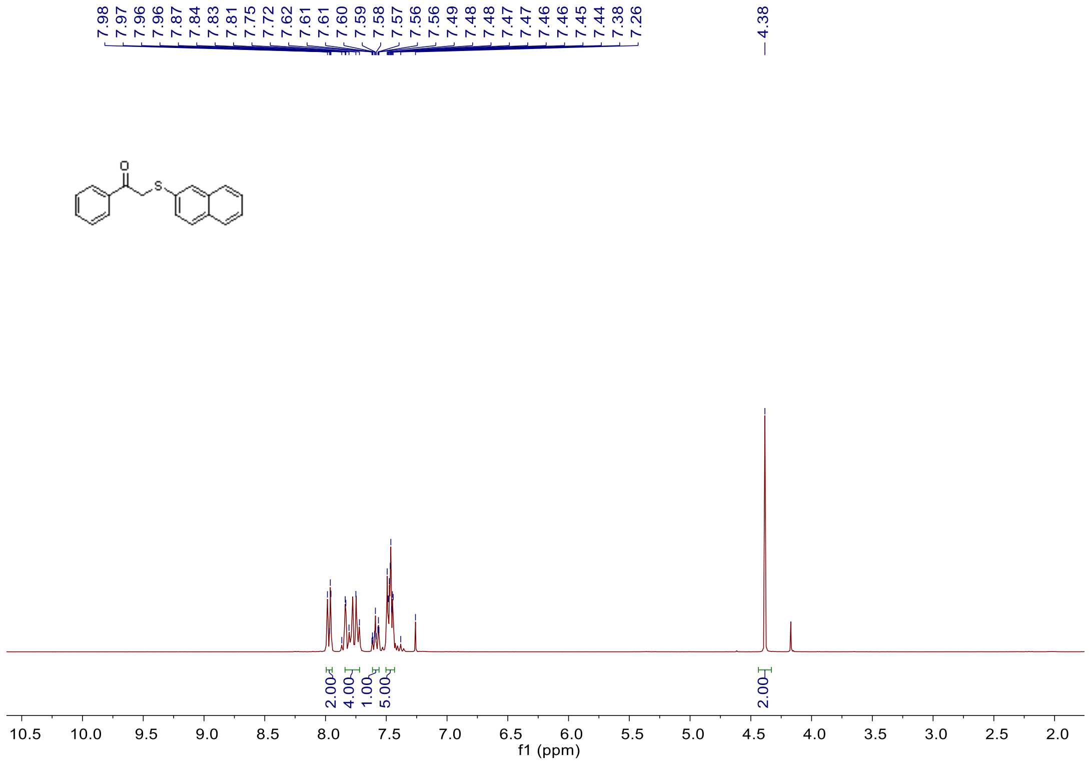

^{1}H NMR of **3-2m**

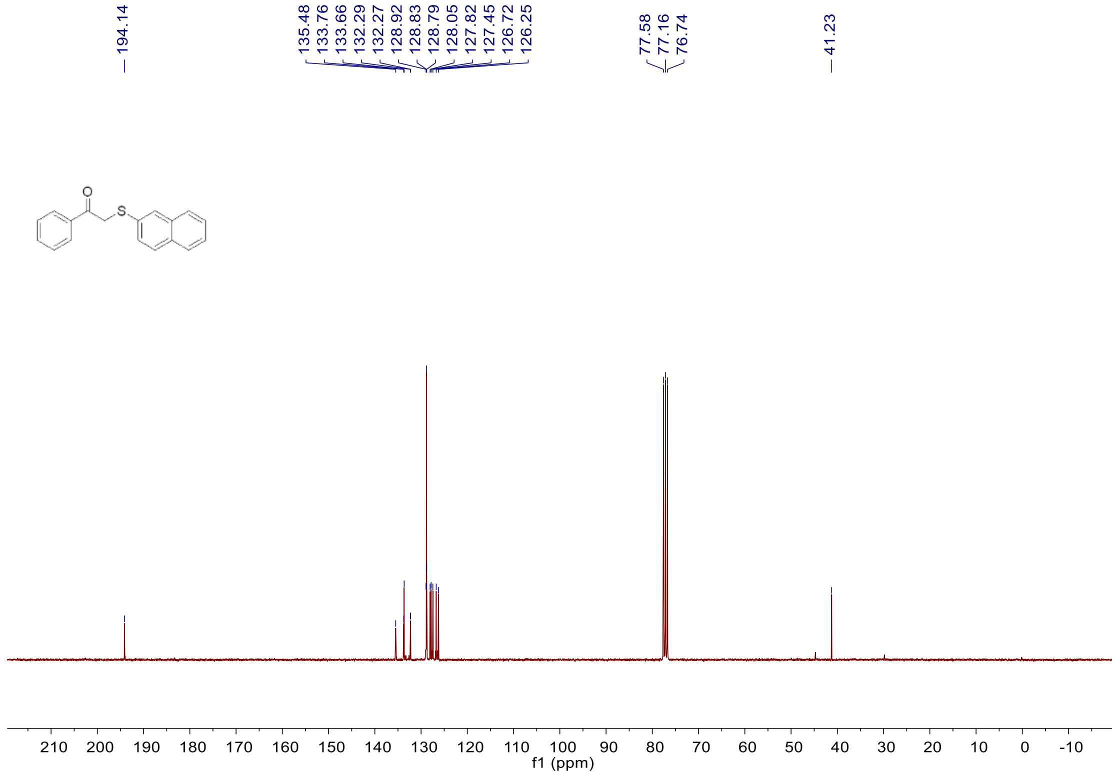

^{13}C NMR of **3-2m**

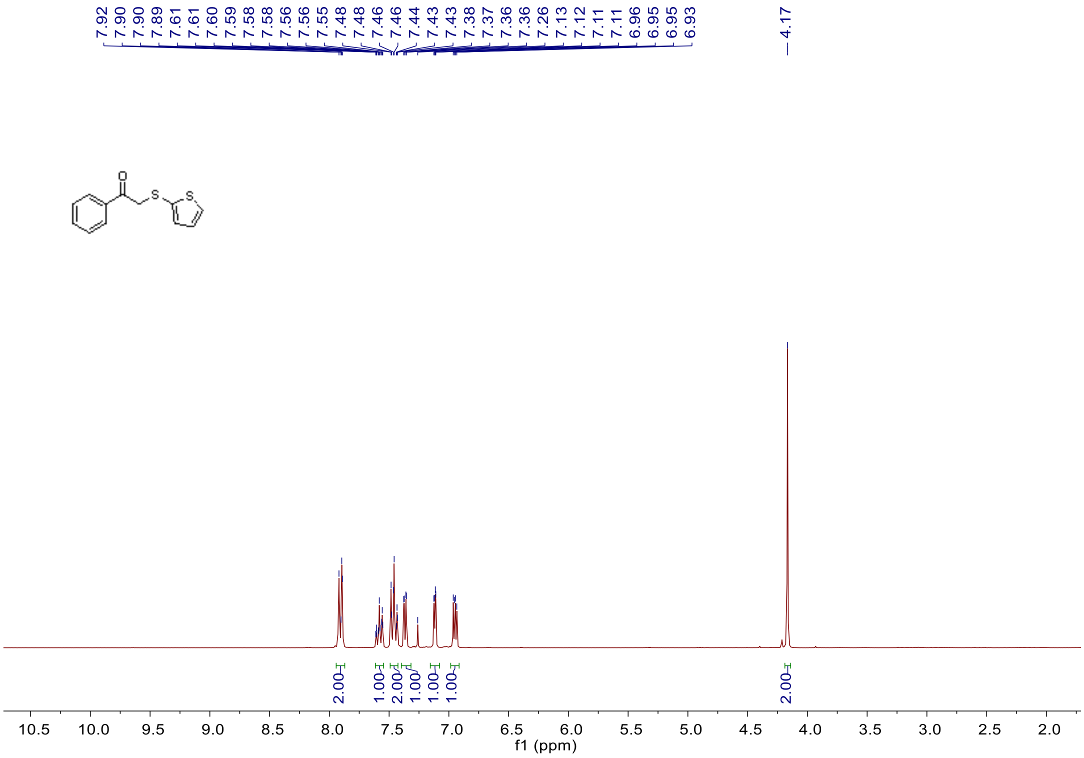

^{1}H NMR of **3-2n**

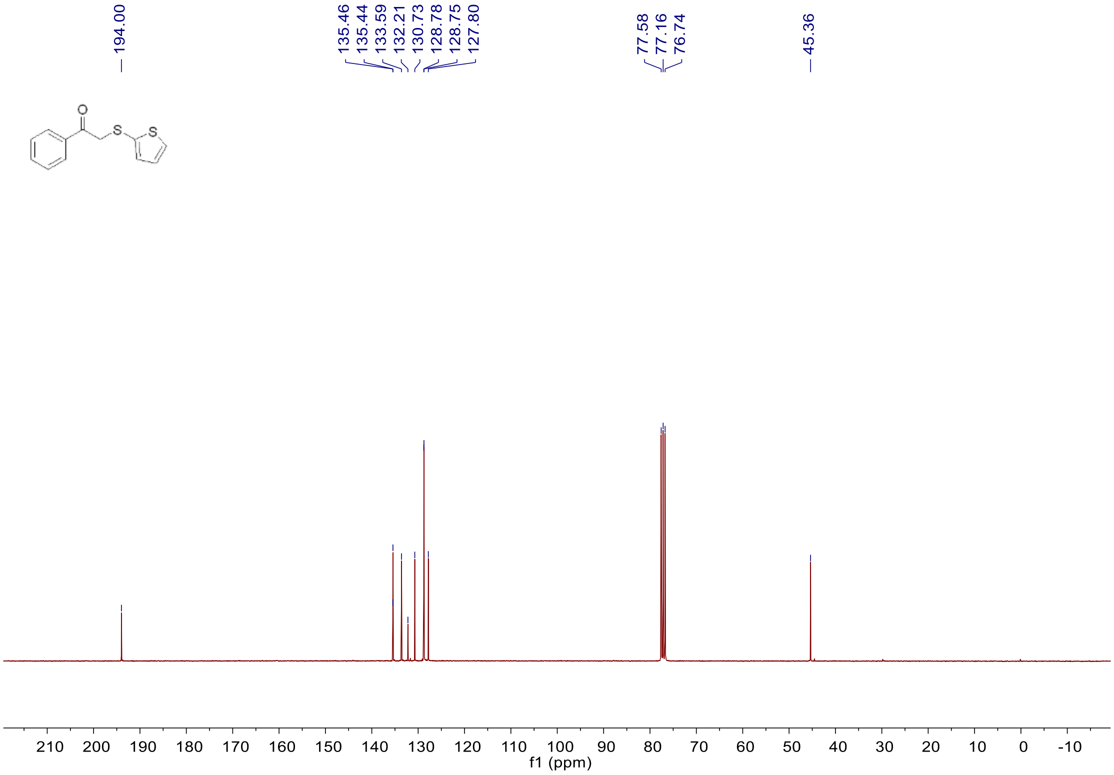

^{13}C NMR of **3-2n**

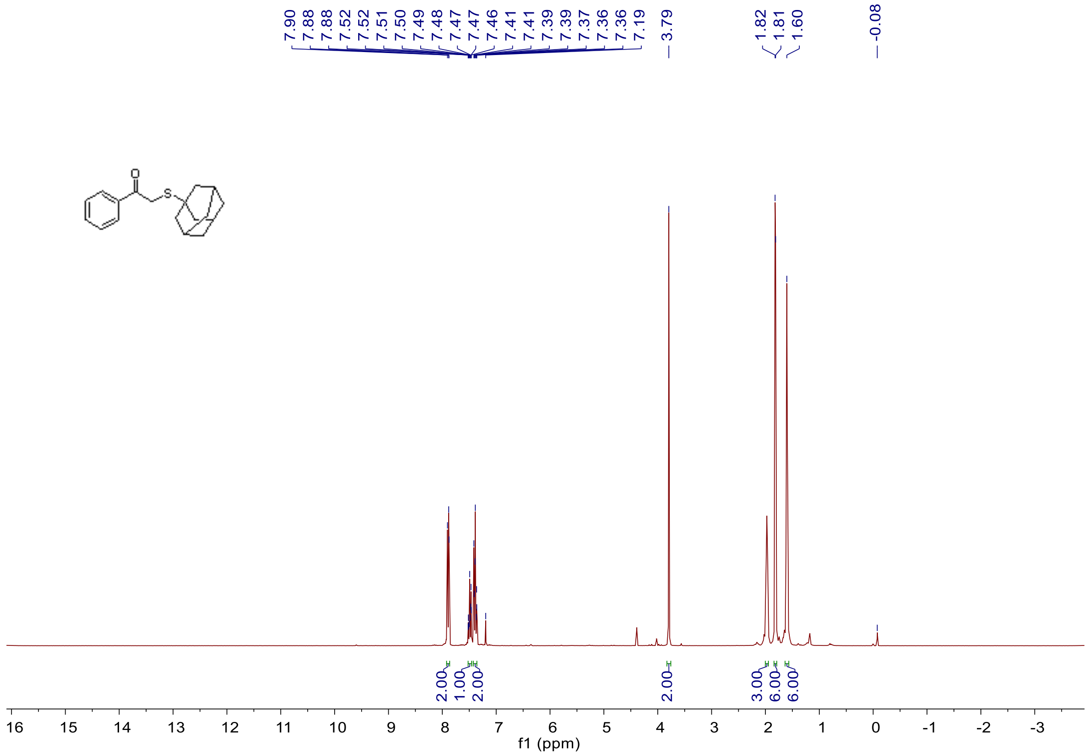

^{1}H NMR of **3-2o**

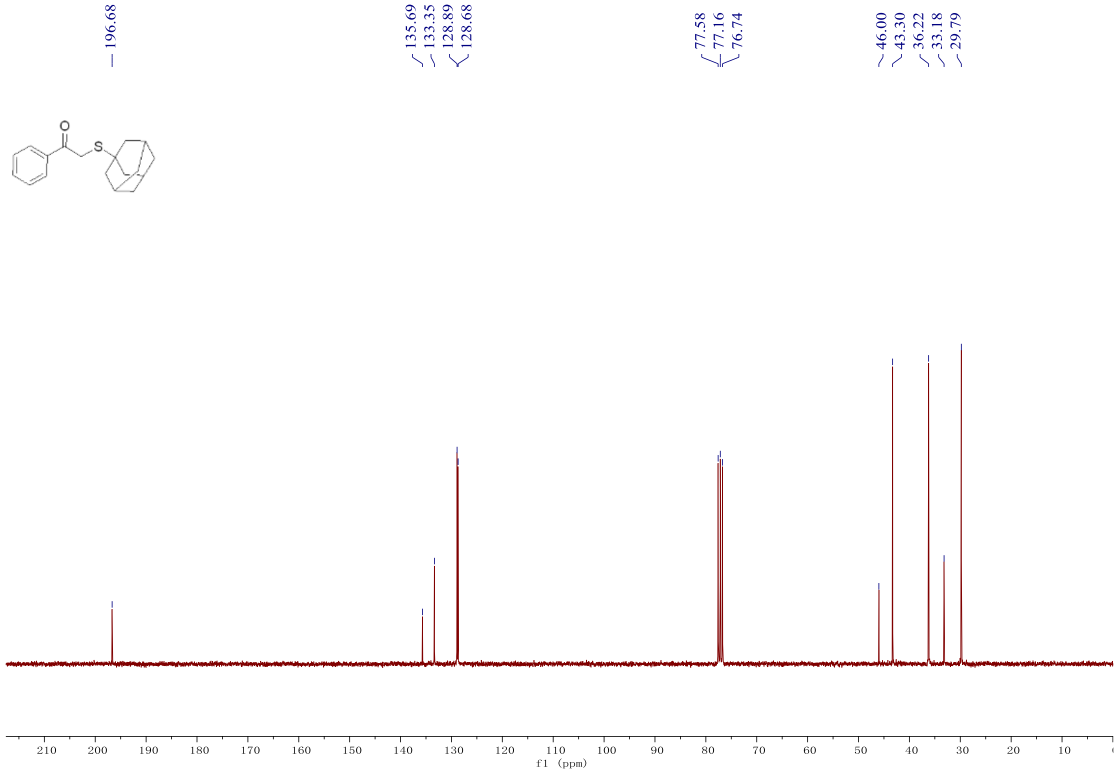

^{13}C NMR of **3-2o**

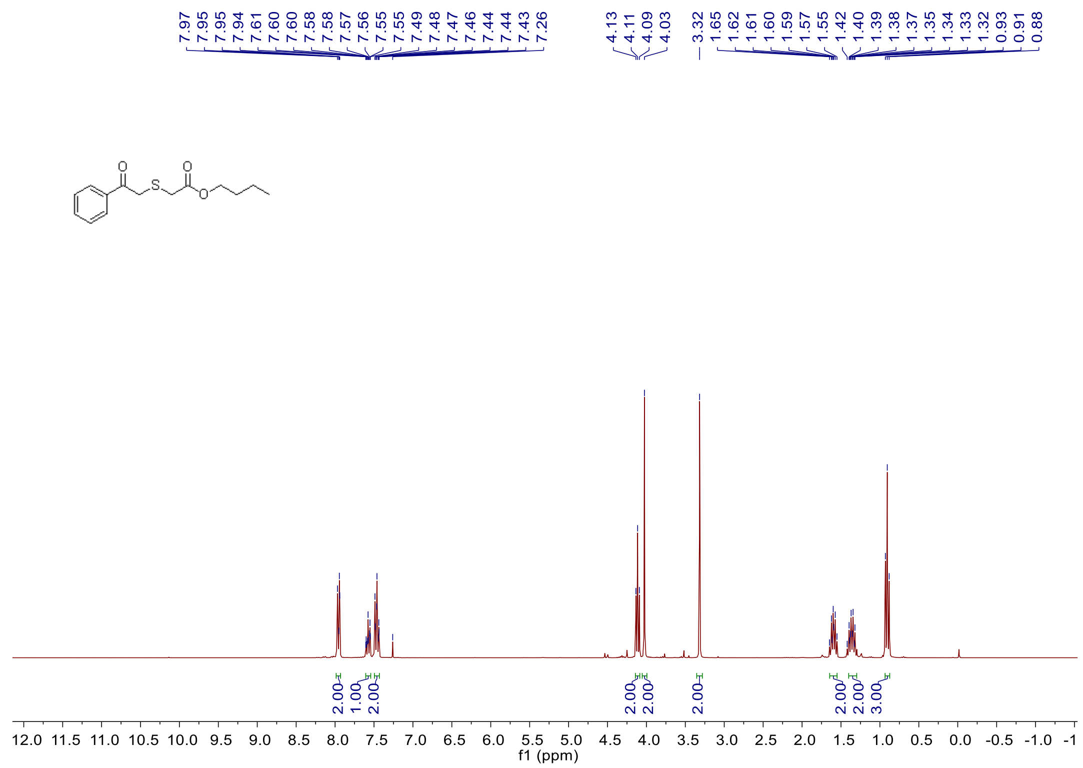

^{1}H NMR of **3-2p**

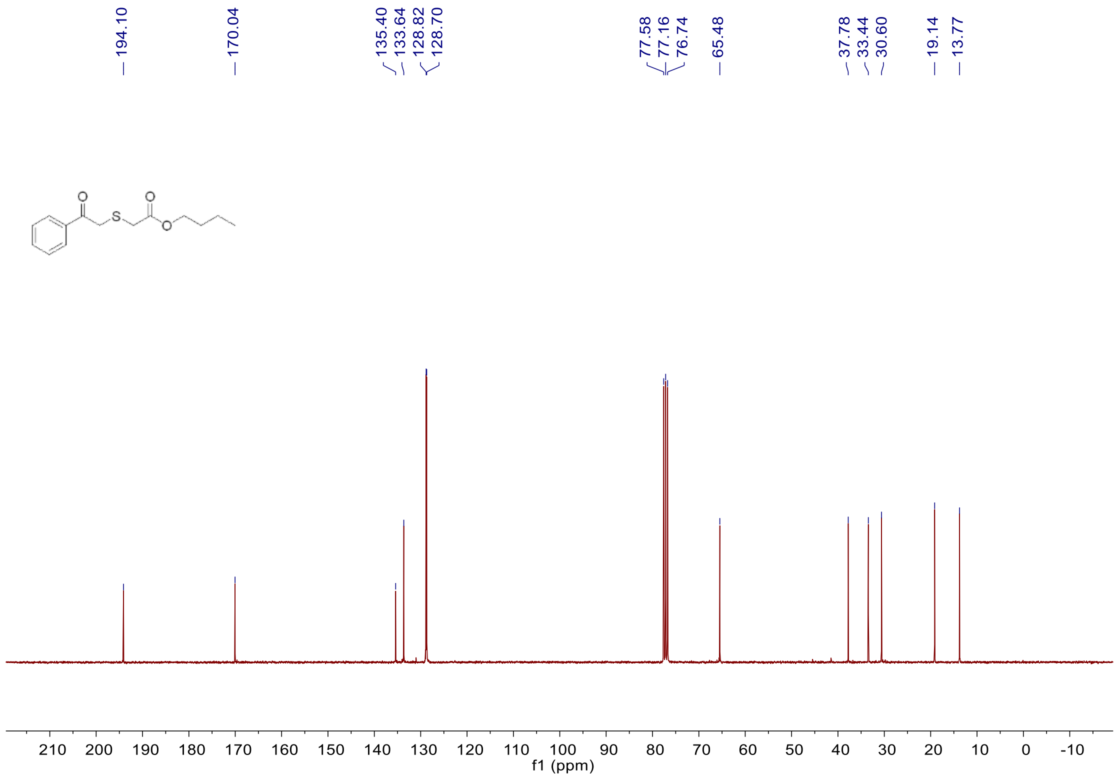

^{13}C NMR of **3-2p**

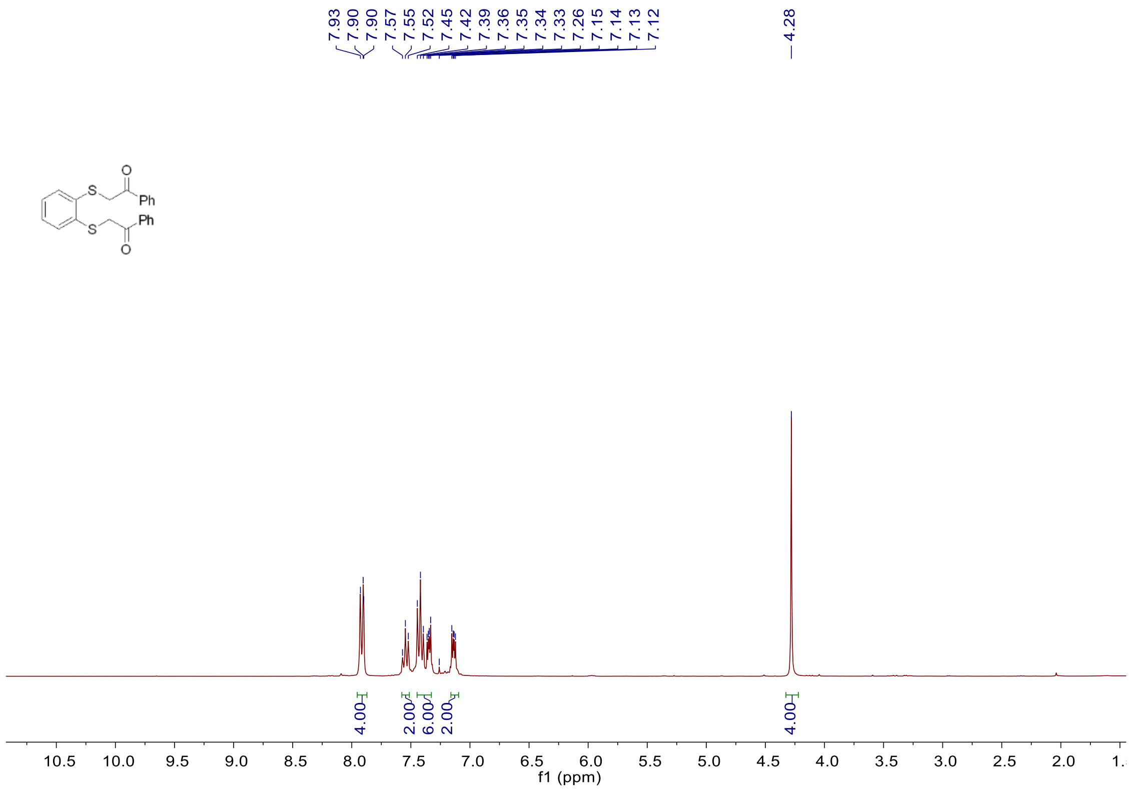

^{1}H NMR of **3-2q**

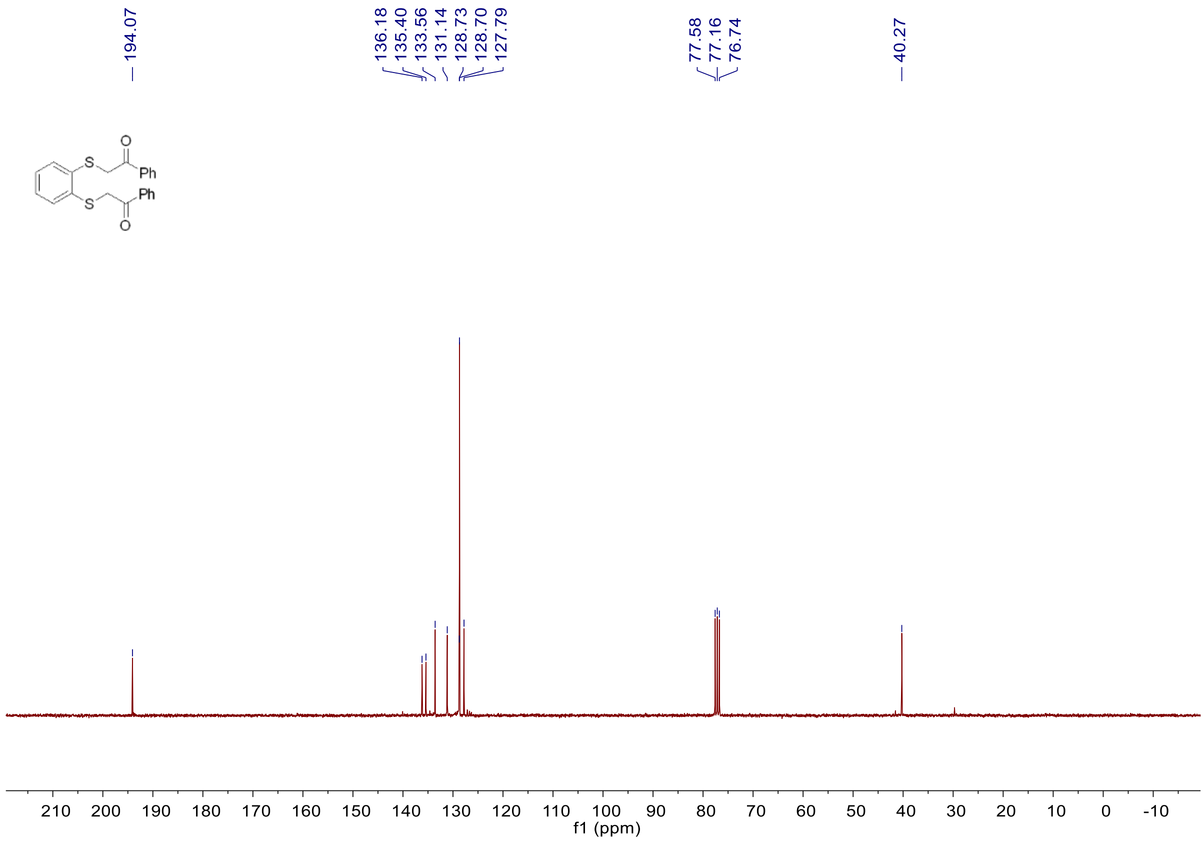

^{13}C NMR of **3-2q**

^{1}H NMR of **D**

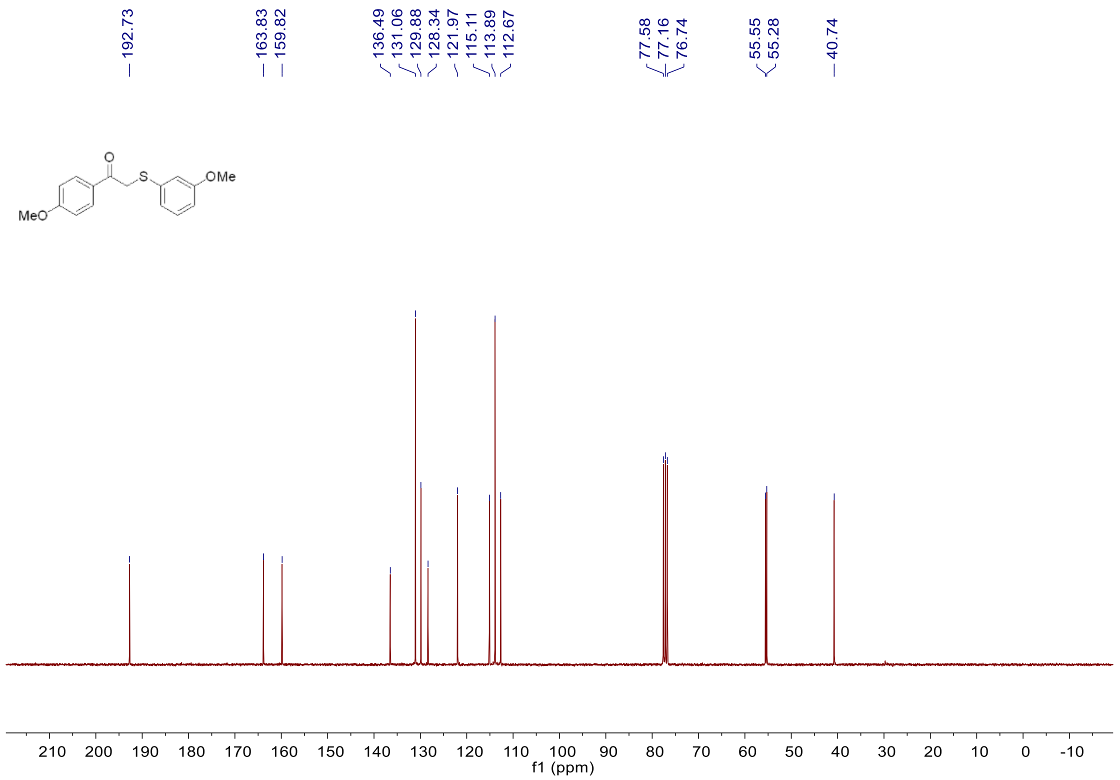

^{13}C NMR of **D**

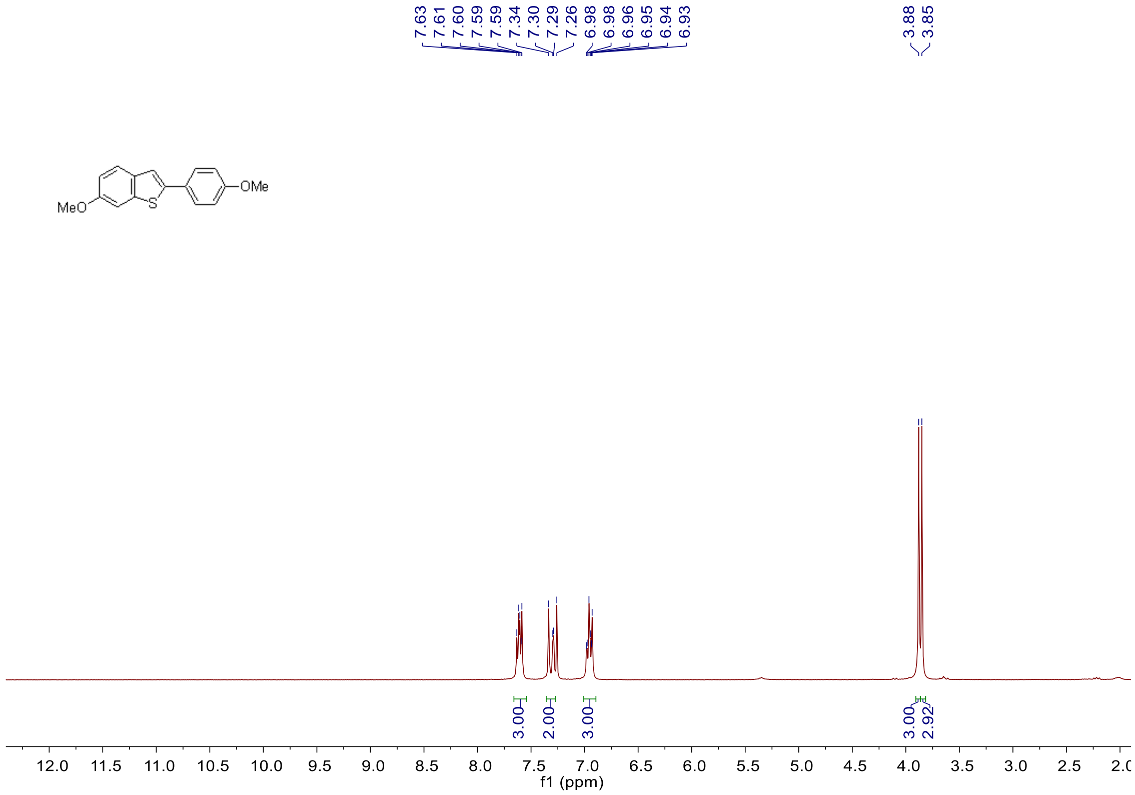

^{1}H NMR of **E**

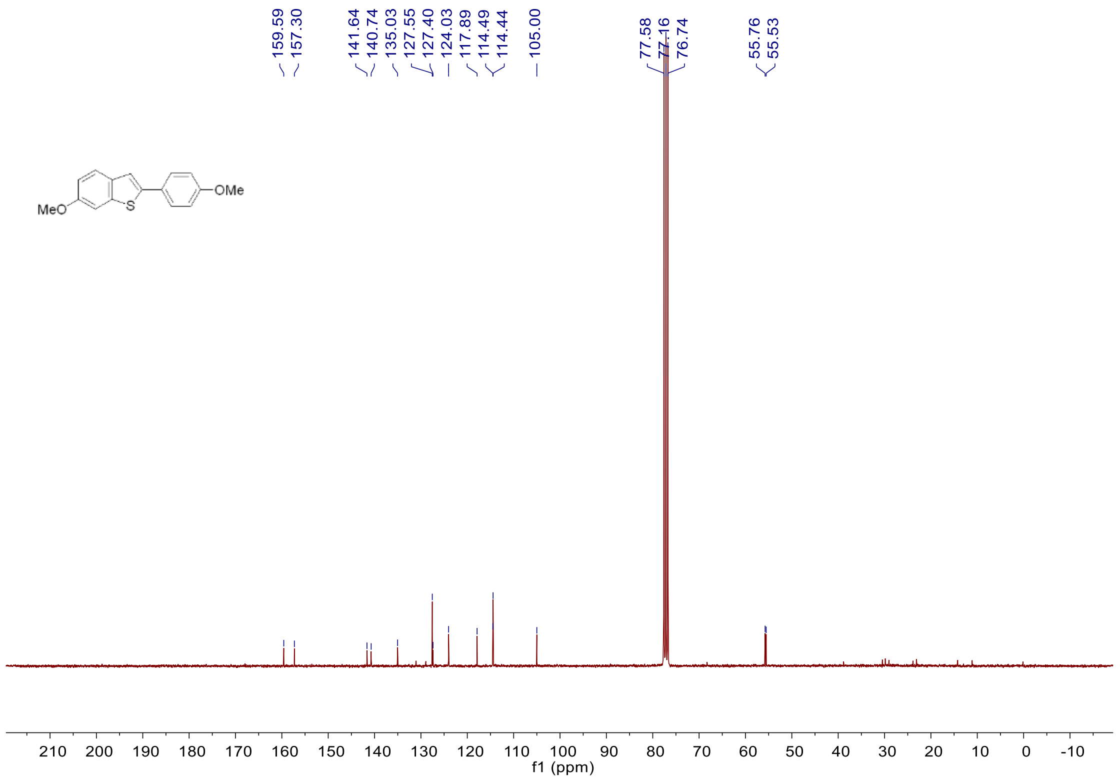

^{13}C NMR of **E**

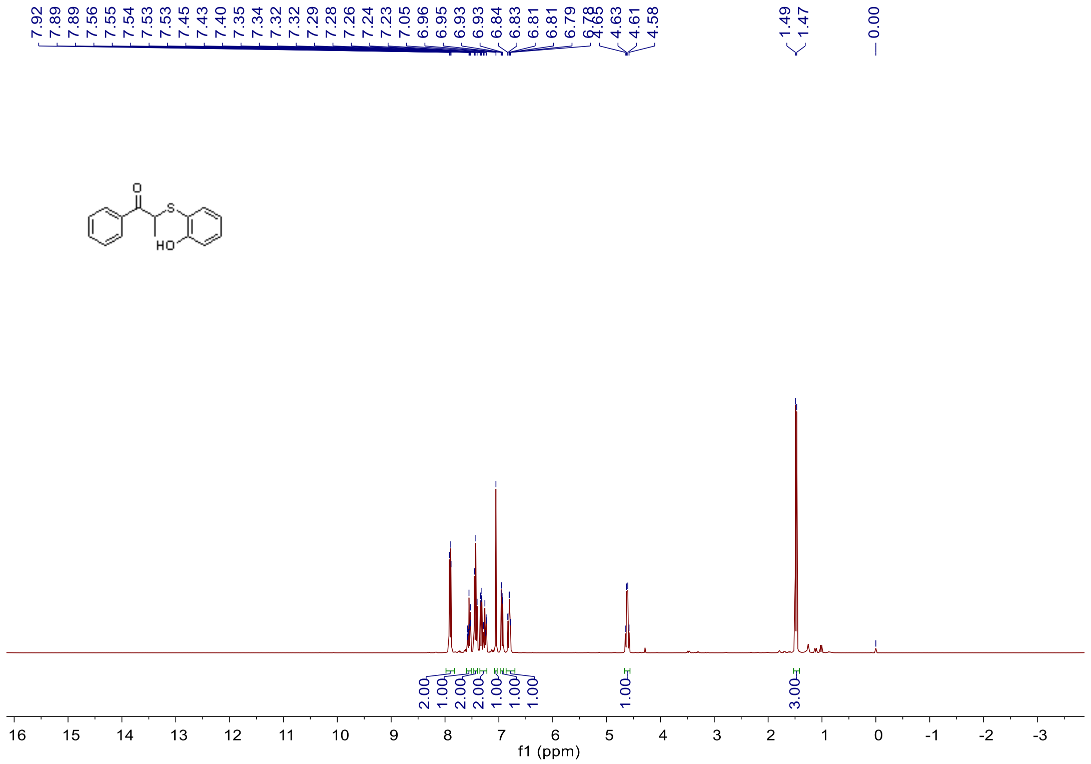

^{1}H NMR of **F**

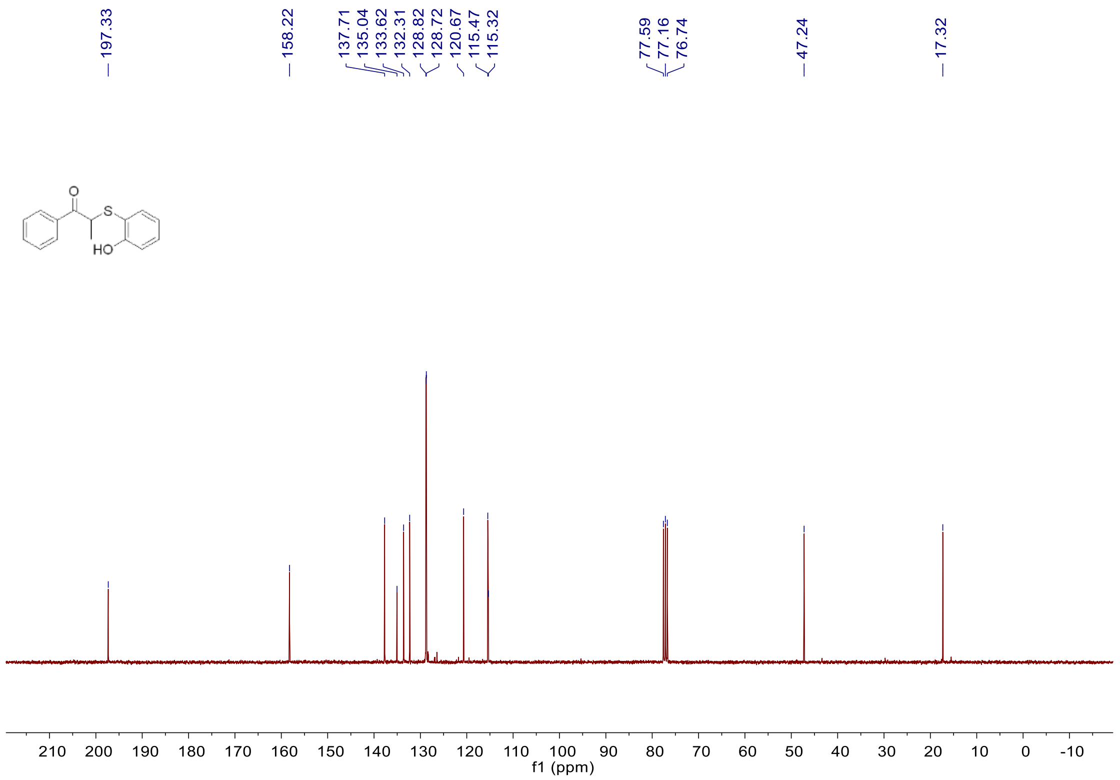

^{13}C NMR of **F**

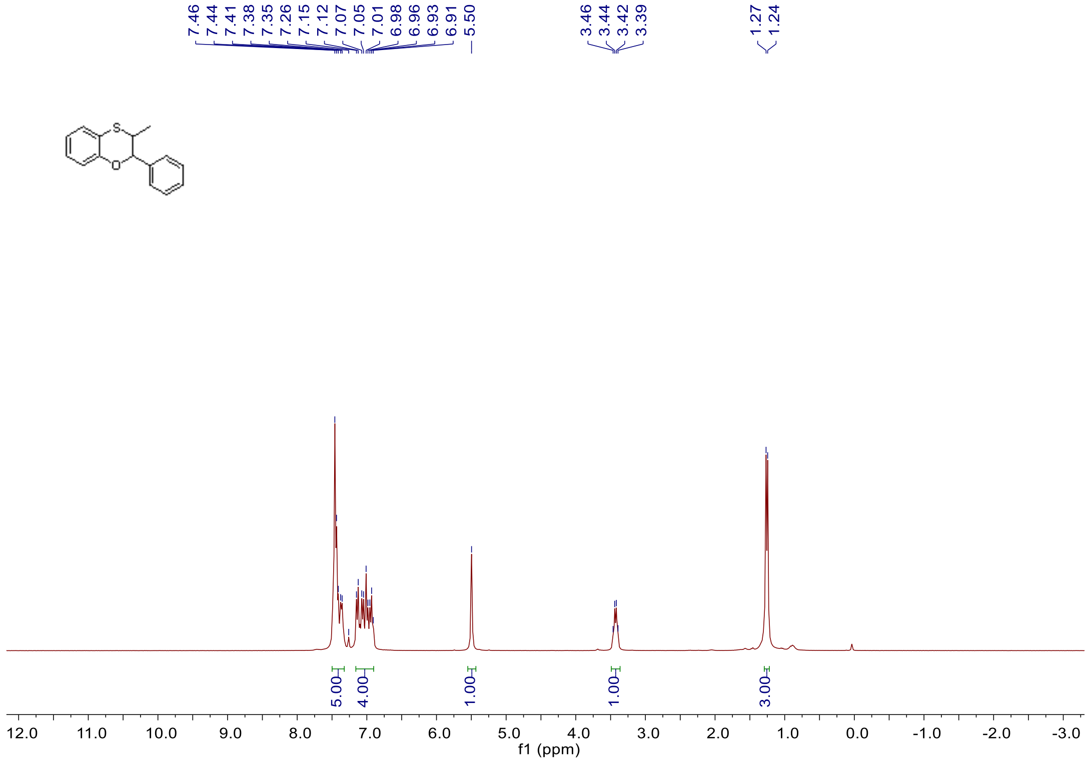

^{1}H NMR of **G**

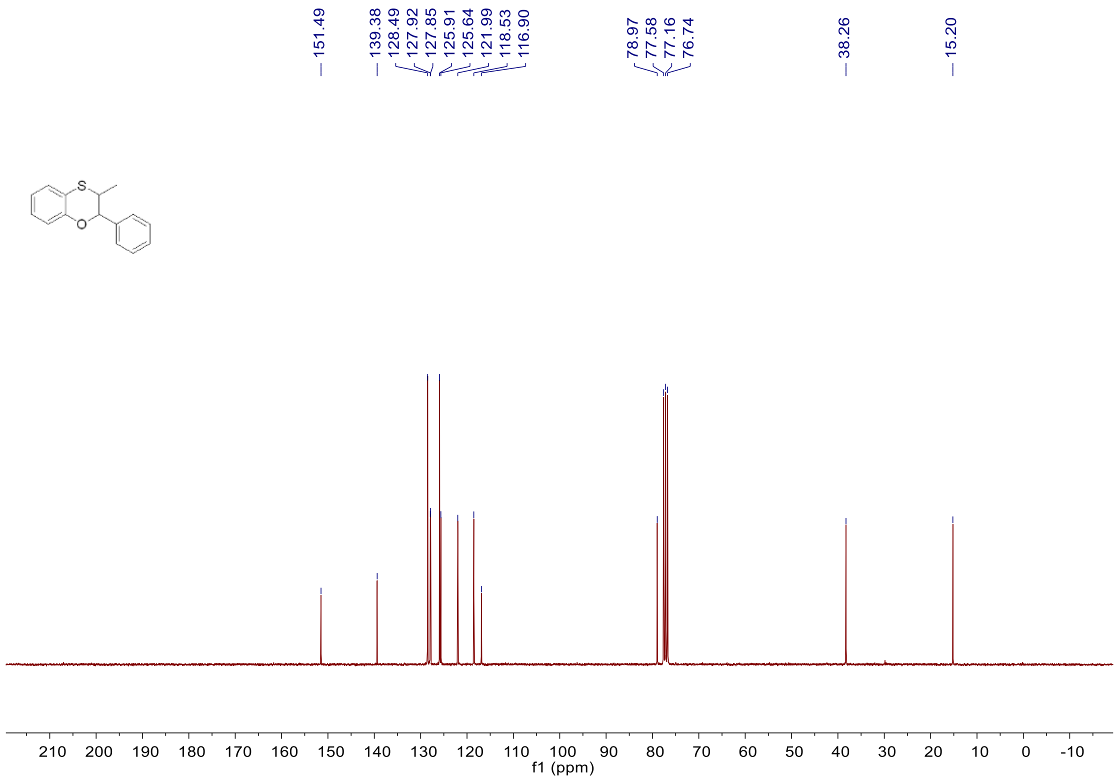

^{13}C NMR of **G**

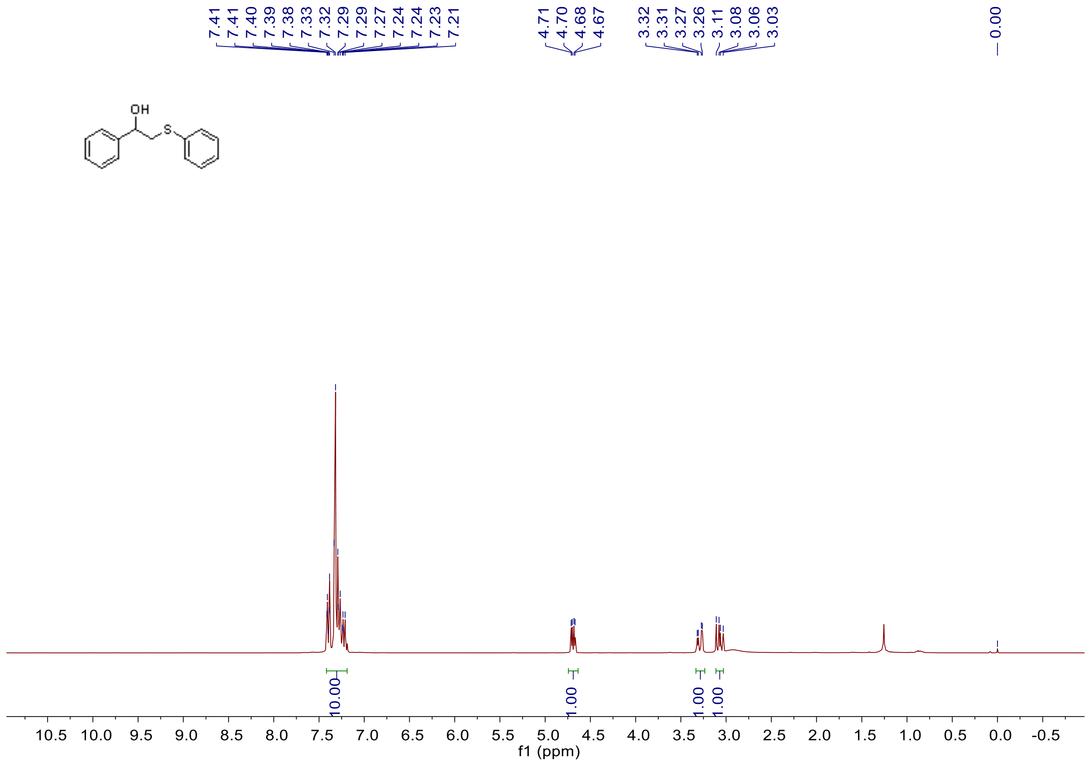

^{1}H NMR of **H**

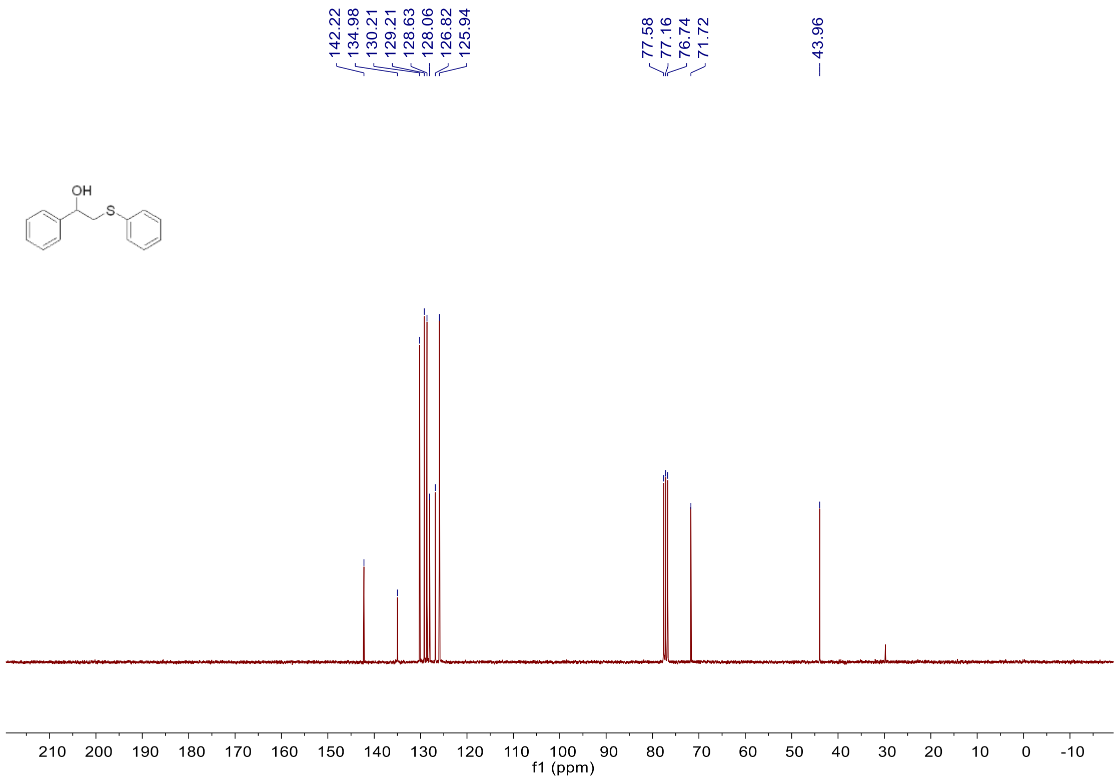

^{13}C NMR of **H**

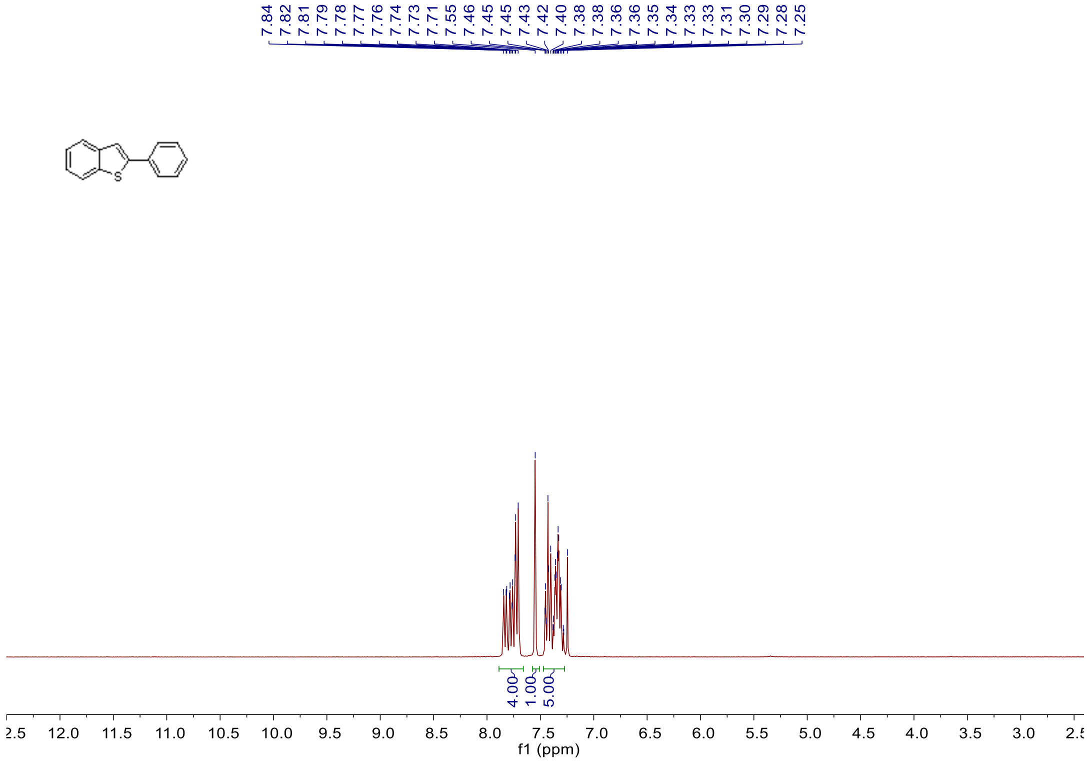

^{1}H NMR of **I**

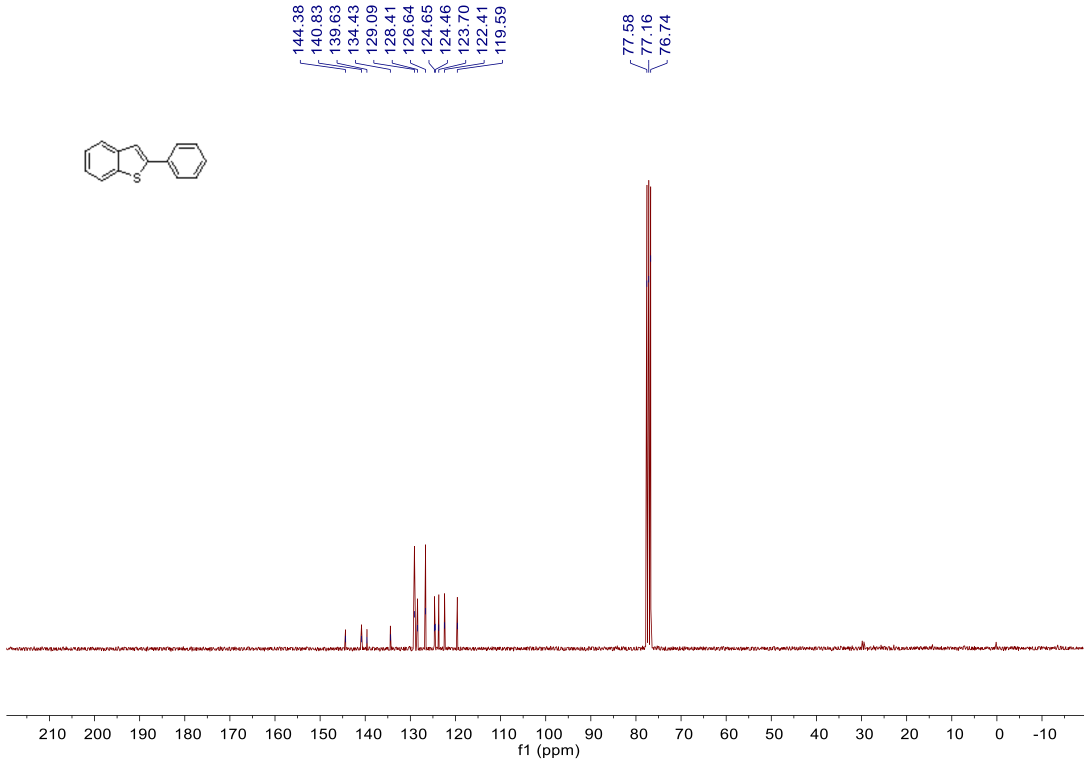

^{13}C NMR of **I**

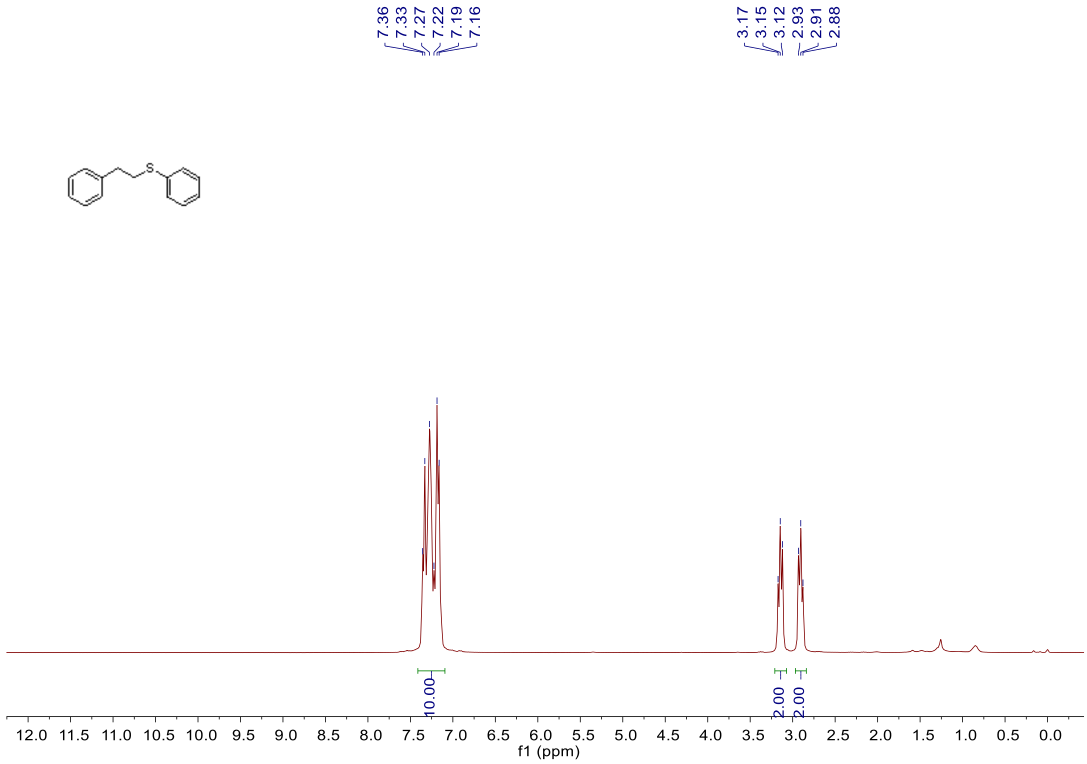

^{1}H NMR of **J**

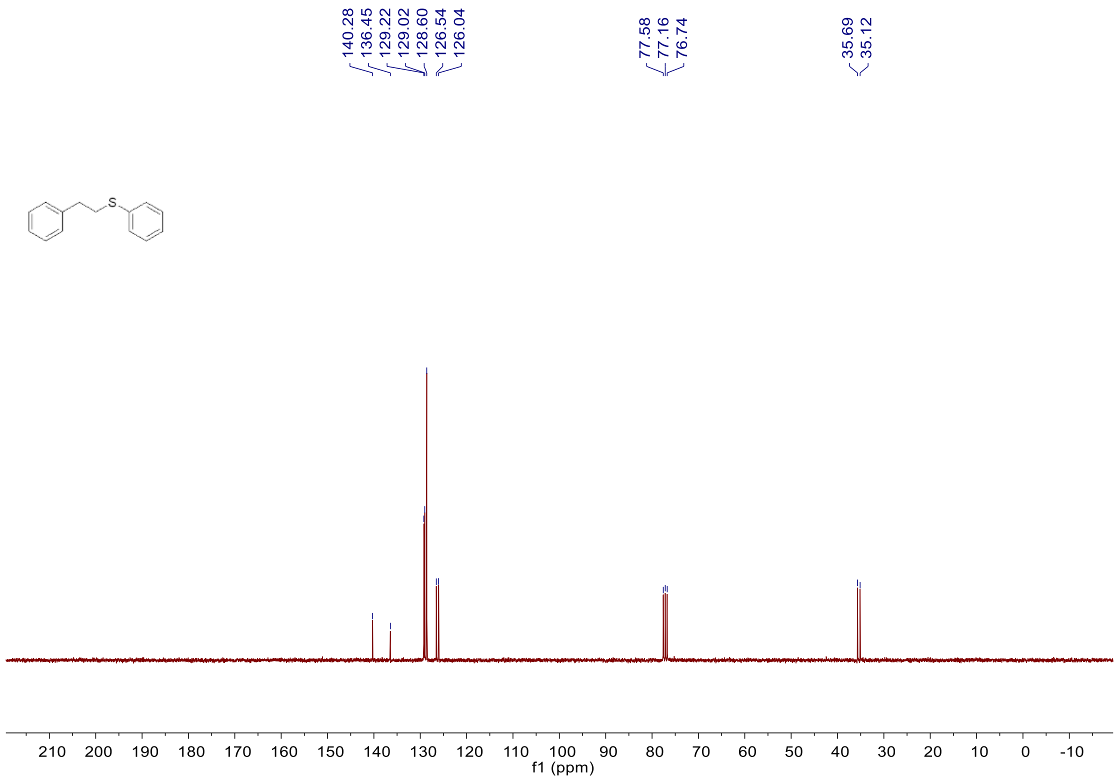

^{13}C NMR of **J**

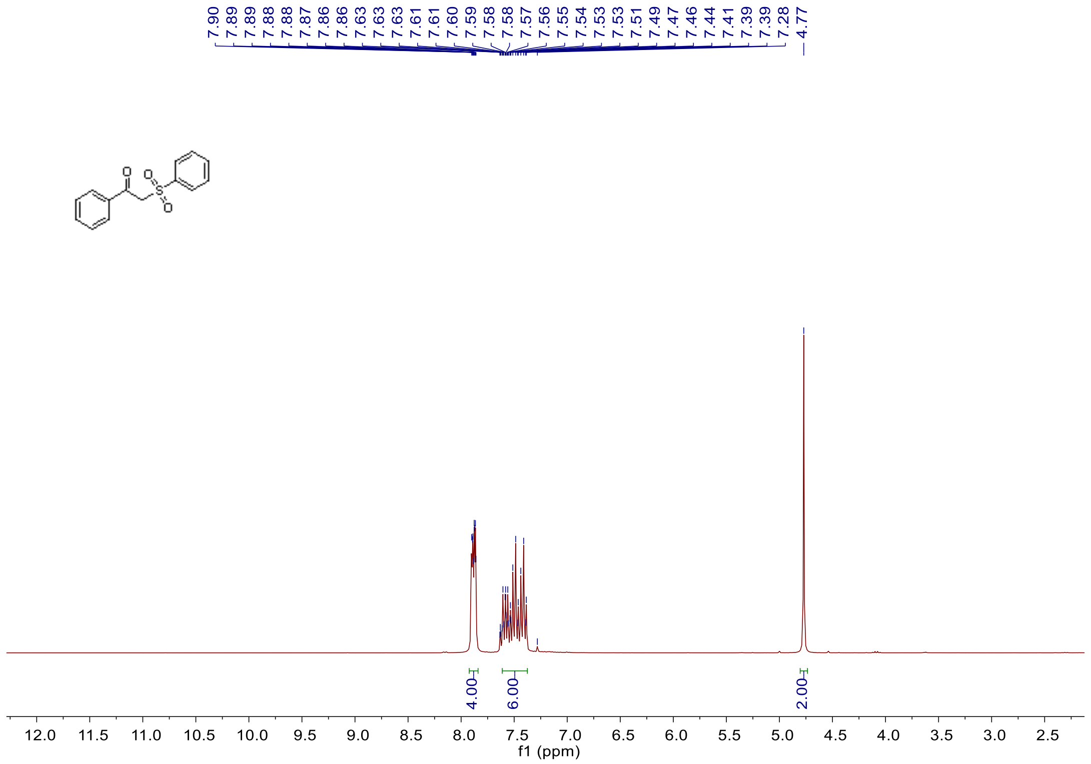

^{1}H NMR of **K**

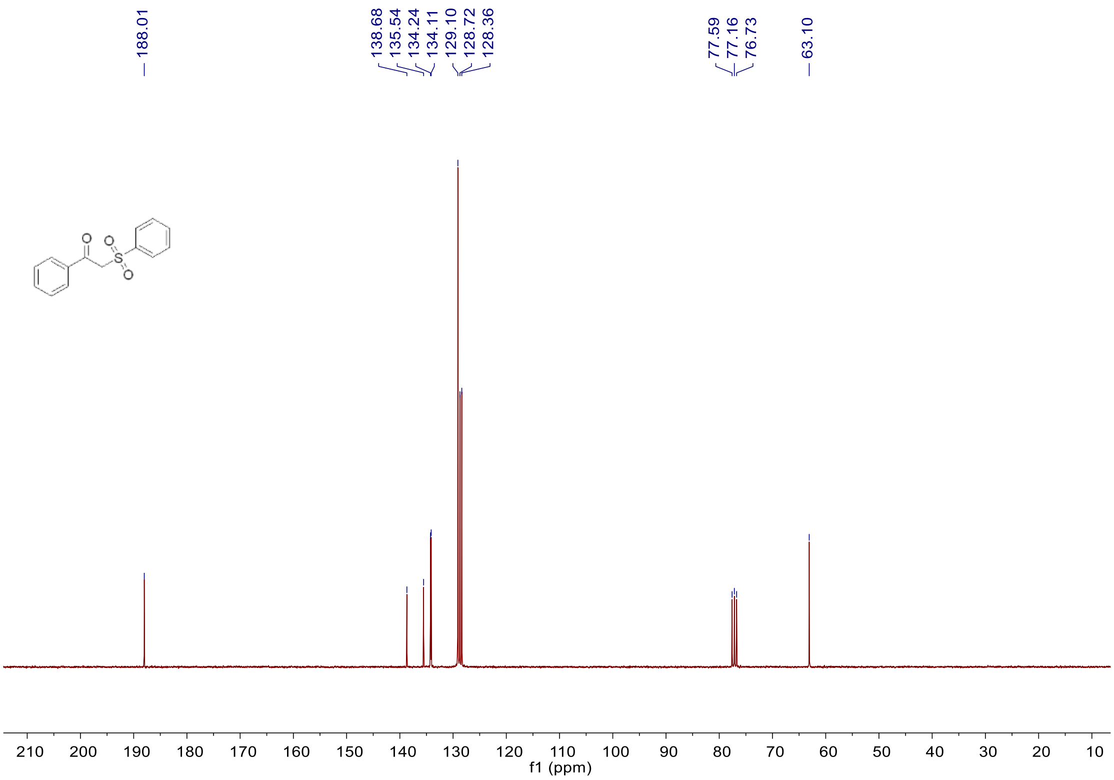

^{13}C NMR of **K**

References

(1) Zhao, F.; Lauder, K.; Liu, S. Y.; Finnigan, J. D.; Charnock, S. B. R.; Charnock, S. J.; Castagnolo, D. Chemoenzymatic Cascades for the Enantioselective Synthesis of *β*-Hydroxysulfides Bearing a Stereocentre at the C-O or C-S Bond by Ketoreductases. *Angew. Chem. Int. Ed.* **2022**, *61*, e202202363.

(2) Cellnik, T.; Healy, A. R. Sulfonyl Chlorides as Thiol Surrogates for Carbon-Sulfur BondFormation: One-Pot Synthesis of Thioethers and Thioesters. *J. Org. Chem.* **2022**, *87*, 6454-6458.

(3) Dias, R. M. P.; Burtoloso, A. C. B. Catalyst-Free Insertion of Sulfoxonium Ylides into Aryl Thiols. A Direct Preparation of *β*-Keto Thioethers. *Org. Lett.* **2016**, *18*, 3034-3037.

(4) Wang, Y.; Wang, Y. J.; Liang, X. C.; Shen, M. H.; Xu, H. D.; Xu, D. F. An Aryl Thiol-vinyl Azide Coupling Reaction and a Thiol-vinyl Zide Coupling/Cyclization Cascade: Efficient Synthesis of *β*-ketosulfides and Arene-fused 5-Methylene-2-pyrrolidinone derivatives. *Org. Biomol. Chem.* **2021**, *19*, 5169-5176.

(5) Li, H.; Xia, Z. M.; Li, L. W.; Zeng, J.; Lv, J.; Wang, H. F.; Gu, S. X.; Chen, F. Regioselective Cleavage and Reconfiguration of C-S Bonds with Diazo Compounds. *Org. Lett.* **2024**, *26*, 8405-8409.
